# Synthesis and In Vitro Anti-Influenza Virus Evaluation of Novel Sialic Acid (C-5 and C-9)-Pentacyclic Triterpene Derivatives †

**DOI:** 10.3390/molecules22071018

**Published:** 2017-06-22

**Authors:** Xu Han, Long-Long Si, Yong-Ying Shi, Zi-Bo Fan, Shou-Xin Wang, Zhen-Yu Tian, Man Li, Jia-Qi Sun, Ping-Xuan Jiao, Fu-Xiang Ran, Yong-Min Zhang, De-Min Zhou, Su-Long Xiao

**Affiliations:** 1 State Key Laboratory of Natural and Biomimetic Drugs, School of Pharmaceutical Sciences, Peking University, Beijing 100191, China; ax_han@126.com (X.H.); silonglong@bjmu.edu.cn (L.-L.S.); sygne24501@sina.com (Y.-Y.S.); fancyfzb@163.com (Z.-B.F.); shouxinwang@126.com (S.-X.W.); fashankc@163.com (Z.-Y.T.); 18811711762@163.com (M.L.); jiaqi.sun@pku.edu.cn (J.-Q.S.); 15022326072@163.com (P.-X.J.); rfx@bjmu.edu.cn (F.-X.R.); deminzhou@bjmu.edu.cn (D.-M.Z.); 2 Institut Parisien de Chimie Moléculaire, CNRS UMR 8232, Université Pierre & Marie Curie-Paris 6, 4 Place Jussieu, Paris 75005, France; yongmin.zhang@upmc.fr; 3 School of Pharmacy, Jining Medical University, Rizhao 276826, China

**Keywords:** pentacyclic triterpene, sialic acid, influenza virus, structure-activity relationship (SAR)

## Abstract

The emergence of drug resistant variants of the influenza virus has led to a great need to identify novel and effective antiviral agents. In our previous study, a series of sialic acid (C-2 and C-4)-pentacyclic triterpene conjugates have been synthesized, and a five-fold more potent antiviral activity was observed when sialic acid was conjugated with pentacyclic triterpene via C-4 than C-2. It was here that we further reported the synthesis and anti-influenza activity of novel sialic acid (C-5 and C-9)-pentacyclic triterpene conjugates. Their structures were confirmed by ESI-HRMS, ^1^H-NMR, and ^13^C-NMR spectroscopic analyses. Two conjugates (**26** and **42**) showed strong cytotoxicity to MDCK cells in the CellTiter-Glo assay at a concentration of 100 μM. However, they showed no significant cytotoxicity to HL-60, Hela, and A549 cell lines in MTT assay under the concentration of 10 μM (except compound **42** showed weak cytotoxicity to HL-60 cell line (10 μM, ~53%)). Compounds **20**, **28**, **36**, and **44** displayed weak potency to influenza A/WSN/33 (H1N1) virus (100 μM, ~20–30%), and no significant anti-influenza activity was found for the other conjugates. The data suggested that both the C-5 acetylamide and C-9 hydroxy of sialic acid were important for its binding with hemagglutinin during viral entry into host cells, while C-4 and C-2 hydroxy were not critical for the binding process and could be replaced with hydrophobic moieties. The research presented herein had significant implications for the design of novel antiviral inhibitors based on a sialic acid scaffold.

## 1. Introduction

Influenza is one of the most common and dangerous viral respiratory diseases; it affects three to five million cases of severe illness and leads to approximately 250,000 to 500,000 deaths every year worldwide [1,2]. Based on the antigenic major surface glycoproteins hemagglutinin (HA; 18 subtypes) and neuraminidase (NA; 11 subtypes), it is divided into different subtypes [3]. Currently available options to fight against the respiratory system attack by influenza A viruses include both vaccines and antiviral agents. However, the three types of available vaccines (inactivated influenza vaccine, live attenuated influenza vaccine, and recombinant influenza vaccine) have moderate efficacy that varies seasonally due to constant viral evolution [4]. Currently, two classes of anti-influenza drugs have been developed for interruption of specific processes in influenza infection. Amantadine and rimantadine target the M2 protein which is an ion channel allowing protons to move through the viral envelope to uncoat viral RNA, and thus block the release of viral RNA into the cytoplasm [5]. Oseltamivir (Tamiflu) and zanamivir (Relenza), on the other hand, target neuraminidase (NA) protein, inhibiting its enzymatic activity and causing the tethered progeny virus to be unable to escape from its host cells. However, the emergence of drug-resistant influenza viruses has limited the use of those drugs [6,7,8,9].

**Figure 1:**
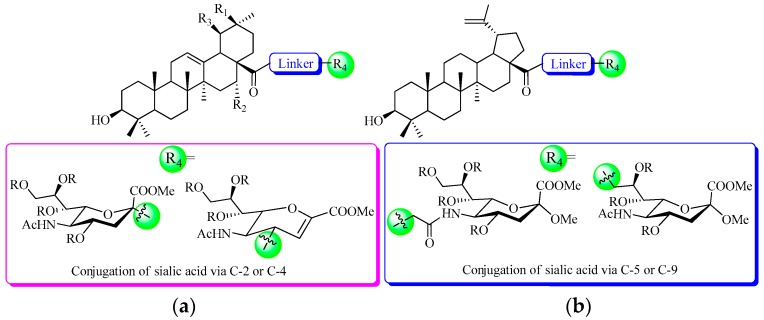
The structures of novel pentacyclic triterpene-sialic acid conjugates: (**a**) From previous work [25,26]; (**b**) In this work.

The first step of infection by influenza A virus is mediated by the interaction of sialic acid with the major surface viral glycoprotein haemagglutinin (HA) [10]. The crucial role of HA in the viral lifecycle makes it an attractive target for the development of therapeutics to treat influenza virus infection [11]. Sialic acid (also called as *N*-acetylneuraminic acid—Neu5Ac), a negatively charged nine-carbon carboxylated monosaccharide, is present at the non-reducing terminal positions of carbohydrate chains of glycoproteins and glycolipids on the cell surface [12]. It is well exploited by many pathogens to attach to and infect cells, and moreover, many pathogens decorate themselves with sialic acid to escape the host immune system [13]. In the last decades, many efforts have been made to develop an anti-influenza drug based on a sialic acid scaffold [14,15,16]. In fact, the analogs and derivatives of sialic acid have been shown to be potent inhibitors of influenza virus sialidase, and are commercially available [17]. Moreover, other bioactive molecules (such as cholesterol, [18] phospholipid, [19] taxol, [20] deazaflavin, [21] cytidine 5′-monophosphate, [22] and anthraquinone, [23]) have been selected to conjugate with sialic acid to prepare new biologically active molecules. Recently, we have explored potential agents against the influenza virus by the modifications at C-2 of the naturally sialic acid moiety (by conjugation of hydrophobic pentacyclic triterpene (Figure 1), which are widely distributed in the plant kingdom and generally believed to enhance the immunity of host plants and to increase plant resistance to pathogens [24]) with one compound exhibited moderate potency for influenza A/WSN/33 (H1N1) virus with IC_50_ at 41.2 μM [25]. More recently, a five-fold more potent anti-influenza activity was found when sialic acid was conjugated to pentacyclic triterpene via C-4 than to C-2 [26], possibly due to the fact that the big substitute group at C-2 of sialic acid affected the interactions of COOH with the basic amino acid of HA. 

As part of our continued interest in the structurally modified pentacyclic triterpene derivatives as an anti-influenza virus entry inhibitor, [25,27,28,29,30] we thought it of value to prepare a range of C-5 and C-9 modified sialic acid derivatives to better explore the antiviral structure-activity relationship (SAR) of sialic acid-pentacyclic triterpene conjugates. We reported herein the synthesis and anti-influenza A/WSN/33 virus activity of a series of C-5 and C-9 modified sialic acid conjugates of pentacyclic triterpene.

## 2. Results and Discussion

### 2.1. Chemistry

As outlined in our previous work [31], we have achieved the synthesis of the *N*-propargyl pentacyclic triterpene amides **15**–**17** and **27** as well as 1-benzotriazolyl pentacyclic triterpene esters **31**–**33** and **43**, respectively. 

**Scheme 1:**
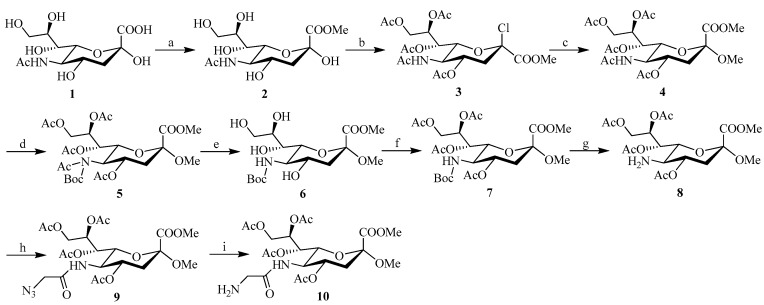
Reagents and conditions: (a) MeOH, H^+^-exchange resin, RT, 40 h; (b) AcCl, AcOH, MeOH, 24 h; (c) MeOH, RT, 1 h; then Ac_2_O, pyridine, RT, 24 h; (d) Boc_2_O, DMAP, THF; (e) MeONa/MeOH, 1 h; (f) pyridine, Ac_2_O; (g) TFA, DCM; (h) N_3_CH_2_COOH, EDC, THF; (i) H_2_, Pd/C, MeOH.

The C-5 functionalized sialic acid intermediates **9** and **10** were synthesized using the approach shown in Scheme 1. Commercially available sialic acid **1** was used as a starting material, and synthesis of the 4,7,8,9-tetra-*O*-acetyl-*N*-acetylneuraminic acid **4** was performed in moderate yield according to published procedures by Tropper et al. (Scheme 1) [32]. In order to synthesize the C-5 azide substituted intermediate **9** required for cycloaddition reaction, the NHAc group at C-5 was first Boc-protected by treatment with di-*tert*-butyl dicarbonate (Boc_2_O) in THF and then de-*O*-acetylated under Zemplén conditions [33] (followed by re-*O*-acetylation to provide derivative **7** in ~82% yield over three steps). Transformation to the azide substituted amide **9** was then performed by removal of the Boc group with TFA in DCM, followed by acylation of the amine. Subsequent catalytic reduction of the 2-azido-acetamide at position C-5 of **9** was achieved by exposure to a H_2_ atmosphere in the presence of 10% Pd/C in methanol, to afford the *N*-glycylneraminic acid intermediate **10** in 93% yield (which was used without further purification in the next step).

**Scheme 2:**
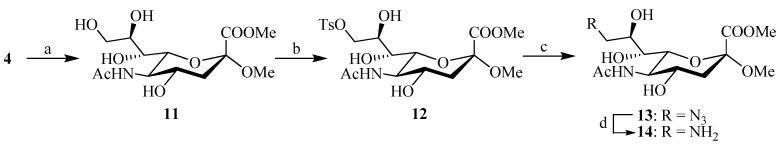
Reagents and conditions: (a) MeONa/MeOH, 3 h; (b) *p*-TsCl, pyridine; (c) DMF, NaN_3_, 60 °C, 24 h; (d) Pd/C, H_2_, CH_3_OH.

The synthesis of C-9 functionalized sialic acid derivatives **13** and **14** was accessed from intermediate **4** (Scheme 2). The acetyl groups of **4** were firstly removed under Zemplén conditions, followed by selective mono-tosylation of C-9 hydroxy to afford compound **12**. This compound was followed by nucleophilic substitution with sodium azide in DMF to provide the intermediate **13**, and further reduction of the azide group by hydrolygenolysis with Pd/C catalysis yielded the corresponding C-9 amine intermediate **14**, which was used without further purification in the next step.

**Scheme 3:**
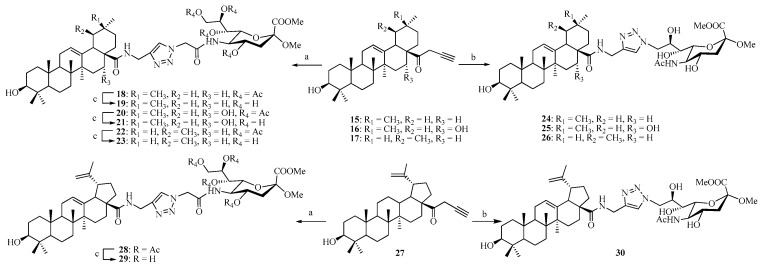
Reagents and conditions: (a) **9**, CuSO_4_, sodium l-ascorbate, DCM/H_2_O (1:1, *v*/*v*); (b) **13**, CuSO_4_, sodium l-ascorbate, DCM/H_2_O (1:1, *v*/*v*); (c) MeONa/MeOH, 1 h.

Coupling of compound **9** with *N*-propargyl triterpene amides **15**–**17** and **27** via click reaction yielded the acetyl-protected conjugates **18**, **20**, **22**, and **28**, respectively. All the click reactions were performed in DCM/H_2_O (1:1, *v*/*v*) at room temperature for 12–24 h. In all cases, pentacyclic triterpene-sialic acid conjugates were separated by extraction and purified by column chromatography on silica gel, and the yield after the purification was between 88% and 95%. In the next step, the Ac groups were removed under Zemplén conditions to give the corresponding conjugates **19**, **21**, **23,** and **29** as the final products quantitatively (Scheme 3). On the other hand, **13** (which does not contain any protecting groups on the hydroxyl groups) underwent click reaction with *N*-propargyl triterpene amides **15**–**17** and **27** under similar conditions to directly provide conjugates **24**–**26** and **30** in yields ranging from 79% to 87%.

**Scheme 4:**
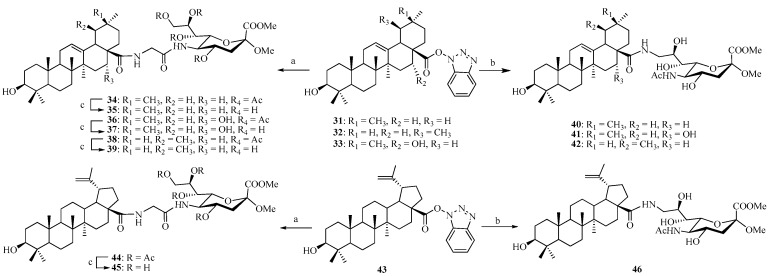
Reagents and conditions: (a) **10**, Na_2_CO_3_, DMF, 60 °C, 24 h; (b) **14**, Na_2_CO_3_, DMF, 60 °C, 24 h; (c) MeONa/MeOH, 1 h.

Alternatively, the 1-benzotriazolyl derivatives of pentacyclic triterpene **31**–**33** and **43** were coupled with intermediate **10** by route method in DMF. The crude products (compounds **34**, **36**, **38**, and **44**) were purified by column chromatography, and the coupling yields were good and ranged from 75% to 85% (Scheme 4). The Ac-removal of **34**, **36**, **38**, and **44** under Zemplén conditions gave the corresponding conjugates **35**, **37**, **39**, and **45**, quantitatively. Similarly coupling **31**–**33** and **43** with **14** gave the final conjugates **40**–**42** and **46**, respectively, with good yields ranging from 75% to 83%.

All novel conjugates gave satisfactory analysis by TLC and ^1^H- and ^13^C-NMR and HRMS.

### 2.2. Biological Evaluation

**Figure 2:**
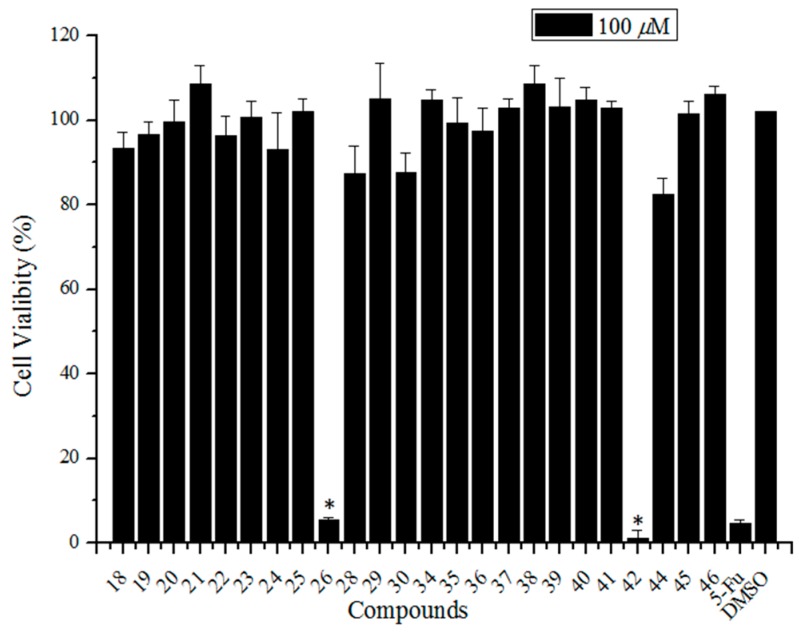
The cytotoxicity screening of sialic acid and pentacyclic triterpene conjugates (100 μM) using CellTiter-Glo^®^ Assay. DMSO and 5-Fu were used as negative and positive controls, respectively. Error bars indicate standard deviations of triplicate experiments. * compared with negative control, *p* < 0.05.

Since the synthesis of sialic acid derivatives having a pentacyclic triterpene substituent at the C-5 or C-9 positions were accomplished, our attention was directed to the biological activities of the anti-influenza virus. Initially, we examined the cytotoxicity of the newly synthesized compounds **18**–**26**, **28**–**30**, **34**–**42**, and **44**–**46** in Madin-Darby canine kidney (MDCK) cells using the CellTiter-Glo^®^ assay. A culture medium containing 0.5% DMSO served as a negative control. 5-Fluorouracil (5-Fu) (a well-known broad spectrum anticancer drug [34]) was selected as a positive control. Except for compounds **26** and **42** (two sialic acid (C-9)-ursolic acid conjugates), the other compounds showed no significant cytotoxicity to MDCK cells at a concentration of 100 μM (Figure 2).

The cytotoxicity of compounds **26** and **42** against human promyelocytic leukemia HL-60, human cervical cancer Hela, and human lung cancer A549, was further examined by the 3-(4,5-dimethylthiazol-2-yl)-2,5-diphenyltetrazolium bromide (MTT) assay with tested compound at a concentration of 0.1–10 μM [35]. With the exception of compound **42**, which showed weaker cytotoxicity against HL-60 (10 μM, 53%), no significant cytotoxicity was found (see Table S1 in Supplementary Materials). These results revealed that there was almost no cytotoxicity for those two ursolic acid-pentacyclic triterpene conjugates at a concentration of less than 10 μM. 

**Figure 3:**
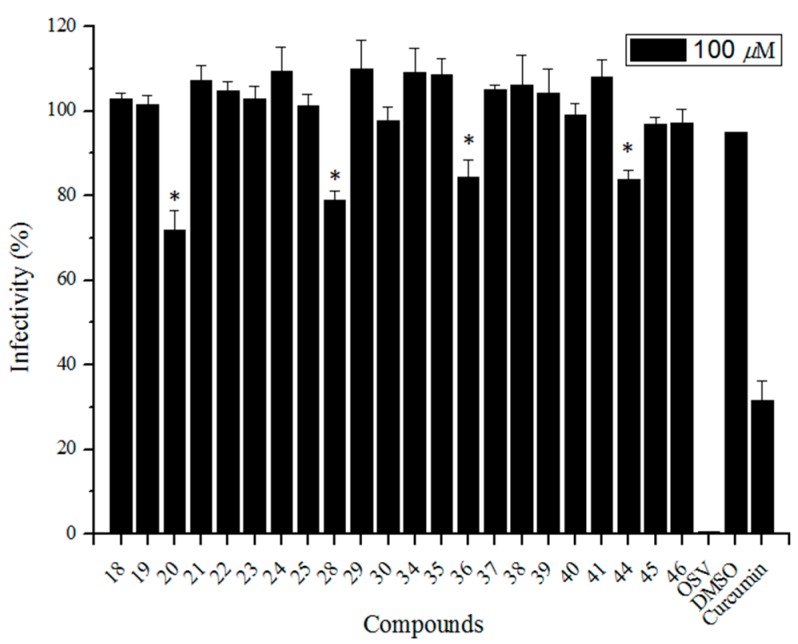
The cytopathic effect-based screening of conjugated sialic acid and pentacyclic triterpene derivatives. The Madin-Darby canine kidney (MDCK) was utilized as the host cell to test the A/WSN/33 virus infection; 0.5% DMSO (final concentration) was used as the negative control; curcumin (a small-molecule entry inhibitor targeting the HA1 domain) as well as OSV (oseltamivir) were utilized as positive controls. Error bars indicate standard deviations of triplicate experiments. * compared with negative control, *p* < 0.05.

Next, we examined the inhibitory activity of the test compounds against the virus replication in MDCK cells using the influenza A/WSN/33 (H1N1 subtype) virus strain at a concentration of 100 μM. The virus yields as a percent of control were estimated by a plaque titration method, and the results are shown in Figure 3 (including OSV and curcumin—a small-molecule entry inhibitor targeting the influenza virus HA1 domain [36] as positive controls). Four compounds **20**, **28**, **36**, and **44** (two of them are echinocystic acid-sialic acid (C-5)-echinocystic acid conjugates **20** and **36** and the other two are sialic acid (C-5)-betulinic acid conjugates) showed weak anti-influenza A/WSN/33 virus activity with IC_50_ > 100 μM. All the other compounds displayed no activity against influenza at high concentration. These data indicated that (1) the modification of the C-5 position of sialic acid showed a little more potent antiviral activity than the C-9 position; and (2) the introduction of large hydrophobic pentacyclic triterpene groups onto the C-5 acetamide or C-9 hydroxy of sialic acid might affect the interaction of sialic acid with HA during viral entry. The results of both the study described here and those previously reported by ours [25,26] demonstrated that the C-5 and C-9 positions of sialic acid were important for its binding with the HA protein (the introduction of a large hydrophobic group at those positions would affect their interaction), while the introduction of an appropriate hydrophobic group at the C-2 and C-4 positions of sialic acid could increase the binding with the active site of HA without detriment to binding affinity.

In order to determine their specificity for influenza virus infections, we further tested their inhibition activity on vesicular stomtatis virus G protein pseudo-particle (VSVpp), a negative-strand RNA virus with abroad host range that infects almost all cell lines. The results showed that they displayed no inhibition activity on VSV entry, indicating that they are specific anti-influenza entry inhibitors.

In our previous study, we found that certain pentacyclic triterpene displayed broad spectrum anti-influenza activity by blocking virus entry. The synergistic effect of compound **20** (the most potent of the four compounds), combined with OSV (an oral neuraminidase inhibitor), was investigated to explore the potential of the compound for use in cocktail therapy. We found that the addition of compound **20** to OSV increased its efficacy at inhibiting influenza infection. According to the median-effect equation, [37] moderate synergic anti-influenza effects were observed with the combination index at 0.83. The synergistic effect of pentacyclic triterpene with other anti-influenza virus inhibitors may provide a new option for the treatment of influenza virus infections.

## 3. Materials and Methods 

### 3.1. Chemistry

High-resolution mass spectra (HRMS) were obtained with an APEX IV FT_MS (7.0T) spectrometer (Bruker Daltonics, Inc., Billerica, MA, USA) in positive ESI mode. NMR spectra were recorded on a Bruker DRX 400 spectrometer at ambient temperature. ^1^H-NMR chemical shifts were referenced to the internal standard TMS (δ_H_ = 0.00) or the solvent signal (δ_H_ = 3.31 for the central line of CD_3_OD). ^13^C-NMR chemical shifts were referenced to the solvent signal (δ_C_ = 77.00 for the central line of CDCl_3_, δ_C_ = 49.00 for the central line of CD_3_OD). Reactions were monitored by thin-layer chromatography (TLC) on a pre-coated silica gel 60 F_254_ plate (layer thickness 0.2 mm; E. Merck, Darmstadt, Germany) and detected by staining with a yellow solution containing Ce(NH_4_)_2_(NO_3_)_6_ (0.5 g) and (NH_4_)_6_Mo_7_O_24_**^.^**4H_2_O (24.0 g) in 6% H_2_SO_4_ (500 mL), followed by heating. Flash column chromatography was performed on silica gel 60 (200–300 mesh, Qingdao Marine Chemical Co., Ltd., Qingdao, China).

All chemicals were used as supplied without further purification. The synthesis of compounds **2**–**8**, **11**–**17**, **27**, **31**–**33**, and **43** has been reported previously [31,32,38]. The intermediates **9** and **10** and the novel sialic acid (C-5 or C-9)-pentacyclic triterpene-Neu5Ac2en conjugates **18**–**26**, **28**–**30**, **34**–**42**, and **44**–**46** were obtained as follows.

#### 3.1.1. General Procedure A for the Click Reaction

CuSO_4_ (48 mg, 0.30 mmol) and sodium ascorbate (119 mg, 0.60 mmol) were added to a solution of alkyne (0.45 mmol) and azide (0.30 mmol) in DCM/H_2_O (1:1 *v*/*v*, 12 mL). The resulting solution was vigorously stirred at room temperature for 12 h. The reaction mixture was extracted with DCM (3 × 10 mL). The combined organic layer was dried over Na_2_SO_4_, filtered and concentrated. The residue was purified by column chromatography over silica gel.

#### 3.1.2. General Procedure B for the Deacetylation Reaction

The per-*O*-acetylated sialic acid (C-5 or C-9)-pentacyclic triterpene conjugate was dissolved in dry MeOH (~5 mL per 100 mg compound), and a solution of MeONa (30% in MeOH, 0.1 equiv mol^−1^ acetate) was added. The solution was stirred at room temperature for 3 h. After completion (TLC), the reaction mixture was neutralized with Amberlite IR-120 (H^+^) ion-exchange resin, filtered, and concentrated. The crude product was purified by column chromatography over silica gel.

#### 3.1.3. General Procedure C for the Amidation Reaction

Na_2_CO_3_ (201 mg, 1.9 mmol) was added to a solution of amino substituted sialic acid derivatives **10** or **14** (0.37 mmol) and 1-benzotriazolyl activated pentacyclic triterpene **31**–**33** or **43** (0.56 mmol) in DMF (20 mL). The resulting solution was vigorously stirred for 24 h at 60 °C. After completion (TLC), the reaction mixture was concentrated. The residue was purified by column chromatography over silica gel.

*Methyl (O-methyl-5-(N-azidoacetyl)-4,7,8,9-tetra-O-acetyl-3,5-dideoxy-d-glycero-α-d-galacto-2-nonulopyranosyl)onate* (**9**). EDC (62 mg, 0.32 mmol) was added to a solution of 2-azidoacetic acid (33 mg, 0.32 mmol) in dry THF. The resulting solution was vigorously stirred for 0.5 h at room temperature. Then compound **8** (100 mg, 0.22 mmol—dissolved in dry THF (10 mL)) was added. The solution was stirred at room temperature for another 24 h. After completion (TLC), the reaction mixture was concentrated, and the crude product was purified by column chromatography (eluent: PE/Act = 3:2) over silica gel to afford compound **9** as a white solid in 87% yield. *R*_f_ = 0.25 (PE/Act = 2:1); ^1^H-NMR (400 MHz, CDCl_3_): δ 6.18 (d, 1H, *J* = 10.2 Hz), 5.42–5.47 (m, 1H), 5.34 (dd, 1H, *J* = 2.3, 8.6 Hz), 4.91 (ddd, 1H, *J* = 12.2, 10.3, 4.6 Hz), 4.31 (dd, 1H, *J* = 2.6, 12.5 Hz), 4.21 (dd, 1H, *J* = 2.3, 10.7 Hz), 4.03–4.14 (m, 2H), 3.81–3.92 (m, 5H), 3.33 (s, 3H), 2.61 (dd, 1H, *J* = 4.7, 12.8 Hz), 2.18, 2.13, 2.04, 2.03 (s, 3H each, 4 × CH_3_CO), 1.94 (t, 1H, *J* = 12.6 Hz); ^13^C-NMR (100 MHz, CDCl_3_): δ 170.78, 170.26, 170.19, 168.12, 167.39, 99.05, 72.29, 68.92, 68.58, 67.24, 62.21, 52.84, 52.65, 52.51, 49.30, 37.96, 21.15, 20.88, 20.86, 20.78; ESI-HRMS calcd. for C_21_H_30_NaN_4_O_13_ [M + H]^+^: 569.1702, found 569.1708.

*Methyl (O-methyl-5-(N-aminoacetyl)-4,7,8,9-tetra-O-acetyl-3,5-dideoxy-d-glycero-α-d-galacto-2-nonulopyranosyl)onate* (**10**). The compound **9** (230 mg, 0.42 mmol) was dissolved in MeOH (15 mL), and palladium–carbon (0.1 equiv.) was added. The suspension was degassed under vacuum and urged with H_2_ three times; then it was stirred under an H_2_ balloon at room temperature for 24 h. The suspension was filtered through a pad of celite and the pad cake was washed with CH_3_OH. The combined filtrate was concentrated to dryness. The residue was used without further purification in the next step.

*Methyl (O-methyl-5-(N-(4-(N-(3β-hydroxy-olean-12-en-28-oyl)-amino)methyl)-1H-1,2,3-triazolyl)acetyl-4,7,8,9-tetra-O-acetyl-3,5-dideoxy-d-glycero-α-d-galacto-2-nonulopyranosyl)onate* (**18**). Prepared from **9** and **15** according to general procedure A, the residue was purified by chromatography (eluent: PE/Act = 1:1) over silica gel to afford compound **18** as a white solid in 93% yield. *R*_f_ = 0.25 (PE/Act = 1:1); ^1^H-NMR (400 MHz, CDCl_3_): δ 7.70 (s, 1H), 6.66 (t, 1H, *J* = 5.4 Hz), 6.45 (d, 1H, *J* = 9.8 Hz), 5.37–5.42 (m, 2H), 5.28–5.31 (m, 1H, overlap with DCM), 4.98 (d, 1H, *J* = 16.2 Hz), 4.75–4.82 (m, 2H), 4.60 (dd, 1H, *J* = 5.7, 15.0 Hz), 4.24–4.34 (m, 2H), 4.00–4.15 (m, 3H), 3.75 (s, 3H), 3.29 (s, 3H), 3.18–3.22 (m, 1H), 2.55 (dd, 1H, *J* = 4.6, 12.8 Hz), 2.13, 2.10, 2.03, 1.94 (s, 3H each, 4 × CH_3_CO), 2.01–0.93 (m, other aliphatic ring protons), 1.13, 0.97, 0.87, 0.86, 0.85, 0.76 (s, 3H each, 6 × CH_3_), 0.70 (d, 1H, *J* = 11.2 Hz), 0.60 (s, 3H, CH_3_); ^13^C-NMR (100 MHz, CDCl_3_): δ 178.51, 170.79, 170.72, 170.53, 170.23, 168.07, 165.72, 145.39, 144.47, 124.37, 123.25, 99.11, 79.06, 72.28, 68.74, 67.42, 62.38, 55.20, 52.85, 52.76, 52.56, 49.71, 47.65, 46.74, 46.31, 42.04, 39.46, 38.84, 38.57, 37.98, 37.05, 35.06, 34.19, 33.09, 32.57, 32.42, 30.80, 28.22, 27.39, 27.26, 25.91, 23.96, 23.71, 23.58, 21.24, 20.96, 20.91, 18.39, 16.69, 15.73, 15.48; ESI-HRMS calcd. for C_54_H_82_N_5_O_15_ [M + H]^+^: 1040.5802, found 1040.5798.

*Methyl (O-methyl-5-(N-(4-(N-(3β-hydroxy-olean-12-en-28-oyl)-amino)methyl)-1H-1,2,3-triazolyl)acetyl-3,5-dideoxy-d-glycero-α-d-galacto-2-nonulopyranosyl)onate* (**19**). Prepared from **18** according to general procedure B, the residue was purified by chromatography (eluent: DCM/MeOH = 10:1) over silica gel to afford compound **19** as a white solid in 83% yield. *R*_f_ = 0.24 (DCM/MeOH = 10:1); ^1^H-NMR (400 MHz, CD_3_OD): δ 7.85 (s, 1H), 7.73 (t, 1H, *J* = 5.3 Hz), 5.35 (brs, 1H), 5.17 (s, 2H), 4.40 (dq, 2H, *J* = 5.5, 15.1 Hz), 3.81–3.86 (m, 6H), 3.63–3.72 (m, 3H), 3.54 (d, 1H, *J* = 9.1 Hz), 3.34 (s, 3H), 3.14 (dd, 1H, *J* = 4.4, 11.2 Hz), 2.79 (d, 1H, *J* = 10.1 Hz), 2.66 (dd, 1H, *J* = 4.6, 12.8 Hz), 2.06 (dt, 1H, *J* = 2.8, 13.0 Hz), 1.88–1.89 (m, 2H), 1.00–1.81 (m, other aliphatic ring protons), 1.16, 0.97, 0.94, (s, 3H each, 3 × CH_3_), 0.92 (s, 6H, 2 × CH_3_), 0.78 (s, 3H, CH_3_), 0.73 (d, 1H, *J* = 11.2 Hz), 0.56 (s, 3H, CH_3_); ^13^C-NMR (100 MHz, CD_3_OD): δ 180.24, 170.71, 169.14, 146.16, 145.01, 126.17, 124.14, 100.27, 79.58, 74.42, 72.49, 70.05, 68.62, 64.71, 56.65, 53.94, 53.34, 52.95, 52.02, 48.94, 47.60, 47.41, 42.80, 42.53, 41.29, 40.55, 39.79, 38.07, 35.88, 35.05, 34.07, 33.72, 33.61, 31.59, 28.78, 28.43, 27.81, 26.55, 24.49, 24.08, 19.45, 17.46, 16.38, 15.97; ESI-HRMS calcd. for C_46_H_73_N_5_NaO_11_ [M + Na]^+^: 894.5199, found 894.5206.

*Methyl (O-methyl-5-(N-(4-(N-(3β,16α-dihydroxy-olean-12-en-28-oyl)-amino)methyl)-1H-1,2,3-triazolyl)acetyl-4,7,8,9-tetra-O-acetyl-3,5-dideoxy-d-glycero-α-d-galacto-2-nonulopyranosyl)onate* (**20**). Prepared from **9** and **16** according to general procedure A, the residue was purified by chromatography (eluent: PE/Act = 1:1) over silica gel to afford compound **20** as a white solid in 91% yield. *R*_f_ = 0.22 (PE/Act = 1:1); ^1^H-NMR (400 MHz, CDCl_3_): δ 7.73 (s, 1H), 6.94–6.97 (m, 2H), 5.53 (brs, 1H), 5.39–5.44 (m, 1H), 5.34 (dd, 1H, *J* = 3.1, 8.0 Hz), 4.92 (d, 1H, *J* = 16.3 Hz), 4.80 (dt, 1H, *J* = 4.5, 12.0 Hz), 4.71 (d, 1H, *J* = 16.3 Hz), 4.59 (dd, 1H, *J* = 5.7, 15.0 Hz), 4.34–4.38 (m, 2H), 4.23 (dd, 1H, *J* = 5.0, 15.0 Hz), 4.17 (dd, 1H, *J* = 2.0, 10.7 Hz), 4.03–4.12 (m, 2H), 3.79 (s, 3H), 3.30 (s, 3H), 3.20–3.22 (m, 1H), 2.73 (dd, 1H, *J* = 3.1, 13.4 Hz), 2.56 (dd, 1H, *J* = 4.6, 12.8 Hz), 2.52 (d, 1H, *J* = 4.6 Hz), 2.25 (t, 1H, *J* = 13.2 Hz), 2.12, 2.09, 2.02, 1.95 (s, 3H each, 4 × CH_3_CO), 1.36, 0.98 (s, 3H each, 2 × CH_3_), 1.00–2.07 (m, other aliphatic ring protons), 0.89 (s, 9H, 3 × CH_3_), 0.77 (s, 3H, CH_3_), 0.73 (d, 1H, *J* = 11.5 Hz), 0.69 (s, 3H, CH_3_); ^13^C-NMR (100 MHz, CDCl_3_): δ 178.42, 170.90, 170.74, 170.70, 170.18, 168.25, 165.88, 144.96, 143.77, 124.22, 123.66, 99.19, 79.02, 75.61, 72.45, 68.88, 68.80, 67.67, 62.51, 55.31, 52.91, 52.60, 52.52, 49.60, 49.10, 47.06, 46.83, 41.89, 41.57, 39.70, 38.88, 38.69, 38.10, 37.07, 35.41, 35.29, 35.06, 32.73, 32.59, 30.38, 29.86, 28.21, 27.31, 26.89, 25.10, 23.51, 21.26, 20.97, 20.94, 20.89, 18.37, 16.88, 15.75, 15.72; ESI-HRMS calcd. for C_54_H_81_N_5_NaO_16_ [M + Na]^+^: 1078.5571, found 1078.5580.

*Methyl (O-methyl-5-(N-(4-(N-(3β,16α-dihydroxy-olean-12-en-28-oyl)-amino)methyl)-1H-1,2,3-triazolyl)acetyl-3,5-dideoxy-d-glycero-α-d-galacto-2-nonulopyranosyl)onate* (**21**). Prepared from **20** according to general procedure B, the residue was purified by chromatography (eluent: DCM/MeOH = 10:1) over silica gel to afford compound **21** as a white solid in 87% yield. *R*_f_ = 0.21 (DCM/MeOH = 10:1); ^1^H-NMR (400 MHz, CD_3_OD): δ 7.84 (s, 1H), 7.56 (t, 1H, *J* = 5.3 Hz), 5.46 (brs, 1H), 5.17 (s, 2H), 4.31–4.43 (m, 3H), 3.81–3.87 (m, 6H), 3.62–3.71 (m, 3H), 3.53 (d, 1H, *J* = 9.1 Hz), 3.34 (s, 3H), 3.15 (dd, 1H, *J* = 5.0, 11.1 Hz), 2.86 (dd, 1H, *J* = 3.6, 13.6 Hz), 2.66 (dd, 1H, *J* = 4.7, 12.8 Hz), 2.36 (t, 1H, *J* = 13.2 Hz), 1.89–1.98 (m, 4H), 1.74 (t, 1H, *J* = 12.3 Hz), 1.01–1.65 (m, other aliphatic ring protons), 1.37, 0.97, 0.96, 0.92, 0.88, 0.78 (s, 3H each, 6 × CH_3_), 0.74 (d, 1H, *J* = 11.2 Hz), 0.57 (s, 3H, CH_3_); ^13^C-NMR (100 MHz, CD_3_OD): δ 180.11, 170.75, 169.16, 145.96, 144.93, 126.07, 124.25, 100.30, 79.63, 75.63, 74.47, 72.51, 70.08, 68.66, 64.75, 56.76, 53.96, 53.34, 52.96, 52.03, 49.90, 48.12, 48.06, 42.75, 42.30, 41.33, 40.71, 39.91, 39.82, 38.07, 36.37, 36.02, 35.95, 33.89, 33.31, 31.97, 31.24, 28.73, 27.87, 27.30, 25.33, 24.46, 19.44, 17.54, 16.36, 16.17; ESI-HRMS calcd. for C_46_H_73_N_5_NaO_12_ [M + Na]^+^: 910.5148, found 910.5152.

*Methyl (O-methyl-5-(N-(4-(N-(3β-hydroxy-urs-12-en-28-oyl)-amino)methyl)-1H-1,2,3-triazolyl)acetyl-4,7,8,9-tetra-O-acetyl-3,5-dideoxy-d-glycero-α-d-galacto-2-nonulopyranosyl)onate* (**22**). Prepared from **9** and **17** according to general procedure A, the residue was purified by chromatography (eluent: PE/Act = 1:1) over silica gel to afford compound **22** as a white solid in 95% yield. *R*_f_ = 0.25 (PE/Act = 1:1); ^1^H-NMR (400 MHz, CDCl_3_): δ 7.69 (s, 1H), 6.67 (t, 1H, *J* = 5.2 Hz), 6.58 (d, 1H, *J* = 9.8 Hz), 5.37–5.41 (m, 1H), 5.29–5.31 (m, 2H), 4.97 (d, 1H, *J* = 16.2 Hz), 4.75–4.82 (m, 2H), 4.58 (dd, 1H, *J* = 5.8, 15.0 Hz), 4.32 (dd, 1H, *J* = 2.5, 12.4 Hz), 4.25 (dd, 1H, *J* = 4.8, 15.0 Hz), 4.00–4.15 (m, 3H), 3.74 (s, 3H), 3.28 (s, 3H), 3.17–3.21 (m, 1H), 2.55 (dd, 1H, *J* = 4.7, 12.8 Hz), 2.12, 2.09, 2.02, 1.93 (s, 3H each, 4 × CH_3_CO), 0.97–1.98 (m, other aliphatic ring protons), 1.06, 0.96, 0.91, 0.86 (s, 3H each, 4 × CH_3_), 0.83 (d, 3H, *J* = 6.4 Hz, CH_3_), 0.75 (s, 3H, CH_3_), 0.69 (d, 1H, *J* = 11.8 Hz), 0.61 (s, 3H, CH_3_); ^13^C-NMR (100 MHz, CDCl_3_): δ 178.38, 170.76, 170.69, 170.54, 170.22, 168.07, 165.78, 145.27, 139.26, 126.17, 124.19, 99.09, 79.06, 72.26, 68.78, 68.72, 67.41, 62.35, 55.18, 53.56, 52.83, 52.67, 52.55, 49.62, 47.70, 47.62, 42.43, 39.77, 39.59, 39.05, 38.81, 38.69, 37.96, 37.14, 37.00, 34.99, 32.76, 30.92, 28.24, 27.90, 27.26, 24.99, 23.40, 21.31, 21.23, 20.95, 20.90, 18.35, 17.28, 16.66, 15.78, 15.57; ESI-HRMS calcd. for C_54_H_82_N_5_O_15_ [M + H]^+^: 1040.5802, found 1040.5807.

*Methyl (O-methyl-5-(N-(4-(N-(3β-hydroxy-urs-12-en-28-oyl)-amino)methyl)-1H-1,2,3-triazolyl)acetyl-3,5-dideoxy-d-glycero-α-d-galacto-2-nonulopyranosyl)onate* (**23**). Prepared from **22** according to general procedure B, the residue was purified by chromatography (eluent: DCM/MeOH = 10:1) over silica gel to afford compound **22** as a white solid in 89% yield. *R*_f_ = 0.25 (DCM/MeOH = 10:1); ^1^H-NMR (400 MHz, CD_3_OD): δ 7.84 (s, 1H), 7.65 (t, 1H, *J* = 5.4 Hz), 5.33 (brs, 1H), 5.16 (s, 2H), 4.33–4.43 (m, 2H), 3.82–3.87 (m, 6H), 3.62–3.72 (m, 3H), 3.54 (d, 1H, *J* = 9.1 Hz), 3.34 (s, 3H, overlap with MeOH), 3.15 (dd, 1H, *J* = 4.9, 11.0 Hz), 2.66 (dd, 1H, *J* = 4.6, 12.7 Hz), 2.14 (d, 1H, *J* = 10.7 Hz), 2.12 (dt, 1H, *J* = 3.8, 13.2 Hz), 1.90–1.91 (m, 2H), 1.10–1.78 (m, other aliphatic ring protons), 1.11 (s, 3H, CH_3_), 0.97 (s, 6H, 2 × CH_3_), 0.93 (s, 3H, CH_3_), 0.90 (d, 3H, *J* = 6.4 Hz, CH_3_), 0.78 (s, 3H, CH_3_), 0.73 (d, 1H, *J* = 11.2 Hz), 0.58 (s, 3H, CH_3_); ^13^C-NMR (100 MHz, CD_3_OD): δ 180.12, 170.72, 169.14, 146.12, 139.68, 127.32, 126.11, 100.29, 79.59, 74.43, 72.51, 70.07, 68.65, 64.73, 56.64, 54.14, 53.95, 53.34, 52.98, 52.03, 48.91, 43.19, 41.31, 40.78, 40.23, 39.93, 39.80, 38.49, 38.03, 35.83, 34.07, 31.86, 28.90, 28.80, 27.85, 25.27, 24.32, 24.13, 21.62, 19.41, 17.76, 17.50, 16.43, 16.11; ESI-HRMS calcd. for C_46_H_73_N_5_NaO_11_ [M + Na]^+^: 894.5199, found 894.5198.

*Methyl (O-methyl-9-((4-(N-(3β-hydroxy-olean-12-en-28-oyl)-amino)methyl)-1H-1,2,3-triazolyl)-5-acetamido-3,5-dideoxy-d-glycero-α-d-galacto-2-nonulopyranosyl)onate* (**24**). Prepared from **13** and **15** according to general procedure A, the residue was purified by column chromatography (eluent: DCM/MeOH = 15:1) over silica gel to afford compound **24** as a white solid in 83% yield. *R*_f_ = 0.29 (DCM/MeOH = 12:1); ^1^H-NMR (400 MHz, CD_3_OD): δ 7.79 (s, 1H), 7.70 (t, 1H, *J* = 5.5 Hz), 5.31 (t, 1H, *J* = 2.9 Hz), 4.78 (dd, 1H, *J* = 2.4, 14.1 Hz), 4.30–4.40 (m, 3H), 4.10 (dt, 1H, *J* = 2.2, 8.4 Hz), 3.78 (s, 3H), 3.73 (t, 1H, *J* = 10.3 Hz), 3.54–3.63 (m, 2H), 3.37–3.39 (m, 1H), 3.10 (dd, 1H, *J* = 4.5, 10.7 Hz), 2.76 (dd, 1H, *J* = 3.7, 13.3 Hz), 2.60 (dd, 1H, *J* = 4.5, 12.7 Hz), 1.96 (s, 3H), 0.68–2.05 (m, other aliphatic ring protons), 1.12, 0.93, 0.91, 0.88, 0.87,0.74, 0.54 (s, 3H each, 7 × CH_3_); ^13^C-NMR (100 MHz, CD_3_OD): δ 180.41, 175.20, 170.72, 145.08, 125.68, 124.22, 100.41, 79.67, 74.68, 71.61, 71.33, 68.41, 56.69, 54.79, 53.82, 53.35, 52.11, 47.60, 47.51, 42.87, 42.62, 41.39, 40.61, 39.83, 38.11, 35.98, 35.06, 34.14, 33.79, 33.54, 31.62, 28.73, 28.47, 27.85, 26.44, 24.52, 24.03, 22.68, 19.46, 17.54, 16.34, 16.16, 15.93; ESI-HRMS calcd. for C_46_H_74_O_10_N_5_ [M + H]^+^: 856.5430, found 856.5415.

*Methyl (O-methyl-9-((4-(N-(3β,16α-dihydroxy-olean-12-en-28-oyl)-amino)methyl)-1H-1,2,3-triazolyl)-5-acetamido-3,5-dideoxy-d-glycero-α-d-galacto-2-nonulopyranosyl)onate* (**25**). Prepared from **13** and **16** according to general procedure A, the residue was purified by column chromatography (eluent: DCM/MeOH = 18:1) over silica gel to afford compound **25** as a white solid in 81% yield. *R*_f_ = 0.27 (DCM/MeOH = 12:1); ^1^H-NMR (400 MHz, CD_3_OD): δ 7.78 (s, 1H), 7.53 (t, 1H, *J* = 5.3 Hz), 5.43 (t, 1H, *J* = 3.2 Hz), 4. 82 (dd, 1H, *J* = 2.4, 14.0 Hz), 4.27–4.38 (m, 4H), 4.11 (dt, 1H, *J* = 2.4, 8.4 Hz), 3.79 (s, 3H), 3.74 (t, 1H, *J* = 5.6 Hz), 3.54–3.63 (m, 2H), 3.37 (dd, 1H, *J* = 1.5, 8.4 Hz), 3.11 (dd, 1H, *J* = 5.1, 11.7 Hz), 2.84 (dd, 1H, *J* = 3.7, 13.5 Hz), 2.60 (dd, 1H, *J* = 4.6, 12.7 Hz), 2.32 (t, 1H, *J* = 13.7 Hz), 2.11 (s, 1H), 1.96 (s, 3H), 0.91–1.98 (m, other aliphatic ring protons), 1.13, 0.93, 0.92, 0.89, 0.85, 0.74 (s, 3H each, 6 × CH_3_), 0.74 (d, 1H, *J* = 11.4 Hz), 0.56 (s, 3H, CH_3_); ^13^C-NMR (100 MHz, CD_3_OD): δ 180.19, 175.21, 170.73, 145.73, 144.95, 125.50, 124.31, 100.41, 79.65, 75.60, 74.68, 71.61, 71.33, 68.41, 56.79, 54.79, 53.83, 53.36, 52.12, 49.98, 48.16, 48.03, 42.81, 42.37, 41.38, 40.75, 39.93, 39.84, 38.09, 36.35, 36.12, 35.99, 33.96, 33.28, 31.86, 31.24, 28.71, 27.90, 27.28, 25.36, 24.47, 22.69, 19.45, 17.61, 16.34, 16.15; ESI-HRMS calcd. for C_46_H_74_O_11_N_5_ [M + H]^+^: 872.5379, found 872.5360.

*Methyl (O-methyl-9-((4-(N-(3β-hydroxy-urs-12-en-28-oyl)-amino)methyl)-1H-1,2,3-triazolyl)-5-acetamido-3,5-dideoxy-d-glycero-α-d-galacto-2-nonulopyranosyl)onate* (**26**). Prepared from **13** and **17** according to general procedure A, the residue was purified by column chromatography (eluent: DCM/MeOH = 12:1) over silica gel to afford compound **26** as a white solid in 87% yield. *R*_f_ = 0.30 (DCM/MeOH = 12:1); ^1^H-NMR (400 MHz, CD_3_OD): δ 7.78 (s, 1H), 7.59 (t, 1H, *J* = 4.9 Hz), 5.29 (brs, 1H), 4.46–4.78 (m, 1H, overlap with H_2_O), 4.33–4.38 (m, 3H), 4.11 (dt, 1H, *J* = 1.6, 8.1 Hz), 3.79 (s, 3H), 3.73 (t, 1H, *J* = 9.8 Hz), 3.54–3.63 (m, 2H), 3.37 (d, 1H, *J* = 8.4 Hz), 3.11 (dd, 1H, *J* = 4.9, 11.4 Hz), 2.60 (dd, 1H, *J* = 4.6, 12.7 Hz), 2.10 (d, 1H, *J* = 10.8 Hz), 2.03 (dt, 1H, *J* = 3.4, 12.2 Hz), 1.96 (s, 3H), 0.56–1.89 (m, other aliphatic ring protons), 1.07 (s, 3H, CH_3_), 0.93 (s, 6H, 2 × CH_3_), 0.90 (s, 3H, CH_3_), 0.86 (d, 3H, *J* = 6.3 Hz, CH_3_), 0.74 (s, 3H, CH_3_), 0.69 (d, 1H, *J* = 11.4 Hz), 0.56 (s, 3H, CH_3_); ^13^C-NMR (100 MHz, CD_3_OD): δ 180.20, 180.12, 175.16, 170.71, 139.77, 127.36, 125.54, 100.39, 79.63, 74.67, 71.54, 71.3, 68.38, 56.67, 54.76, 54.22, 53.82, 53.35, 52.11, 48.36, 43.25, 41.37, 40.81, 40.28, 39.96, 39.82, 38.53, 38.06, 35.94, 35.83, 34.13, 31.88, 28.92, 28.78, 27.89, 25.28, 24.34, 24.09, 22.7, 21.59, 19.42, 17.72, 17.57; ESI-HRMS calcd. for C_46_H_74_O_10_N_5_ [M + H]^+^: 856.5430, found 856.5412.

*Methyl (O-methyl-5-(N-(4-(N-(3β-hydroxy-lup-20(29)-en-28-oyl)-amino)methyl)-1H-1,2,3-triazolyl)acetyl-4,7,8,9-tetra-O-acetyl-3,5-dideoxy-d-glycero-α-d-galacto-2-nonulopyranosyl)onate* (**28**). Prepared from **9** and **27** according to general procedure A, the residue was purified by chromatography (eluent: PE/Act = 1:1) over silica gel to afford compound **28** as a white solid in 88% yield. *R*_f_ = 0.27 (PE/Act = 1:1); ^1^H-NMR (400 MHz, CDCl_3_): δ 7.71 (s, 1H), 6.55 (d, 1H, *J* = 9.9 Hz), 6.49 (t, 1H, *J* = 5.5 Hz), 5.40 (dt, 1H, *J* = 2.5, 6.2 Hz), 5.30 (dd, 1H, *J* = 2.1, 7.7 Hz), 5.03 (d, 1H, *J* = 16.1 Hz), 4.77–4.83 (m, H), 4.78 (d, 1H, *J* = 16.0 Hz), 4.71 (brs, 1H), 4.56 (brs, 1H), 4.51 (dd, 1H, J = 5.5, 15.0 Hz), 4.40 (dd, 1H, *J* = 5.5, 15.0 Hz), 4.35 (dd, 1H, *J* = 2.5, 12.4 Hz), 4.00–4.17 (m, 3H), 3.73 (s, 3H), 3.29 (s, 3H), 3.14–3.20 (m, 1H), 3.09 (dt, 1H, *J* = 3.9, 11.2 Hz), 2.55 (dd, 1H, *J* = 4.7, 12.8 Hz), 2.39 (dt, 1H, *J* = 3.3, 12.8 Hz), 2.13, 2.10, 2.04, 1.93 (s, 3H each, 4 × CH_3_CO), 0.84–1.95 (m, other aliphatic ring protons), 1.65, 0.94, 0.92, 0.79, 0.77, 0.74 (s, 3H each, 6 × CH_3_), 0.64 (d, 1H, *J* = 8.7 Hz); ^13^C-NMR (100 MHz, CDCl_3_): δ 176.55, 170.83, 170.73, 170.52, 170.31, 168.07, 165.71, 150.97, 145.92, 124.29, 109.50, 99.11, 79.10, 72.31, 68.96, 68.73, 67.49, 62.42, 55.66, 55.45, 52.88, 52.82, 52.54, 50.70, 50.26, 49.66, 46.80, 42.53, 40.86, 38.94, 38.82, 38.31, 37.91, 37.77, 37.30, 34.79, 34.43, 33.59, 30.96, 29.50, 28.12, 27.50, 25.70, 21.24, 21.08, 20.98, 20.92, 19.57, 18.41, 16.30, 15.90, 15.56, 14.74; ESI-HRMS calcd. for C_54_H_82_N_5_O_15_ [M + H]^+^: 1040.5802, found 1040.5809.

*Methyl (O-methyl-5-(N-(4-(N-(3β-hydroxy-lup-20(29)-en-28-oyl)-amino)methyl)-1H-1,2,3-triazolyl)acetyl-3,5-dideoxy-d-glycero-α-d-galacto-2-nonulopyranosyl)onate* (**29**). Prepared from **28** according to general procedure B, the residue was purified by chromatography (eluent: DCM/MeOH = 10:1) over silica gel to afford compound **29** as a white solid in 78% yield. *R*_f_ = 0.26 (DCM/MeOH = 10:1); ^1^H-NMR (400 MHz, CD_3_OD): δ 8.07 (t, 1H, *J* = 5.6 Hz), 7.86 (s, 1H), 5.19 (d, 1H, *J* = 16.0 Hz), 5.15 (d, 1H, *J* = 16.0 Hz), 4.70 (brs, 1H), 4.58 (brs, 1H), 4.41 (dq, 2H, *J* = 5.5, 15.1 Hz), 3.80–3.87 (m, 6H), 3.62–3.73 (m, 3H), 3.53 (d, 1H, *J* = 9.3 Hz), 3.34 (s, 3H), 3.05–3.14 (m, 2H), 2.66 (dd, 1H, *J* = 4.6, 12.8 Hz), 2.53 (dt, 1H, *J* = 3.1, 13.0 Hz), 2.13 (brd, 1H, *J* = 13.4 Hz), 0.92–1.92 (m, other aliphatic ring protons), 1.68, 0.98, 0.95 (s, 3H each, 3 × CH_3_), 0.85 (s, 6H, 2 × CH_3_), 0.76 (s, 3H, CH_3_), 0.69 (d, 1H, *J* = 9.1 Hz); ^13^C-NMR (100 MHz, CD_3_OD): δ 179.15, 170.78, 169.22, 152.29, 146.84, 125.99, 109.97, 100.33, 79.66, 74.51, 72.54, 70.14, 68.64, 64.78, 56.93, 54.03, 53.32, 52.99, 52.09, 52.02, 51.44, 48.08, 43.49, 42.01, 41.36, 40.11, 39.95, 39.19, 38.92, 38.33, 35.55, 34.04, 31.93, 30.54, 28.64, 28.05, 26.99, 22.15, 19.68, 19.50, 16.82, 16.63, 16.17, 15.10; ESI-HRMS calcd. for C_46_H_74_N_5_O_11_ [M + H]^+^: 872.5379, found 872.5385.

*Methyl (O-methyl-9-((4-(N-(3β-hydroxy-lup-20(29)-en-28-oyl)-amino)methyl)-1H-1,2,3-triazolyl)-5-acetamido-3,5-dideoxy-d-glycero-α-d-galacto-2-nonulopyranosyl)onate* (**30**). Prepared from **13** and **27** according to general procedure A, the residue was purified by column chromatography (eluent: DCM/MeOH = 12:1) over silica gel to afford compound **30** as a white solid in 79% yield. *R*_f_ = 0.31 (DCM/MeOH = 12:1); ^1^H-NMR (400 MHz, CD_3_OD): δ 7.82 (s, 1H), 4.82 (m, 1H, overlap with H_2_O), 4.66 (s, 1H), 4.54 (s, 1H), 4.37 (s, 2H), 4.32 (dd, 1H, *J* = 8.6, 14.0 Hz), 4.09 (m, 1H), 3.78 (s, 3H), 3.73 (t, 1H, *J* = 9.8), 3.55–3.63 (m, 2H), 3.38–3.40 (m, 1H), 3.27 (m, 4H), 3.02–2.10 (m, 2H), 2.60 (dd, 1H, *J* = 4.6, 12.2 Hz), 2.45–2.51 (m, 1H), 2.08–2.11 (m, 1H), 1.96 (s, 3H), 0.93–1.85 (m, other aliphatic ring protons), 1.64, 0.95, 0.91 (s, 3H each, 3 × CH_3_), 0.82 (s, 6H, 2 × CH_3_), 0.72 (s, 3H, CH_3_), 0.66 (d, 1H, *J* = 8.5 Hz); ^13^C-NMR (100 MHz, CD_3_OD): δ 179.13, 175.19, 170.71, 152.29, 110.01, 100.40, 79.64, 74.67, 71.65, 71.40, 68.40, 56.94, 56.90, 54.89, 53.82, 53.35, 52.14, 52.09, 51.43, 48.09, 43.49, 42.01, 41.41, 40.11, 39.95, 39.17, 38.97, 38.33, 35.58, 35.53, 33.99, 31.94, 30.56, 28.64, 28.04, 27.00, 22.71, 22.15, 19.66, 19.47, 16.82, 16.69, 16.17, 15.12; ESI-HRMS calcd. for C_46_H_74_O_10_N_5_ [M + H]^+^: 856.5430, found 856.5411.

*Methyl (O-methyl-5-(N-(3β-hydroxy-olean-12-en-28-oyl)-amino)acetyl-4,7,8,9-tetra-O-acetyl-3,5-dideoxy-d-glycero-α-d-galacto-2-nonulopyranosyl)onate* (**34**). Prepared from **10** and **31** according to general procedure C, the residue was purified by column chromatography (eluent: PE/Act = 1:1) over silica gel to afford compound **34** as a white solid in 82% yield. *R*_f_ = 0.23 (PE/Act = 1:1); ^1^H-NMR (400 MHz, CDCl_3_): δ 6.99 (d, 1H, *J* = 9.4 Hz), 6.75 (brs, 1H), 5.44–5.48 (m, 2H), 5.33 (dd, 1H, *J* = 2.2, 9.2 Hz), 4.77 (m, 1H), 4.25 (dd, 1H, *J* = 2.6, 12.4 Hz), 4.18 (dd, 1H, *J* = 2.2, 10.7 Hz), 4.02–4.11 (m, 3H), 3.80 (s, 3H), 3.53 (dd, 1H, *J* = 3.2, 16.2 Hz), 3.31 (s, 3H), 3.22 (t, 1H, *J* = 5.2 Hz), 2.57–2.64 (m, 2H), 2.15, 2.13, 2.04, 2.00 (s, 3H each, 4 × CH_3_CO), 0.96–2.02 (m, other aliphatic ring protons), 1.18, 0.99, 0.93, 0.91, 0.89, 0.79, 0.77 (s, 3H each, 7 × CH_3_); ^13^C-NMR (100 MHz, CDCl_3_): δ 179.04, 170.75, 170.47, 170.04, 169.93, 169.71, 168.14, 144.17, 123.62, 99.03, 79.07, 76.84, 72.37, 69.30, 67.93, 67.40, 62.68, 55.24, 52.77, 52.60, 49.00, 47.72, 46.76, 46.29, 44.01, 42.14, 42.04, 39.48, 38.88, 38.65, 38.15, 37.06, 34.34, 33.09, 32.48, 32.44, 30.83, 28.22, 27.32, 25.96, 24.20, 23.69, 23.54, 21.24, 20.99, 20.91, 18.41, 16.80, 15.73, 15.53; ESI-HRMS calcd. for C_51_H_78_N_2_NaO_15_ [M + Na]^+^: 981.5294, found 981.5294.

*Methyl (O-methyl-5-(N-(3β-hydroxy-olean-12-en-28-oyl)-amino)acetyl-3,5-dideoxy-d-glycero-α-d-galacto-2-nonulopyranosyl)onate* (**35**). Prepared from **34** according to general procedure B, the residue was purified by chromatography (eluent: DCM/MeOH = 10:1) over silica gel to afford compound **35** as a white solid in 85% yield. *R*_f_ = 0.24 (DCM/MeOH = 10:1); ^1^H-NMR (400 MHz, CD_3_OD): δ 7.53 (t, 1H, *J* = 4.8 Hz), 5.38 (brs, 1H), 3.59–3.93 (m, 11H), 3.50 (dd, 1H, *J* = 1.3, 8.7 Hz), 3.35 (s, 3H), 3.15 (dd, 1H, *J* = 4.8, 11.0 Hz), 2.78 (dd, 1H, *J* = 3.4, 9.6 Hz), 2.66 (dd, 1H, *J* = 4.6, 12.8 Hz), 2.10 (dt, 1H, J = 3.3, 12.3 Hz), 1.88–2.01 (m, 2H), 0.99–1.83 (m, other aliphatic ring protons), 1.18, 0.97 (s, 3H each, 2 × CH_3_), 0.95 (s, 6H, 2 × CH_3_), 0.92, 0.78, 0.76 (s, 3H each, 3 × CH_3_), 0.75 (d, 1H, *J* = 11.2 Hz); ^13^C-NMR (100 MHz, CD_3_OD): δ 180.88, 173.08, 170.85, 145.03, 124.42, 100.32, 79.68, 74.74, 72.61, 70.37, 68.57, 65.16, 56.70, 53.85, 53.35, 52.00, 49.00, 47.65, 47.55, 43.83, 42.92, 42.75, 41.32, 40.67, 39.84, 38.12, 35.09, 34.00, 33.82, 33.53, 31.61, 28.75, 28.47, 27.86, 26.45, 24.59, 24.28, 24.04, 19.48, 17.56, 16.33, 16.05; ESI-HRMS calcd. for C_43_H_70_N_2_NaO_11_ [M + Na]^+^: 813.4872, found 813.4876.

*Methyl (O-methyl-5-(N-(3β,16α-dihydroxy-olean-12-en-28-oyl)-amino)acetyl-4,7,8,9-tetra-O-acetyl-3,5-dideoxy-d-glycero-α-d-galacto-2-nonulopyranosyl)onate* (**36**). Prepared from **10** and **32** according to general procedure C, the residue was purified by column chromatography (eluent: PE/Act = 1:1) over silica gel to afford compound **36** as a white solid in 84% yield. *R*_f_ = 0.20 (PE/Act = 1:1); ^1^H-NMR (400 MHz, CDCl_3_): δ 7.84 (brs, 1H), 7.08 (d, 1H, *J* = 5.2 Hz), 5.67 (brs, 1H), 5.46–5.49 (m, 1H), 5.33 (dd, 1H, *J* = 2.3, 8.7 Hz), 4.79 (dt, 1H, *J* = 4.2, 11.2 Hz), 4.30–4.41 (m, 3H), 4.04–4.19 (m, 3H), 3.84 (s, 3H), 3.57 (d, 1H, *J* = 16.7 Hz), 3.31 (s, 3H), 3.22 (brd, 1H, *J* = 6.9 Hz), 2.89 (brs, 1H), 2.66 (brd, 1H, *J* = 13.2 Hz), 2.60 (dd, 1H, *J* = 4.4, 13.0 Hz), 2.32 (t, 1H, *J* = 13.2 Hz), 2.15, 2.14, 2.04, 2.03 (s, 3H each, 4 × CH_3_CO), 0.99–2.02 (m, other aliphatic ring protons), 1.42, 1.00, 0.96, 0.90, 0.87, 0.84, 0.80 (s, 3H each, 7 × CH_3_), 0.76 (d, 1H, *J* = 11.5 Hz); ^13^C-NMR (100 MHz, CDCl_3_): δ 178.19, 170.71, 170.53, 170.07, 170.02, 169.65, 169.16, 143.43, 124.12, 99.27, 79.00, 76.06, 73.03, 69.37, 68.32, 67.54, 62.92, 55.26, 53.03, 52.70, 48.61, 48.53, 47.23, 46.78, 44.06, 41.80, 41.58, 39.58, 38.87, 38.76, 38.21, 37.02, 35.98, 34.60, 32.67, 30.94, 30.54, 28.21, 27.29, 26.61, 24.11, 23.74, 21.22, 21.17, 20.93, 20.89, 18.37, 16.80, 15.79, 15.75; ESI-HRMS calcd. for C_51_H_78_N_2_NaO_16_ [M + Na]^+^: 997.5244, found 997.5252.

*Methyl (O-methyl-5-(N-(3β,16α-dihydroxy-olean-12-en-28-oyl)-amino)acetyl-3,5-dideoxy-d-glycero-α-d-galacto-2-nonulopyranosyl)onate* (**37**). Prepared from **36** according to general procedure B, the residue was purified by chromatography (eluent: DCM/MeOH = 10:1) over silica gel to afford compound **37** as a white solid in 83% yield. *R*_f_ = 0.21 (DCM/MeOH = 10:1); ^1^H-NMR (400 MHz, CD_3_OD): δ 7.51 (t, 1H, *J* = 4.7 Hz), 5.51 (brs, 1H), 4.28 (brs, 1H), 3.58–3.95 (m, 11H), 3.46 (dd, 1H, *J* = 1.3, 8.8 Hz), 3.34 (s, 3H), 3.16 (dd, 1H, *J* = 4.9, 11.1 Hz), 2.89 (dd, 1H, *J* = 3.4, 13.7 Hz), 2.66 (dd, 1H, *J* = 4.6, 12.8 Hz), 2.31 (t, 1H, *J* = 13.2 Hz), 1.81–1.97 (m, 4H), 1.00–1.75 (m, other aliphatic ring protons), 1.38, 0.98, 0.97, 0.96, 0.90, 0.80, 0.79 (s, 3H each, 7 × CH_3_), 0.77 (d, 1H, *J* = 12.6 Hz); ^13^C-NMR (100 MHz, CD_3_OD): δ 180.62, 172.89, 170.82, 144.61, 124.56, 100.30, 79.66, 75.58, 74.73, 72.59, 70.44, 68.57, 65.18, 56.81, 53.80, 53.36, 52.00, 50.29, 48.26, 48.01, 43.92, 42.93, 42.63, 41.28, 40.91, 39.99, 39.84, 38.08, 36.17, 36.06, 33.97, 33.26, 31.12, 30.83, 28.73, 27.90, 27.45, 25.75, 24.55, 19.46, 17.61, 16.33; ESI-HRMS calcd. for C_43_H_70_N_2_NaO_12_ [M + Na]^+^: 829.4821, found 829.4826.

*Methyl (O-methyl-5-(N-(3β-hydroxy-urs-12-en-28-oyl)-amino)acetyl-4,7,8,9-tetra-O-acetyl-3,5-dideoxy-d-glycero-α-d-galacto-2-nonulopyranosyl)onate* (**38**). Prepared from **10** and **33** according to general procedure C, the residue was purified by column chromatography (eluent: PE/Act = 1:1) over silica gel to afford compound **38** as a white solid in 80% yield. *R*_f_ = 0.24 (PE/Act = 1:1); ^1^H-NMR (400 MHz, CDCl_3_): δ 6.84 (d, 1H, *J* = 9.0 Hz), 6.71 (brs, 1H). 5.44–5.48 (m, 1H), 5.40 (brs, 1H), 5.32 (dd, 1H, *J* = 1.6, 9.3 Hz), 4.75 (m, 1H), 4.24 (dd, 1H, *J* = 2.5, 12.4 Hz), 4.06–4.17 (m, 3H), 3.98 (dd, 1H, *J* = 6.1, 16.3 Hz), 3.81 (s, 3H), 3.56 (dd, 1H, *J* = 3.2, 16.3 Hz), 3.31 (s, 3H), 3.18–3.23 (m, 1H), 2.63 (dd, 1H, *J* = 4.5, 12.7 Hz), 2.15, 2.13, 2.03, 1.99 (s, 3H each, 4 × CH_3_CO), 1.03–2.06 (m, other aliphatic ring protons), 1.11, 0.99, 0.96, 0.94 (s, 3H each, 4 × CH_3_), 0.88 (d, 3H, *J* = 6.4 Hz, CH_3_), 0.79, 0.76 (s, 3H each, 2 × CH_3_), 0.73 (d, 1H, *J* = 11.5 Hz); ^13^C-NMR (100 MHz, CDCl_3_): δ 178.80, 170.73, 170.49, 170.03, 169.97, 169.85, 168.12, 138.99, 126.50, 99.01, 79.11, 72.37, 69.30, 67.95, 67.32, 62.63, 55.26, 53.62, 52.79, 52.60, 48.93, 47.73 (2C), 43.87, 42.44, 39.84, 39.66, 39.13, 38.87, 38.79, 38.17, 37.19, 37.05, 32.87, 31.06, 28.24, 27.86, 27.31, 25.18, 23.53, 21.34, 21.23, 21.03, 21.00, 20.90, 18.40, 17.28, 16.78, 15.77, 15.60; ESI-HRMS calcd. for C_51_H_79_N_2_O_15_ [M + H]^+^: 959.5475, found 959.5472.

*Methyl (O-methyl-5-(N-(3β-hydroxy-urs-12-en-28-oyl)-amino)acetyl-3,5-dideoxy-d-glycero-α-d-galacto-2-nonulopyranosyl)onate* (**39**). Prepared from **38** according to general procedure B, the residue was purified by chromatography (eluent: DCM/MeOH = 10:1) over silica gel to afford compound **37** as a white solid in 86% yield. *R*_f_ = 0.25 (DCM/MeOH = 10:1); ^1^H-NMR (400 MHz, CD_3_OD): δ 5.37 (brs, 1H), 3.85–3.91 (m, 6H), 3.59–3.80 (m, 5H), 3.49 (d, 1H, *J* = 8.8 Hz), 3.34 (s, 3H), 3.16 (dd, 1H, *J* = 4.8, 11.0 Hz), 2.66 (dd, 1H, *J* = 4.7, 12.8 Hz), 2.12 (d, 1H, *J* = 11.0 Hz), 2.08 (dd, 2H, *J* = 4.1, 13.5 Hz), 1.95–1.98 (m, 2H), 0.98–1.76 (m, other aliphatic ring protons), 1.13 (s, 3H, CH_3_), 0.98 (s, 6H, 2 × CH_3_), 0.97 (s, 3H, CH_3_), 0.92 (d, 3H, *J* = 6.4 Hz, CH_3_), 0.78 (2 × s, 6H, 2 × CH_3_), 0.75 (d, 1H, *J* = 10.7 Hz); ^13^C-NMR (100 MHz, CD_3_OD): δ 180.66, 173.00, 170.83, 139.65, 127.62, 100.31, 79.65, 74.71, 72.62, 70.37, 68.54, 65.14, 56.67, 54.28, 53.84, 53.35, 52.00, 49.00, 48.90, 43.77, 43.29, 41.31, 40.85, 40.81, 40.30, 39.99, 39.82, 38.38, 38.06, 34.12, 31.89, 28.93, 28.78, 27.88, 25.45, 24.43, 24.08, 21.6, 19.45, 17.75, 17.58, 16.39, 16.21; ESI-HRMS calcd. for C_43_H_70_N_2_NaO_11_ [M + Na]^+^: 813.4872, found 813.4879.

*Methyl (O-methyl-9-(N-(3β-hydroxy-olean-12-en-28-oyl)amino)-5-acetamido-3,5-dideoxy-d-glycero-α-d-galacto-2-nonulopyranosyl)onate* (**40**). Prepared from **14** and **31** according to general procedure C, the residue was purified by column chromatography (eluent: DCM/MeOH = 12:1) over silica gel to afford compound **40** as a white solid in 80% yield. *R*_f_ = 0.27 (DCM/MeOH = 12:1); ^1^H-NMR (400 MHz, CD_3_OD): δ 7.15 (t, 1H, *J* = 5.0 Hz), 5.39 (brs, 1H), 3.88 (dt, 1H, *J* = 3.2, 8.1 Hz), 3.84 (s, 3H), 3.73–3.78 (m, 2H), 3.60–3.67 (m, 2H), 3.36 (s, 1H), 3.34 (s, 3H), 3.06–3.17 (m, 2H), 2.74 (d, 1H, *J* = 9.7 Hz), 2.63 (dd, 1H, *J* = 4.4, 12.8 Hz), 0.98–2.09 (m, other aliphatic ring protons), 1.99 (s, 3H, CH_3_CO), 1.19, 0.97, 0.95, 0.94, 0.92, 0.82, 0.78 (s, 3H each, 7 × CH_3_), 0.76 (m, 1H); ^13^C-NMR (100 MHz, CD_3_OD): δ 180.91, 175.00, 170.78, 145.1, 124.54, 100.37, 79.65, 74.67, 72.4, 70.65, 68.53, 56.66, 53.81, 53.28, 52.09, 47.73, 47.67, 44.66, 43.00, 42.96, 41.35, 40.71, 39.83, 38.11, 35.14, 34.00, 33.76, 33.53, 31.62, 28.75, 28.49, 27.86, 26.45, 24.62, 24.37, 24.09, 22.76, 19.47, 17.86, 16.31, 15.99; ESI-HRMS calcd. for C_43_H_71_N_2_O_10_ [M + H]^+^: 775.5103, found 775.5089.

*Methyl (O-methyl-9-(N-(3β,16α-dihydroxy-olean-12-en-28-oyl)amino)-5-acetamido-3,5-dideoxy-d-glycero-α-d-galacto-2-nonulopyranosyl)onate* (**41**). Prepared from **14** and **32** according to general procedure C, the residue was purified by column chromatography (eluent: DCM/MeOH = 12:1) over silica gel to afford compound **41** as a white solid in 75% yield. *R*_f_ = 0.24 (DCM/MeOH = 12:1); ^1^H-NMR (400 MHz, CD_3_OD): δ 7.17 (brs, 1H), 5.51 (brs, 1H), 4.33 (brs, 1H), 3.85 (m, 1H), 3.84 (s, 3H), 3.73–3.78 (m, 2H), 3.58–3.67 (m, 2H), 3.36 (s, 1H), 3.33 (s, 3H), 3.16 (dd, 1H, *J* = 4.8, 11.0 Hz), 3.00–3.06 (m, 1H), 2.81 (dd, 1H, *J* = 2.7, 10.6 Hz), 2.64 (dd, 1H, *J* = 4.5, 12.8 Hz), 2.37 (t, 1H, *J* = 13.2 Hz), 1.02–2.01 (m, other aliphatic ring protons), 2.00 (s, 3H, CH_3_CO), 1.39, 0.98 (s, 3H each, 2 × CH_3_), 0.96 (2 × s, 6H, 2 × CH_3_), 0.89, 0.83, 0.78 (s, 3H each, 3 × CH_3_), 0.77 (m, 1H); ^13^C-NMR (100 MHz, CD_3_OD): δ 180.70, 175.08, 170.81, 144.95, 124.61, 100.35, 79.64, 75.76, 74.72, 72.36, 70.65, 68.52, 56.77, 53.81, 53.33, 52.07, 50.28, 48.21, 48.16, 44.51, 42.90, 42.88, 41.35, 40.87, 39.99, 39.84, 38.09, 36.39, 36.04, 33.92, 33.23, 31.59, 31.22, 28.72, 27.90, 27.30, 25.44, 24.58, 22.77, 19.44, 17.87, 16.32, 16.22; ESI-HRMS calcd. for C_43_H_71_N_2_O_11_ [M + H]^+^: 791.5052, found 791.5032.

*Methyl (O-methyl-9-(N-(3β-hydroxy-urs-12-en-28-oyl)amino)-5-acetamido-3,5-dideoxy-d-glycero-α-d-galacto-2-nonulopyranosyl)onate* (**42**). Prepared from **14** and **33** according to general procedure C, the residue was purified by column chromatography (eluent: DCM/MeOH = 12:1) over silica gel to afford compound **42** as a white solid in 83% yield. *R*_f_ = 0.28 (DCM/MeOH = 12:1); ^1^H-NMR (400 MHz, CDCl_3_): δ 7.24 (brs, 1H), 6.61 (brs, 1H), 5.34 (s, 1H), 4.99 (s, 1H), 4.54 (brs, 1H), 3.95 (s, 1H), 3.85 (s, 4H), 3.71 (s, 3H), 3.51 (d, 1H, *J* = 7.9 Hz), 3.32 (s, 4H), 3.20 (m, 1H), 3.14 (brs, 1H), 2.71 (d, 1H, *J* = 12.2 Hz), 2.03 (s, 3H, CH_3_CO), 1.04–1.99 (m, other aliphatic ring protons), 1.10, 0.99, 0.95, 0.89, 0.87, 0.79, 0.77 (s, 3H each, 7 × CH_3_), 0.72 (d, 1H, *J* = 11.2 Hz); ^13^C-NMR (100 MHz, CDCl_3_): δ 179.46, 173.63, 169.66, 138.91, 126.19, 99.05, 78.91, 73.48, 70.91, 70.36, 67.41, 55.10, 53.60, 53.48, 53.38, 53.12, 51.55, 47.79, 47.54, 43.5, 42.39, 39.73, 39.55, 38.97, 38.73, 38.64, 37.29, 36.90, 32.79, 30.87, 28.13, 27.78, 27.18, 24.91, 23.37, 23.27, 23.04, 21.18, 18.26, 17.15, 16.87, 15.64, 15.44; ESI-HRMS calcd. for C_43_H_71_N_2_O_10_ [M + H]^+^: 775.5103, found 775.5090.

*Methyl (O-methyl-5-(N-(3β-hydroxy-lup-20(29)-en-28-oyl)-amino)acetyl-4,7,8,9-tetra-O-acetyl-3,5-dideoxy-d-glycero-α-d-galacto-2-nonulopyranosyl)onate* (**44**). Prepared from **10** and **43** according to general procedure C, the residue was purified by column chromatography (eluent: PE/Act = 1:1) over silica gel to afford compound **44** as a white solid in 75% yield. *R*_f_ = 0.25 (PE/Act = 1:1); ^1^H-NMR (400 MHz, CDCl_3_): δ 6.41 (t, 1H, *J* = 5.3 Hz), 6.24 (d, 1H, *J* = 9.5 Hz), 5.43–5.47 (m, 1H), 5.29 (t, 1H, *J* = 7.3 Hz), 4.78–4.85 (m, 1H), 4.76 (brs 1H), 4.61 (brs, 1H), 4.23 (dd, 1H, *J* = 2.6, 12.4 Hz), 4.05–4.10 (m, 3H), 3.91 (dd, 1H, *J* = 5.3, 15.6 Hz), 3.83 (s, 3H), 3.66 (dd, 1H, *J* = 5.5, 15.6 Hz), 3.31 (s, 3H), 3.12–3.19 (m, 2H), 2.63 (dd, 1H, *J* = 4.6, 12.7 Hz), 2.38 (dt, 1H, *J* = 3.0, 11.6 Hz), 2.13 (s, 6H, 2 × CH_3_CO), 2.03, 2.02 (s, 3H each, 2 × CH_3_CO), 0.87–2.06 (m, other aliphatic ring protons), 1.70, 0.97, 0.96, 0.90, 0.81, 0.75 (s, 3H each, 6 × CH_3_), 0.68 (d, 1H, *J* = 8.8 Hz); ^13^C-NMR (100 MHz, CDCl_3_): δ 177.50, 170.74, 170.68, 170.46, 170.38, 170.05, 168.05, 150.81, 109.65, 98.99, 79.07, 72.25, 69.30, 67.79, 67.39, 62.48, 55.98, 55.47, 52.89, 52.61, 50.69, 50.26, 49.06, 46.73, 43.85, 42.58, 40.84, 38.97, 38.83, 38.27, 38.08, 37.90, 37.31, 34.45, 33.81, 31.27, 29.59, 28.12, 27.50, 25.70, 21.21, 21.06, 20.97, 20.91, 19.58, 18.42, 16.24, 16.22, 15.52, 14.77; ESI-HRMS calcd. for C_51_H_79_N_2_O_15_ [M + H]^+^: 959.5475, found 959.5481.

*Methyl (O-methyl-5-(N-(3β-hydroxy-lup-20(29)-en-28-oyl)-amino)acetyl-3,5-dideoxy-d-glycero-α-d-galacto-2-nonulopyranosyl)onate* (**45**). Prepared from **44** according to general procedure B, the residue was purified by chromatography (eluent: DCM/MeOH = 10:1) over silica gel to afford compound **45** as a white solid in 78% yield. *R*_f_ = 0.25 (DCM/MeOH = 10:1); ^1^H-NMR (400 MHz, CD_3_OD): δ 7.86 (t, 1H, *J* = 5.6 Hz), 4.73 (s, 1H), 4.59 (s, 1H), 3.61–3.88 (m, 10H), 3.58–3.59 (m, 1H), 3.56 (s, 1H), 3.35 (s, 3H), 3.05–3.15 (m, 2H), 2.68 (dd, 1H, *J* = 4.6, 12.8 Hz), 2.49 (dt, 1H, *J* = 3.2, 12.7 Hz), 2.16–2.18 (m, 1H, overlap with acetone), 1.86–1.98 (m, 2H), 0.91–1.78 (m, other aliphatic ring protons), 1.70, 1.00 (s, 3H each, 2 × CH_3_), 0.95 (s, 6H, 2 × CH_3_), 0.86, 0.75 (s, 3H each, 2 × CH_3_), 0.71 (d, 1H, *J* = 8.6 Hz); ^13^C-NMR (100 MHz, CD_3_OD): δ 179.96, 174.01, 170.89, 152.30, 110.01, 100.29, 79.65, 74.94, 72.73, 70.40, 68.52, 65.38, 57.05, 56.89, 53.85, 53.40, 52.08, 51.99, 51.38, 48.06, 43.65, 43.54, 42.00, 41.37, 40.11, 39.94, 39.28, 38.99, 38.33, 35.61, 34.06, 32.03, 30.66, 28.64, 28.04, 27.01, 22.15, 19.82, 19.47, 16.82, 16.80, 16.13, 15.11; ESI-HRMS calcd. for C_43_H_70_N_2_NaO_11_ [M + Na]^+^: 813.4872, found 813.4878.

*Methyl (O-methyl-9-(N-(3β-hydroxy-lup-20(29)-en-28-oyl)amino)-5-acetamido-3,5-dideoxy-d-glycero-α-d-galacto-2-nonulopyranosyl)onate* (**46**). Prepared from **14** and **43** according to general procedure C, the residue was purified by column chromatography (eluent: DCM/MeOH = 12:1) over silica gel to afford compound **46** as a white solid in 77% yield. *R*_f_ = 0.29 (DCM/MeOH = 12:1); ^1^H-NMR (400 MHz, CD_3_OD): δ 7.37 (t, 1H, *J* = 5.4 Hz), 4.72 (s, 1H), 4.59 (s, 1H), 3.90–3.95 (m, 1H), 3.84 (s, 3H), 3.78 (t, 1H, *J* = 10.2 Hz), 3.61–3.67 (m, 2H), 3.56 (dt, 1H, *J* = 4.3, 13.9 Hz), 3.41 (t, 1H, *J* = 5.9 Hz), 3.33–3.37 (m, 4H), 3.06–3.14 (m, 2H), 2.64 (dd, 1H, *J* = 4.5, 12.7 Hz), 2.54 (dt, 1H, *J* = 2.6, 11.1 Hz), 2.10–2.15 (m, 1H), 1.98 (s, 3H, CH_3_CO), 1.69, 1.00 (s, 3H each, 2 × CH_3_), 0.94–1.92 (m, other aliphatic ring protons), 0.95 (s, 6H, 2 × CH_3_), 0.85, 0.75 (s, 3H each, 2 × CH_3_), 0.70 (d, 1H, *J* = 9.8 Hz); ^13^C-NMR (100 MHz, CD_3_OD): δ 179.67, 174.82, 170.82, 152.24, 110.12, 100.31, 79.64, 74.56, 71.88, 70.99, 68.63, 57.15, 56.89, 53.84, 53.30, 52.08, 52.04, 51.42, 48.16, 43.84, 43.54, 41.99, 41.39, 40.11, 39.94, 39.43, 38.99, 38.33, 35.60, 34.29, 32.05, 30.60, 28.64, 28.04, 26.96, 22.89, 22.13, 19.66, 19.45, 16.90, 16.78, 16.13, 15.14; ESI-HRMS calcd. for C_43_H_71_N_2_O_10_ [M + H]^+^: 775.5103, found 775.5089.

### 3.2. Bioassays

#### 3.2.1. Cytotoxicity Assay

The assay was performed as previously described with some modifications [39]. Cells were seeded in 96-well plates in DMEM supplemented with 10% FBS and cultured overnight at 37 °C in 5% CO_2_. Then, the test compounds were added and the cells were further incubated at 37 °C in 5% CO_2_ for 40 h. Cell viability was assessed using the CellTiter-Glo assay kit (as recommended by the supplier, Promega Corp., Madison, WI, USA) and the plates were read using a plate reader (Tecan Infinite M2000 PRO; Tecan Group Ltd., Mannedorf, Switzerland). Viability was calculated using the background-corrected absorbance as follows:

Viability (%) = A of experiment well/A of control well × 100%

#### 3.2.2. MTT Assay

The compounds were dissolved in DMSO and diluted to the required concentration with the culture medium when used. The cells harvested from the exponential phase were equivalently seeded into a 96-well plate and compounds were then added to the wells to achieve final concentrations ranging from 10^−7^ to 10^−5^ M. Control wells were prepared by the addition of the culture medium. Wells containing the culture medium without cells were used as blanks. The plates were incubated at 37 °C in a 5% CO_2_ incubator for 44 h. The MTT assay was performed (as described by Mosmann [35]). Upon completion of the incubation, a stock MTT dye solution (20 μL, 5 mg/mL) was added to each well. After 4 h of incubation, 2-propanol (100 μL) was added to solubilize the MTT formazan. The OD of each well was then determined on a microplate spectrophotometer, at a wavelength of 570 nm.

#### 3.2.3. CPE Reduction Assay

MDCK cells were seeded into 96-well plates in DMEM, supplemented with 10% FBS and incubated overnight at 37 °C under 5% CO_2_. The culture medium was replaced by the test compound and the influenza virus (MOI = 0.1)-DMEM, supplemented with 1% FBS and 2 μg/mL TPCK-treated trypsin. The final concentration of DMSO was 1%. After 40 h of incubation, CellTiter-Glo reagent (Promega Corp., Madison, WI, USA) was added and the CellTiter-Glo assay was performed.

## 4. Conclusions

We synthesized and characterized a series of novel sialic acid (C-5 or C-9)-pentacyclic triterpene conjugates, and their cytotoxicity and anti-influenza A/WSN/33 virus activity were evaluated. Two sialic acid (C-9)-ursolic acid conjugates **26** and **42** showed strong cytotoxicity to MDCK cells at a concentration of 100 μM. Most compounds had no significant activity against the influenza A/WSN/33 strain, except that four compounds **20**, **28**, **36**, and **44** showed weak anti-influenza virus activity. Compound **20** exhibited a synergistic effect (when combined OSV) in inhibiting influenza infection. These results indicated that the positions of C-5 and C-9 of sialic acid were important for its binding with the HA protein during virus entry into host cells, while C-4 and C-2 hydroxy were not critical for the binding process and could be replaced with hydrophobic moieties. Therefore, the synergistic effect of pentacyclic triterpene with other anti-influenza virus inhibitors may provide a new option for the treatment of the influenza virus infection.

## Supplementary Materials

Supplementary materials are available online. Table S1: The cytotoxicity of compounds **26** and **42** against HL-60, Hela and A549 cell lines. Selected ^1^H-, ^13^C-NMR, and HRMS spectra.
