# Supplementary material for: Synthesis and In Vitro Anti-Influenza Virus Evaluation of Novel Sialic Acid (C-5 and C-9)-Pentacyclic Triterpene Derivatives
Source: Molecules. 2017 Jun 22;22(7):1018. doi: 10.3390/molecules22071018 (PMC6152041; doi:10.3390/molecules22071018)
Supplement: Supplementary file 1 [file molecules-22-01018-s001.pdf]

Supporting Information

Synthesis and In Vitro Anti-influenza Virus Evaluation of Novel Sialic Acid (C-5 and C-9)-Pentacyclic Triterpene Derivatives

Xu Han [1,†], Long-Long Si [1,†], Yong-Ying Shi [1], Zi-Bo Fan [1], Shou-Xin Wang [1,3], Zhen-Yu Tian [1], Man Li [1], Jia-Qi Sun [1], Ping-Xuan Jiao [1], Fu-Xiang Ran [1], Yong-Min Zhang [2], De-Min Zhou [1], Su-Long Xiao [1,*]

1. State Key Laboratory of Natural and Biomimetic Drugs, School of Pharmaceutical Sciences, Peking University, Beijing 100191, China; E-Mails: ax_han@126.com (X. H.); silonglong@bjmu.edu.cn (L.-L S.); sygne24501@sina.com (Y.-Y. S); fancyfzb@163.com (Z.-B. F.); shouxinwang@126.com (S.-X. W.); fashankc@163.com (Z.-Y.T.), 18811711762@163.com (M.L.), jiaqi.sun@pku.edu.cn (J.-Q.S.); 15022326072@163.com (P.-X.J.); rfx@bjmu.edu.cn (F.-X. R); Deminzhou@bjmu.edu.cn (D.-M. Z)
2. Institut Parisien de Chimie Moléculaire, CNRS UMR 8232, Université Pierre & Marie Curie-Paris 6, 4 Place Jussieu, 75005 Paris, France; yongmin.zhang@upmc.fr (Y.-M. Z)
3. School of Pharmacy, Jining Medical University, Rizhao 276826, China

1. **Table S1**. The cytotoxicity of compounds **26** and **42** against HL-60, Hela and A549 cell lines. Selected ^{1}H, ^{13}C NMR and HRMS spectra.

NMR or HRMS	Page
^{1}H NMR of compound **9**	S4
^{13}C NMR of compound **9**	S4
HRMS of compound **9**	S5
^{1}H NMR of compound **18**	S5
^{13}C NMR of compound **18**	S6
HRMS of compound **18**	S6
^{1}H NMR of compound **19**	S7
^{13}C NMR of compound **19**	S7
HRMS of compound **19**	S8
^{1}H NMR of compound **20**	S8
^{13}C NMR of compound **20**	S9
HRMS of compound **20**	S9
^{1}H NMR of compound **21**	S10
^{13}C NMR of compound **21**	S10

HRMS of compound **21**	S11
^{1}H NMR of compound **22**	S11
^{13}C NMR of compound **22**	S12
HRMS of compound **22**	S12
^{1}H NMR of compound **23**	S13
^{13}C NMR of compound **23**	S13
HRMS of compound **23**	S14
^{1}H NMR of compound **24**	S14
^{13}C NMR of compound **24**	S15
HRMS of compound **24**	S15
^{1}H NMR of compound **25**	S16
^{13}C NMR of compound **25**	S16
HRMS of compound **25**	S17
^{1}H NMR of compound **26**	S17
^{13}C NMR of compound **26**	S18

HRMS of compound **26**	S18
^{1}H NMR of compound **28**	S19
^{13}C NMR of compound **28**	S19
HRMS of compound **28**	S20
^{1}H NMR of compound **29**	S20
^{13}C NMR of compound **29**	S21
HRMS of compound **29**	S21
^{1}H NMR of compound **30**	S22
^{13}C NMR of compound **30**	S22
HRMS of compound **30**	S23
^{1}H NMR of compound **34**	S23
^{13}C NMR of compound **34**	S24
HRMS of compound **34**	S24
^{1}H NMR of compound **35**	S25
^{13}C NMR of compound **35**	S25
HRMS of compound **35**	S26
^{1}H NMR of compound **36**	S26
^{13}C NMR of compound **36**	S27
HRMS of compound **36**	S27
^{1}H NMR of compound **37**	S28
^{13}C NMR of compound **37**	S28
HRMS of compound **37**	S29
^{1}H NMR of compound **38**	S29

^{13}C NMR of compound **38**	S30
HRMS of compound **38**	S30
^{1}H NMR of compound **39**	S31
^{13}C NMR of compound **39**	S32
HRMS of compound **39**	S32
^{1}H NMR of compound **40**	S33
^{13}C NMR of compound **40**	S33
HRMS of compound **40**	S34
^{1}H NMR of compound **41**	S34
^{13}C NMR of compound **41**	S35
HRMS of compound **41**	S35
^{1}H NMR of compound **42**	S36
^{13}C NMR of compound **42**	S36
HRMS of compound **42**	S37
^{1}H NMR of compound **44**	S37
^{13}C NMR of compound **44**	S38
HRMS of compound **44**	S38
^{1}H NMR of compound **45**	S39
^{13}C NMR of compound **45**	S39
HRMS of compound **45**	S40
^{1}H NMR of compound **46**	S40
^{13}C NMR of compound **46**	S41
HRMS of compound **46**	S41

1. **Table S1**. The cytotoxicity of compounds **26** and **42** against HL-60, Hela and A549 cell lines.

Compounds	Cell lines	0.1 μM	1 μM	10 μM
**26**	HL-60	3.52	4.01	2.33
	Hela	-3.34	6.6	9.46
	A549	2.16	5.23	0.21
**42**	HL-60	-2	13.6	53
	Hela	1.18	20.13	6.04
	A549	2.33	-0.37	17.7
5-Fu	HL-60	85.22[a]		
	Hela	90.32[a]		
	A549	77.20[a]		

[a] Measured in a dose of 0.5 μM.

1. ^{1}H NMR (400 MHz, $CDCl_3$) spectrum of compound **9**

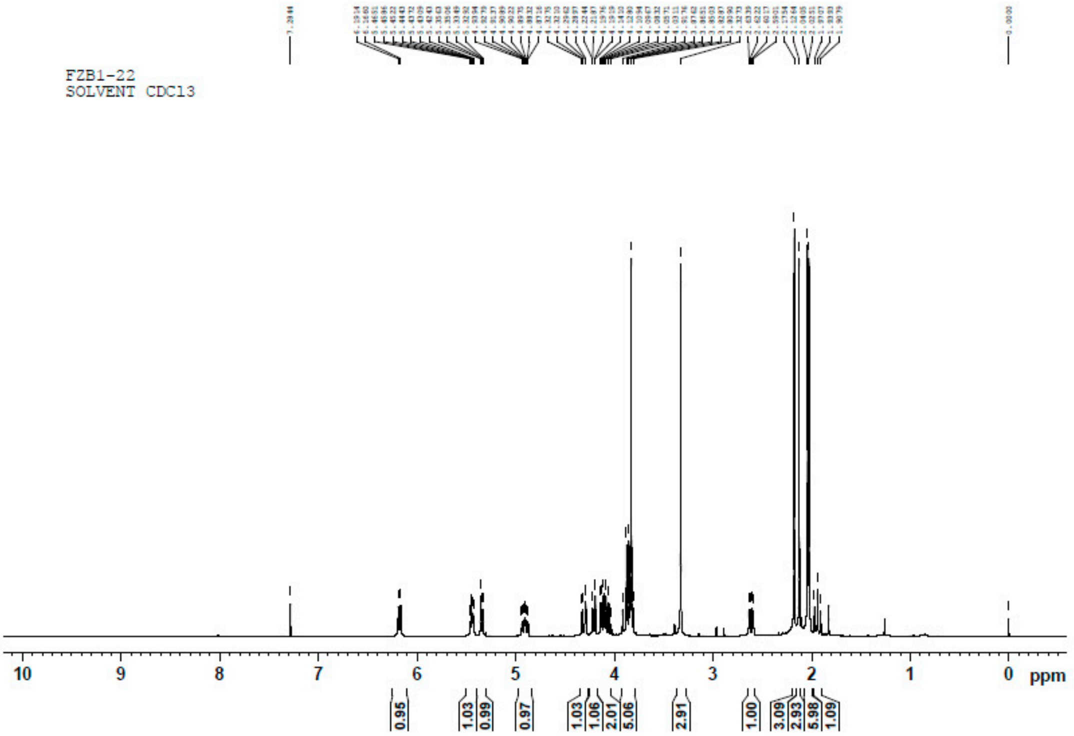

2. ^{13}C NMR (100 MHz, $CDCl_3$) spectrum of compound **9**

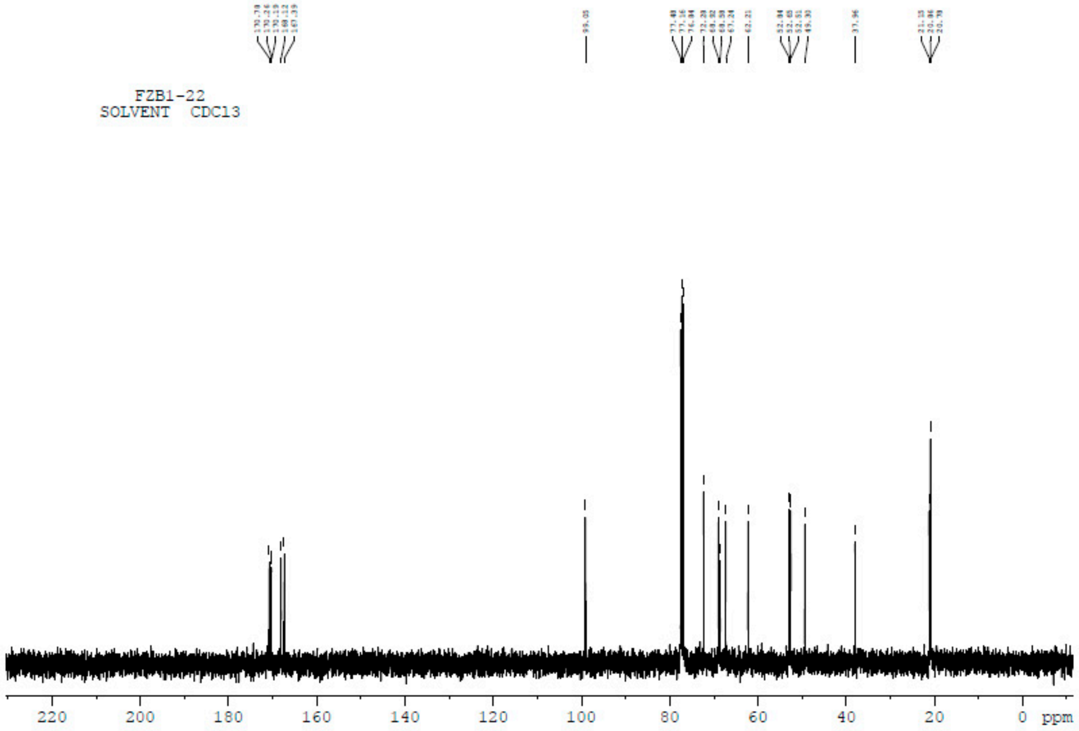

3. HR-ESI-MS spectrum of compound **9**

HIGH RESLUTION MASS SPECTROMETRY REPORT

Sample No.	Formula (M)	Ion Formula	Measured m/z	Calc m/z	Diff (ppm)
FZB1-22	C21 H30 N4 O13	C21 H30 N4 Na O13	569.1708	569.1702	-1.21

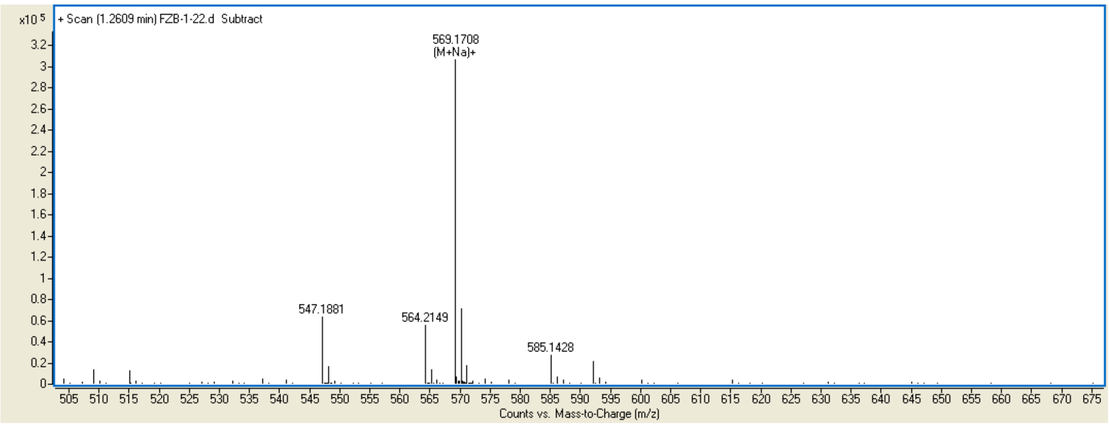

4. ^{1}H NMR (400 MHz, $CDCl_3$) spectrum of **18**

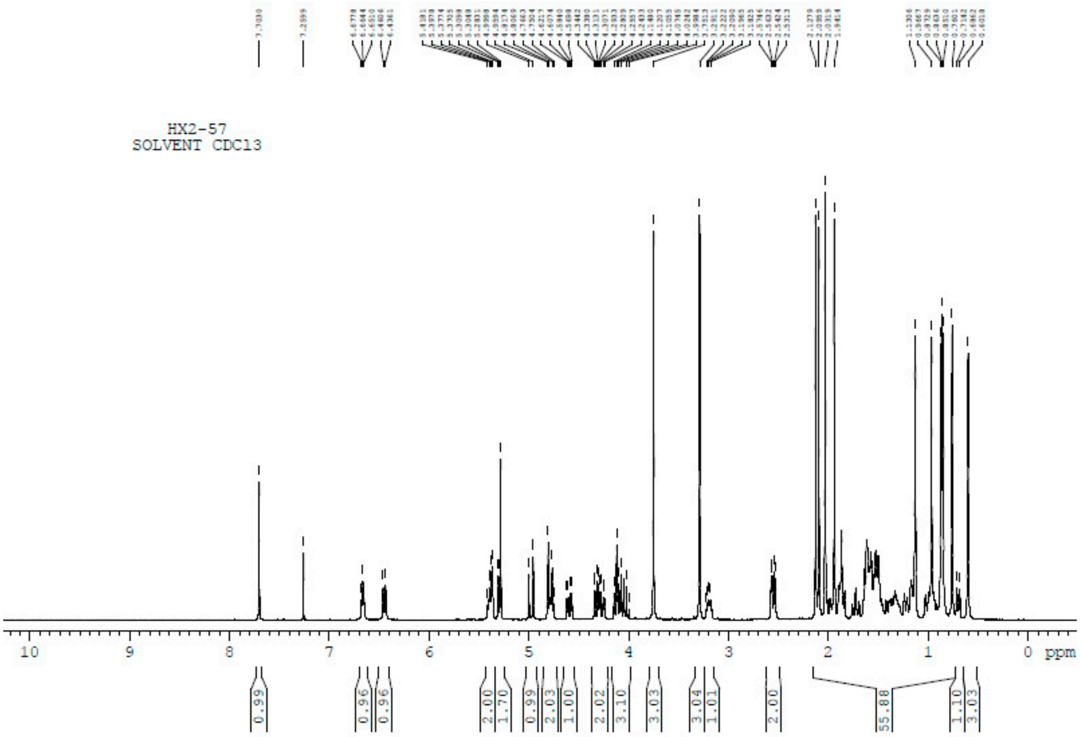

5. ^{13}C NMR (100 MHz, $CDCl_3$) spectrum of compound **18**

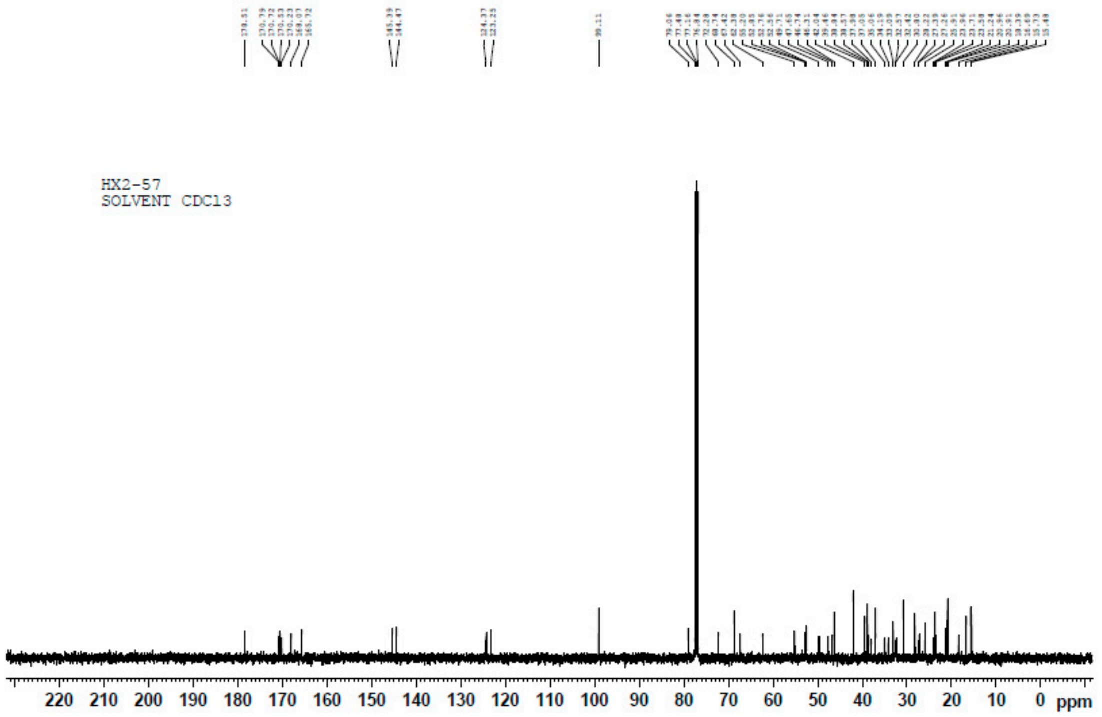

6. HR-ESI-MS spectrum of compound **18**

HIGH RESLUTION MASS SPECTROMETRY REPORT

Sample No.	Formula (M)	Ion Formula	Measured m/z	Calc m/z	Diff (ppm)
HX2-57	C54 H81 N5 O15	C54 H82 N5 O15	1040.5798	1040.5802	0.37

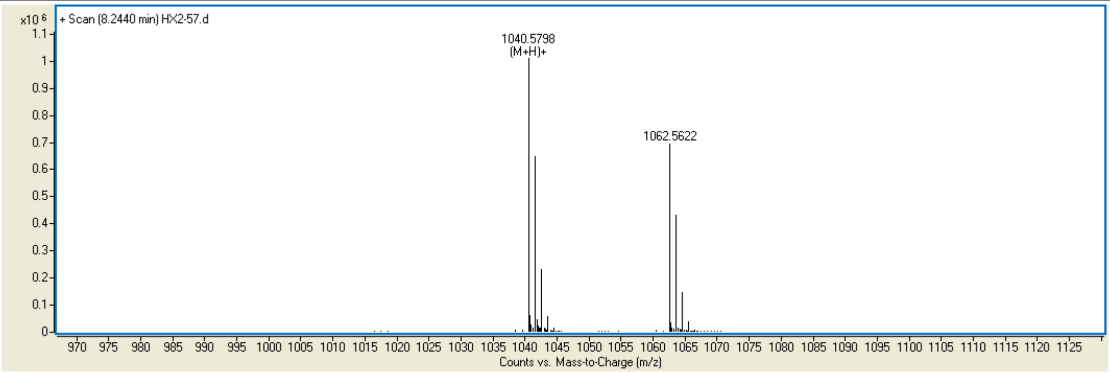

7. ^{1}H NMR (400 MHz, CD_3OD) spectrum of compound **19**

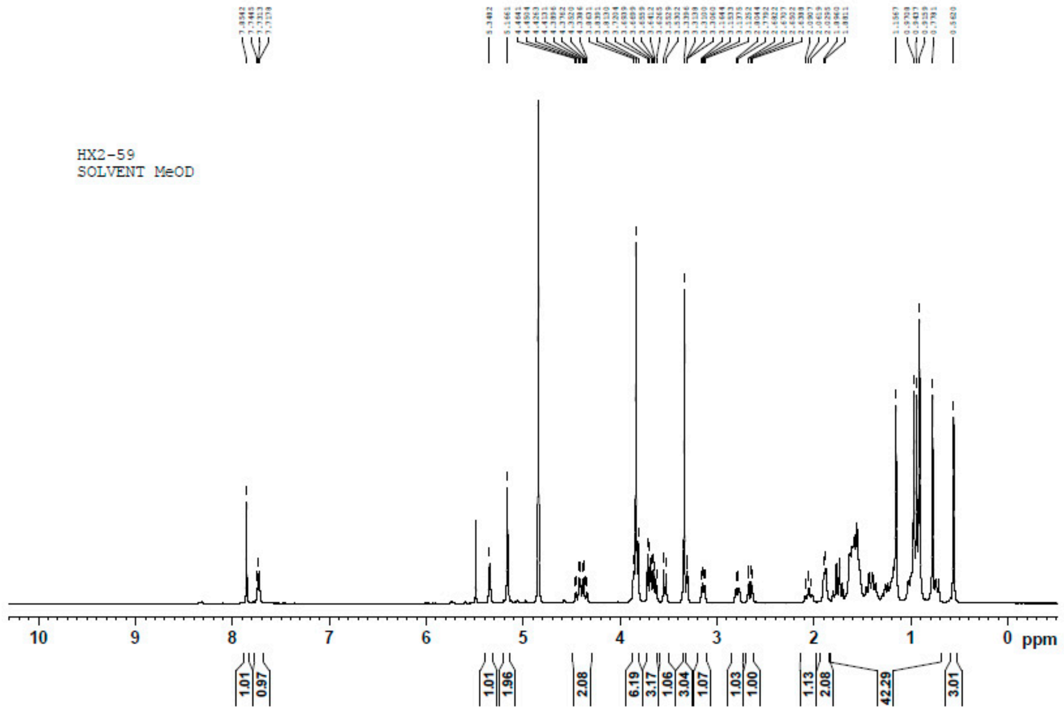

8. ^{13}C NMR (100 MHz, CD_3OD) spectrum of compound **19**

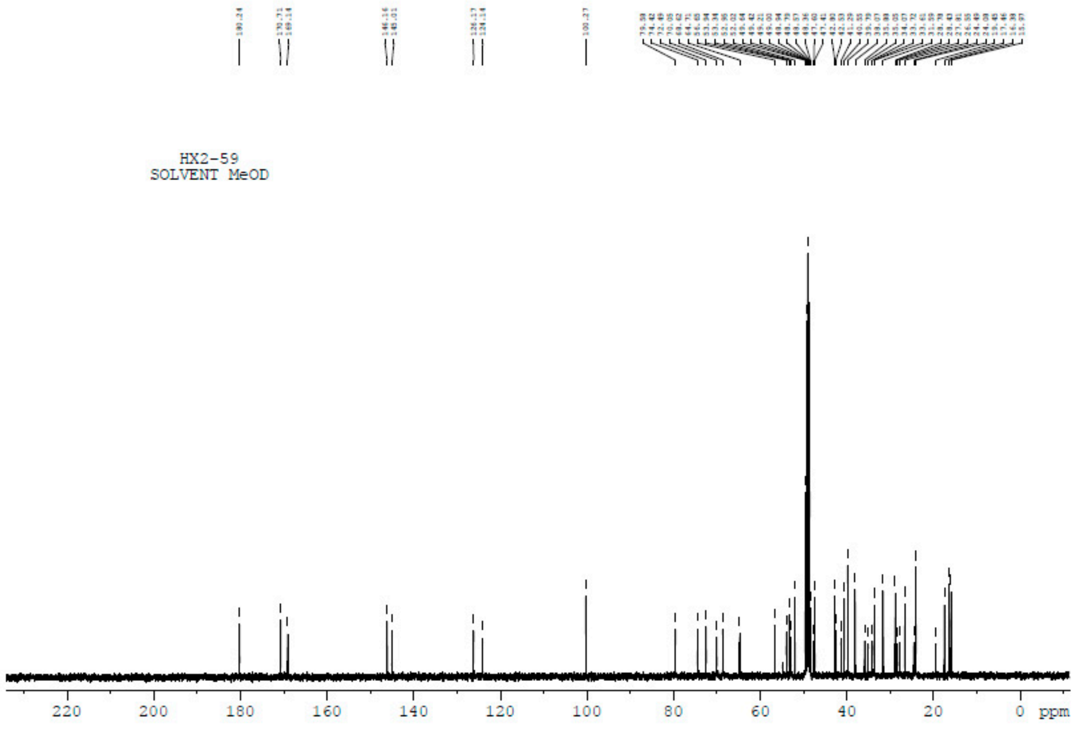

9. HR-ESI-MS spectrum of compound **19**

HIGH RESLUTION MASS SPECTROMETRY REPORT

Sample No.	Formula (M)	Ion Formula	Measured m/z	Calc m/z	Diff (ppm)
HX2-59	C46 H73 N5 O11	C46 H73 N5 Na O11	894.5206	894.5199	-1.23

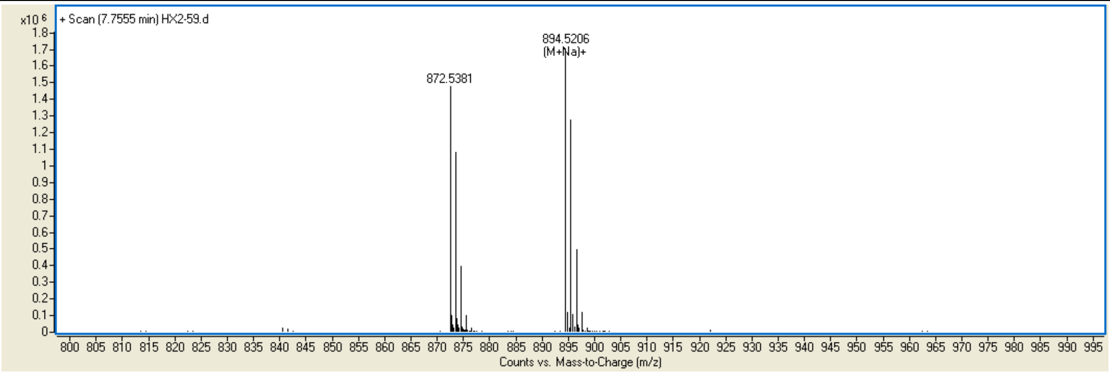

10. ^{1}H NMR (400 MHz, $CDCl_3$) spectrum of compound **20**

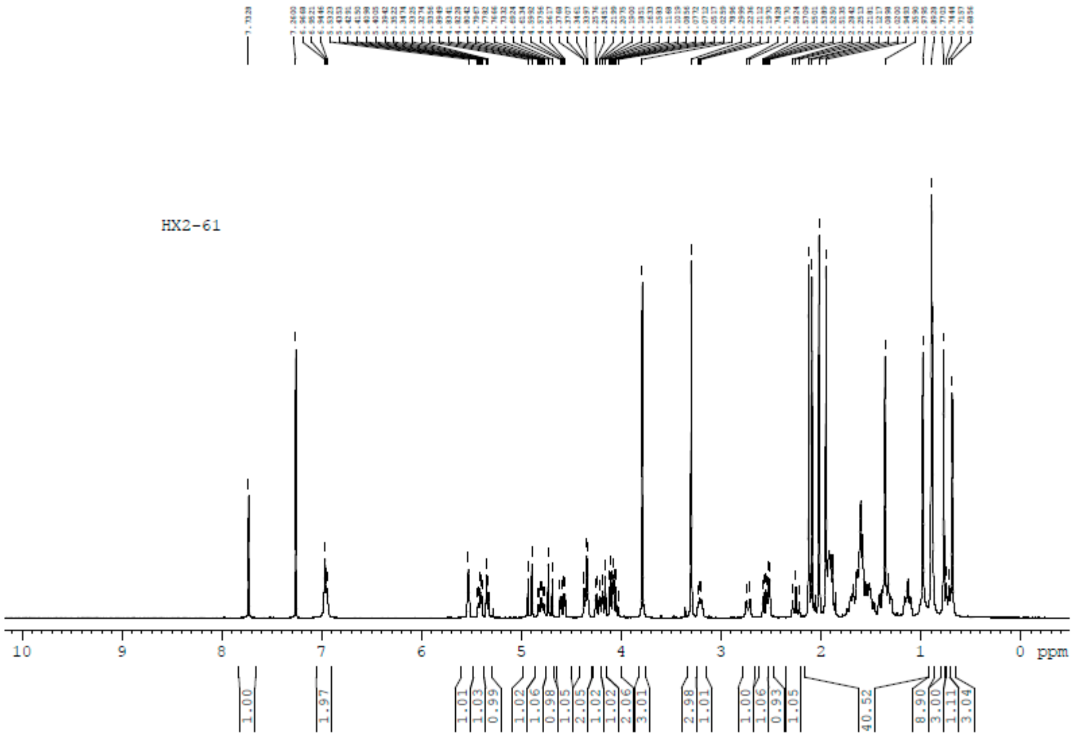

11. ^{13}C NMR (100 MHz, $CDCl_3$) spectrum of compound **20**

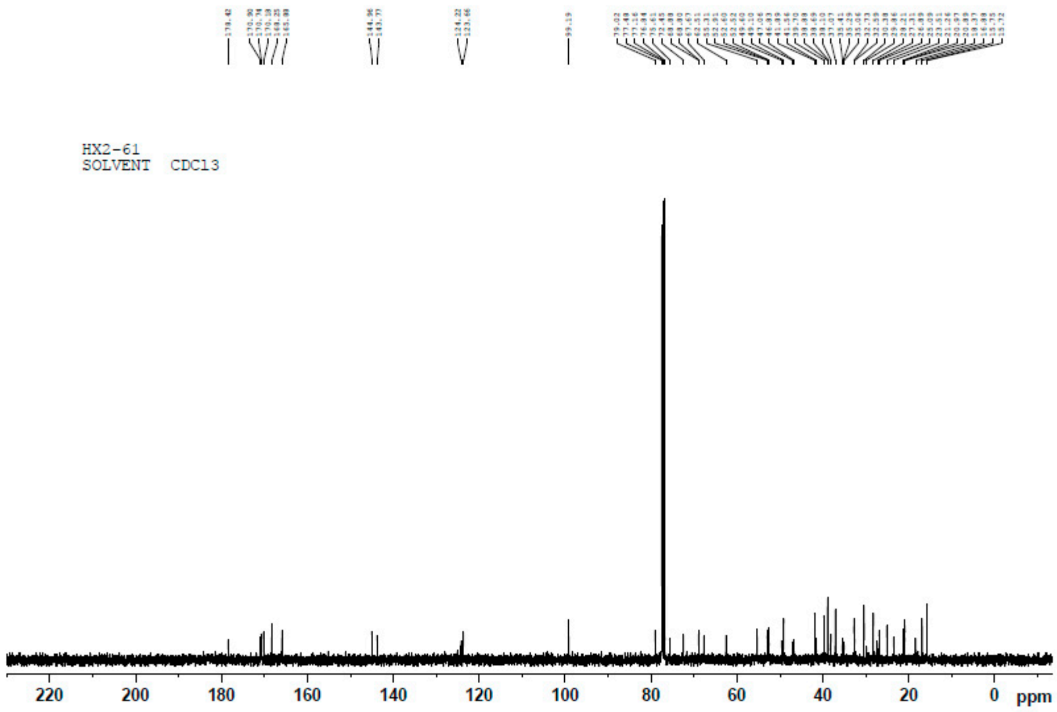

12. HR-ESI-MS spectrum of compound **20**

HIGH RESLUTION MASS SPECTROMETRY REPORT

Sample No.	Formula (M)	Ion Formula	Measured m/z	Calc m/z	Diff (ppm)
HX2-61	C54 H81 N5 O16	C54 H81 N5 Na O16	1078.558	1078.5571	-1.01

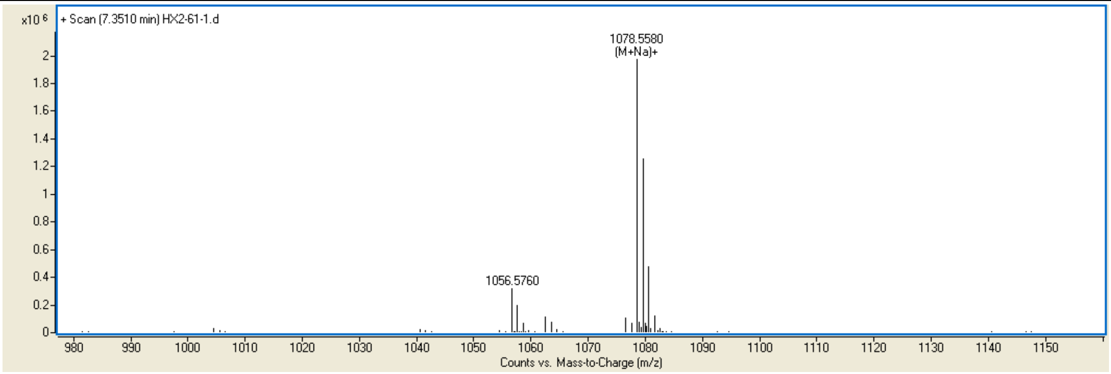

13. ^{1}H NMR (400 MHz, CD_3OD) spectrum of compound **21**

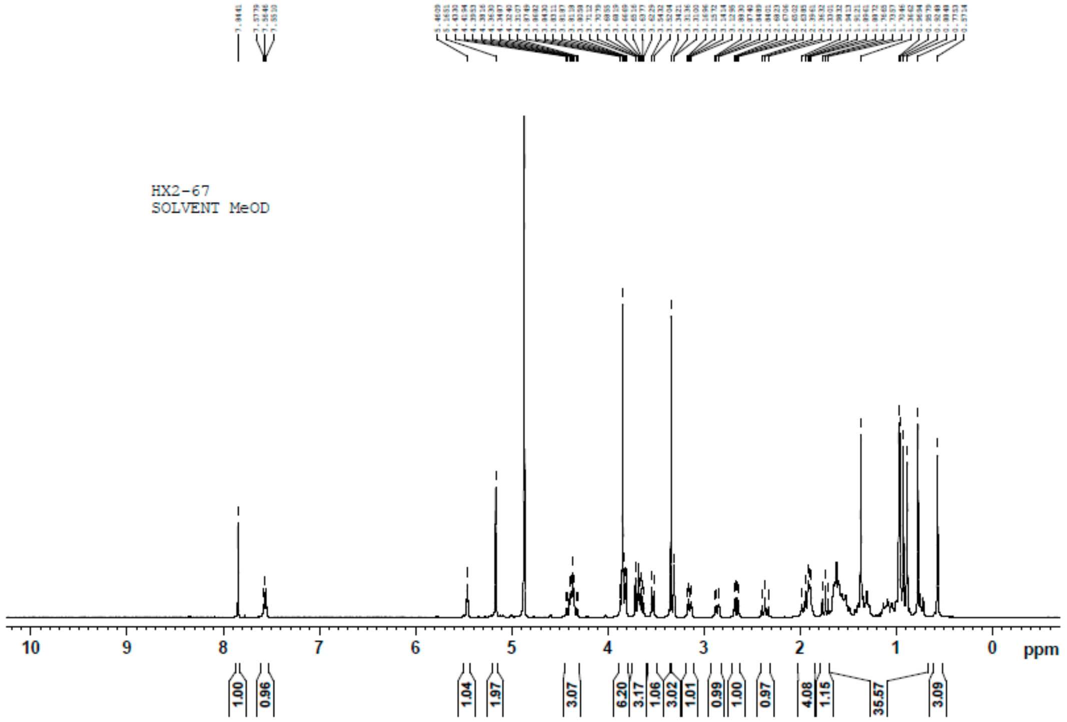

14. ^{13}C NMR (100 MHz, CD_3OD) spectrum of compound **21**

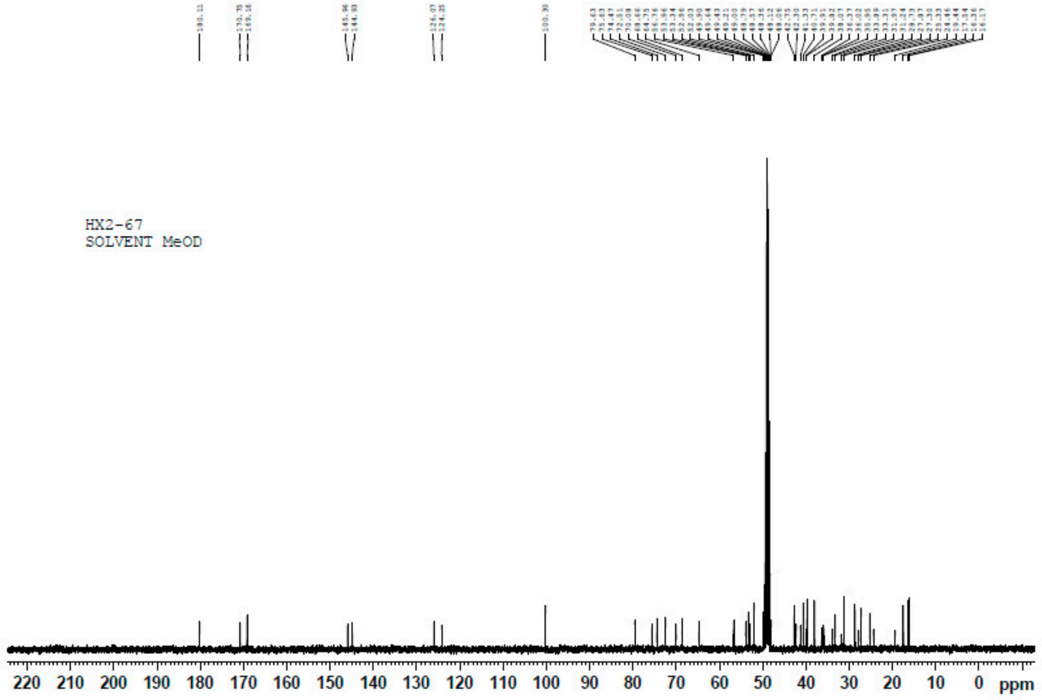

15. HR-ESI-MS spectrum of compound **21**

HIGH RESLUTION MASS SPECTROMETRY REPORT

Sample No.	Formula (M)	Ion Formula	Measured m/z	Calc m/z	Diff (ppm)
HX2-67	C46 H73 N5 O12	C46 H73 N5 Na O12	910.5152	910.5148	-0.77

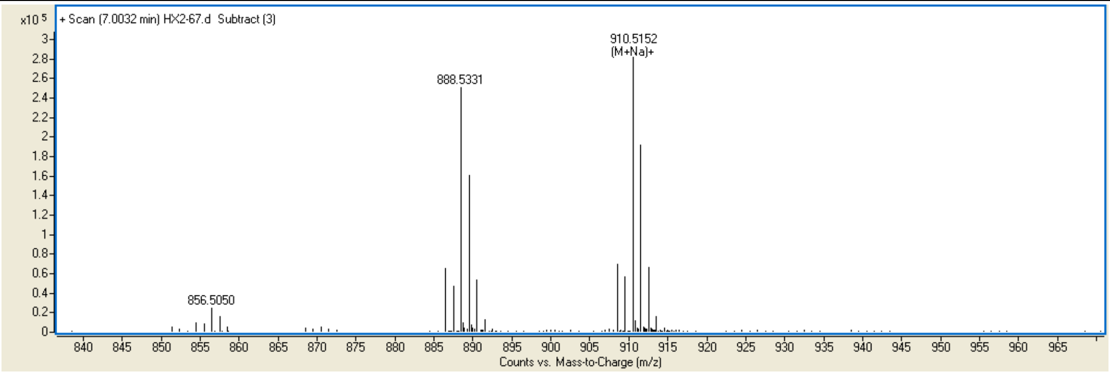

16. ^{1}H NMR (400 MHz, $CDCl_3$) spectrum of compound **22**

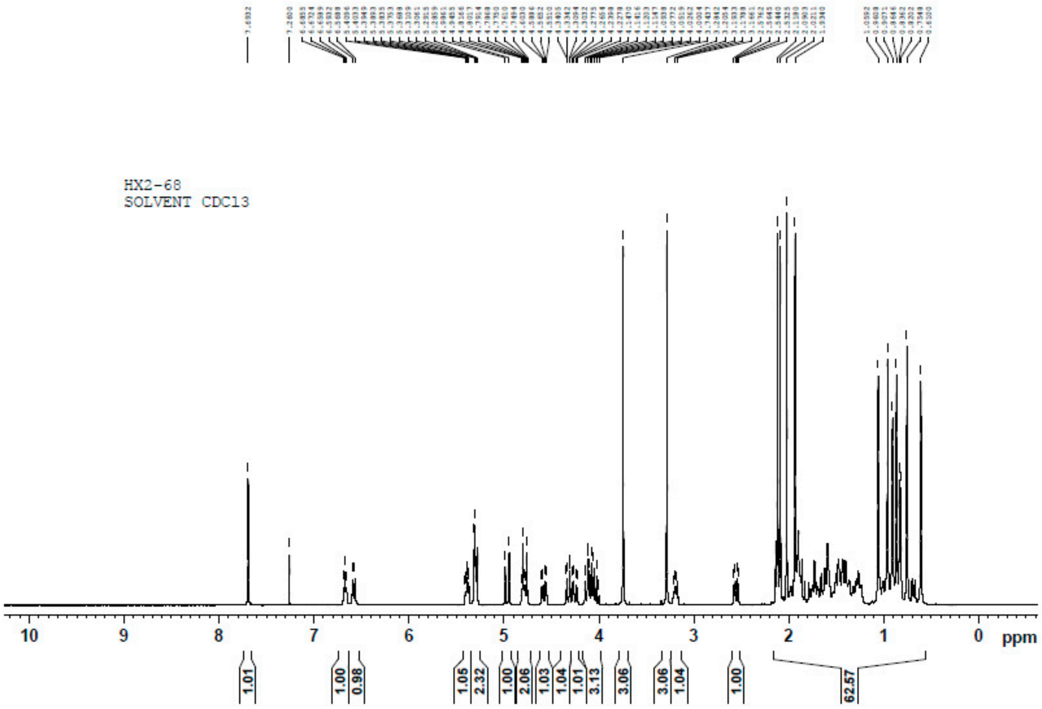

17. ^{13}C NMR (100 MHz, $CDCl_3$) spectrum of compound **22**

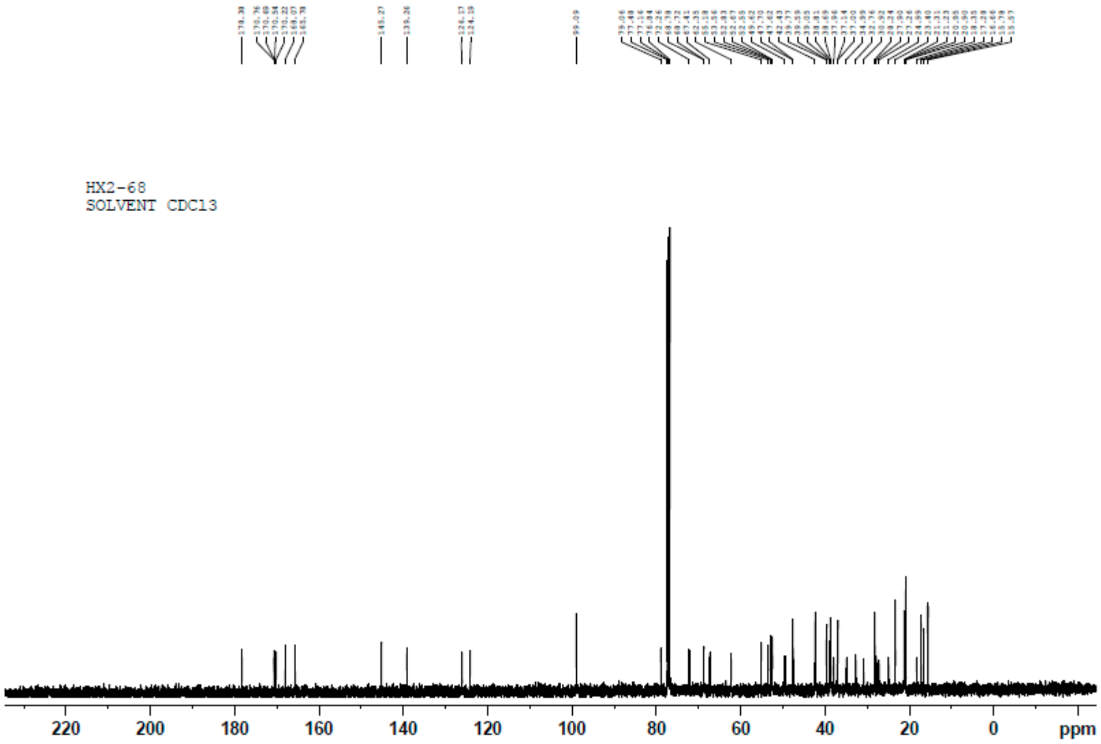

18. HR-ESI-MS spectrum of compound **22**

HIGH RESLUTION MASS SPECTROMETRY REPORT

Sample No.	Formula (M)	Ion Formula	Measured m/z	Calc m/z	Diff (ppm)
HX2-68	C54 H81 N5 O15	C54 H82 N5 O15	1040.5807	1040.58	-0.34

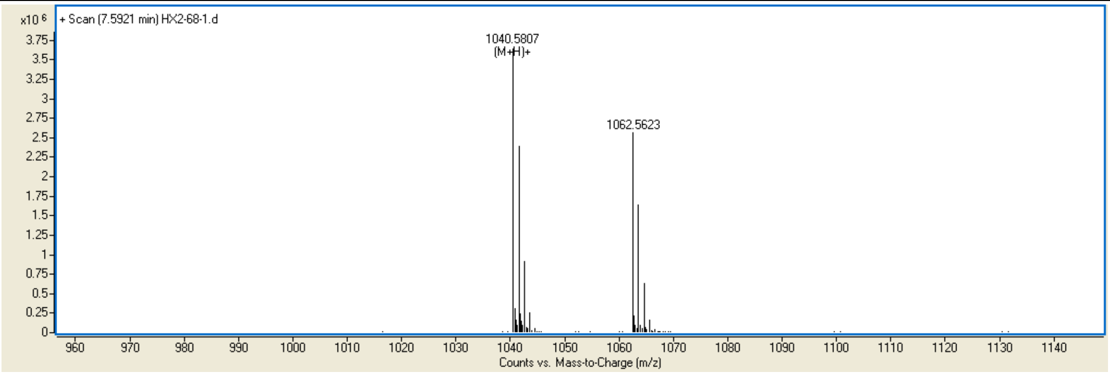

19. ^{1}H NMR (400 MHz, CD_3OD) spectrum of compound **23**

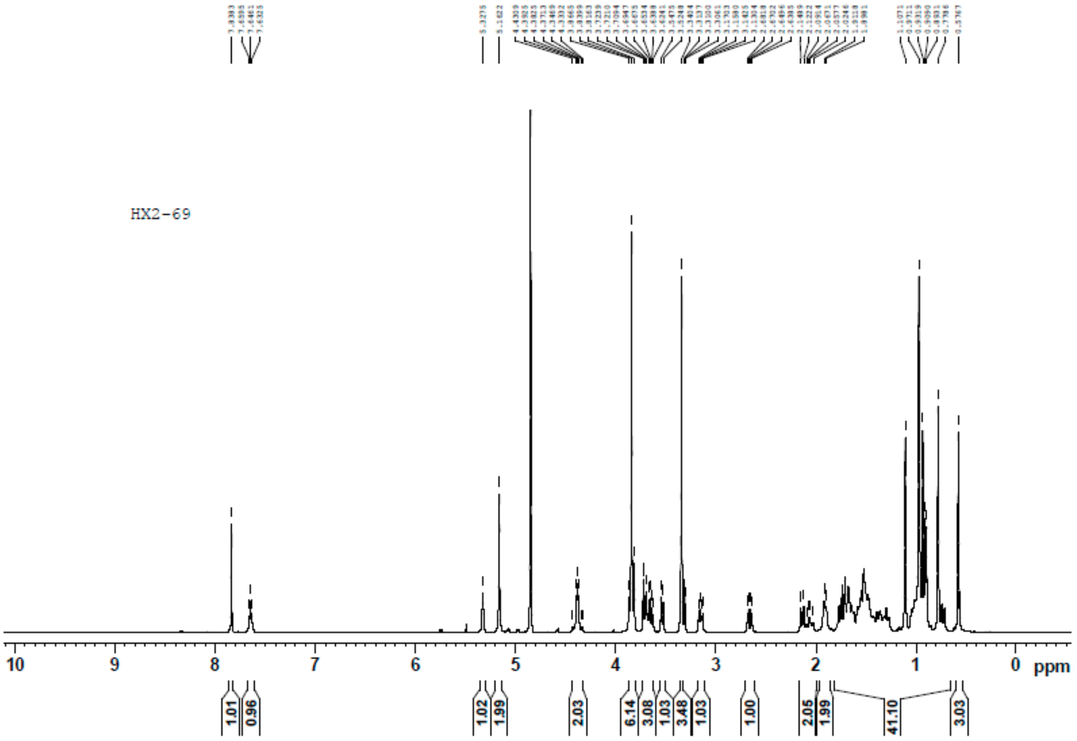

20. ^{13}C NMR (100 MHz, CD_3OD) spectrum of compound **23**

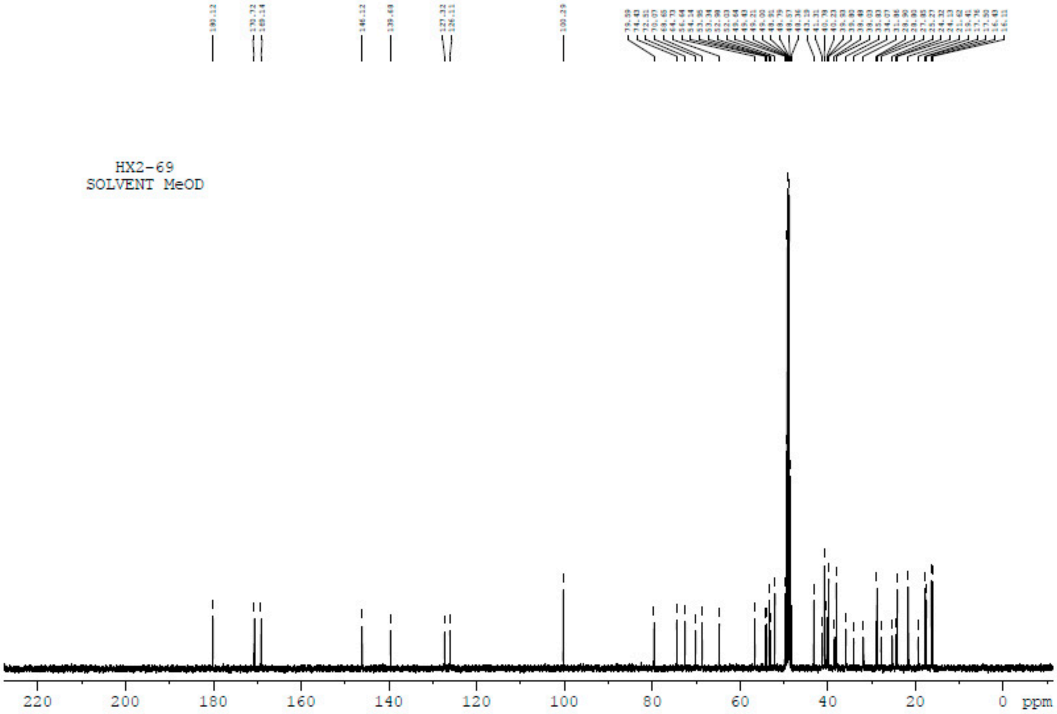

21. HR-ESI-MS spectrum of compound **23**

HIGH RESLUTION MASS SPECTROMETRY REPORT

Sample No.	Formula (M)	Ion Formula	Measured m/z	Calc m/z	Diff (ppm)
HX2-69	C46 H73 N5 O11	C46 H73 N5 Na O11	894.5198	894.5199	0.03

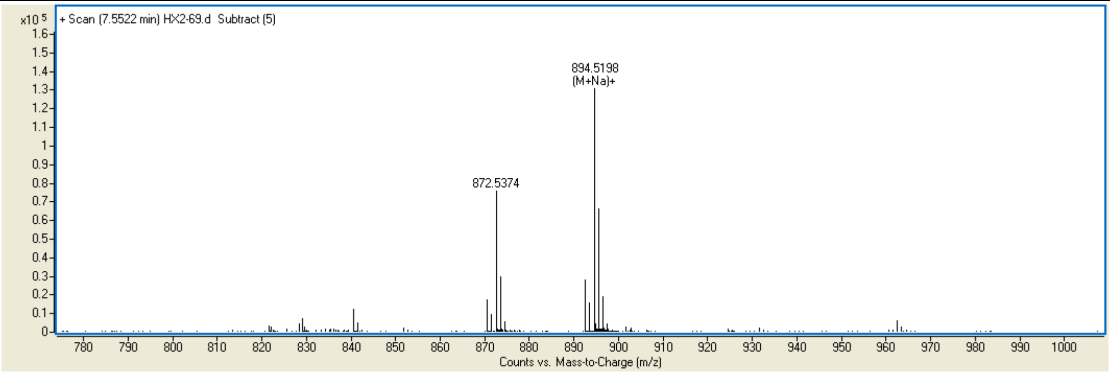

22. ^{1}H NMR (400 MHz, CD_3OD) spectrum of compound **24**

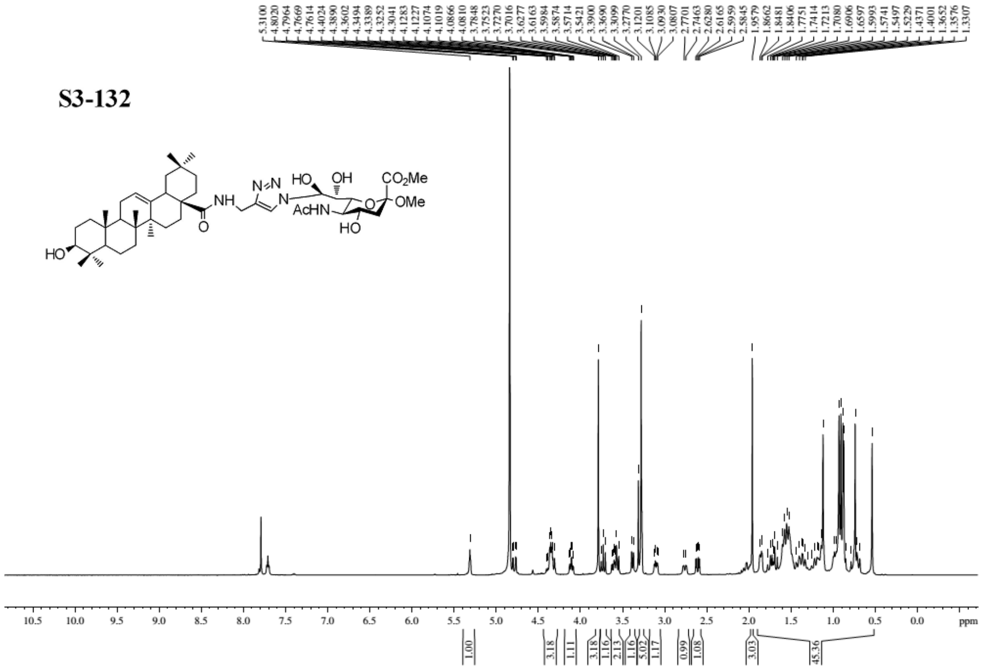

23. ^{13}C NMR (100 MHz, CD_3OD) spectrum of compound **24**

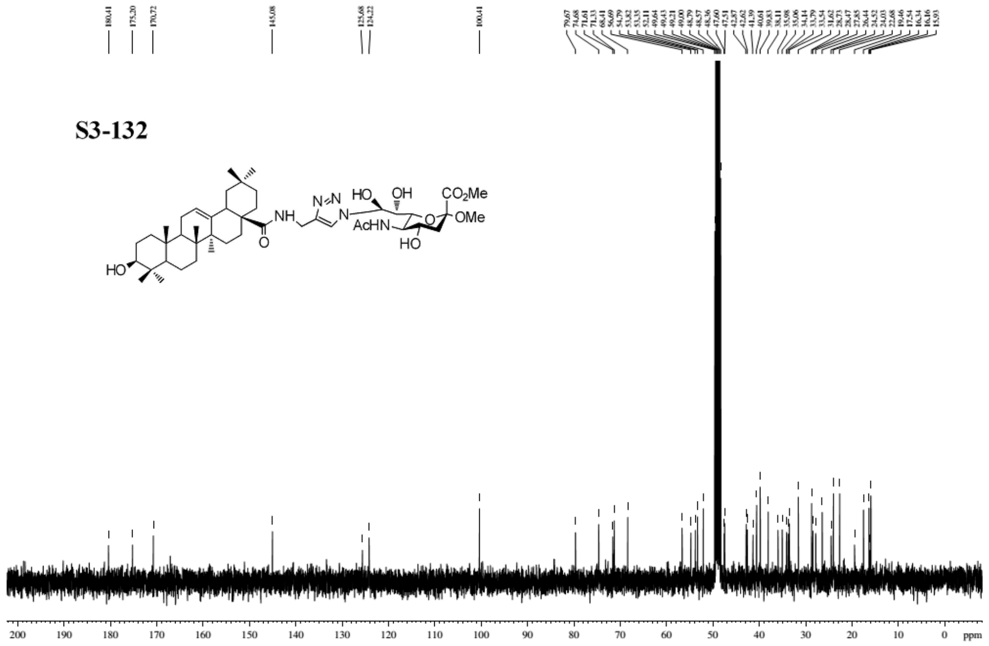

24. HR-ESI-MS spectrum of compound **24**

Peking University Mass Spectrometry Sample Analysis Report

Analysis Info

Analysis Name	14040508_20140411_000027.d	Acquisition Date	4/11/2014 10:12:17 AM
Sample	S3-132	Instrument	Bruker Apex IV FTMS
Comment	ESI Positive	Operator	Peking University

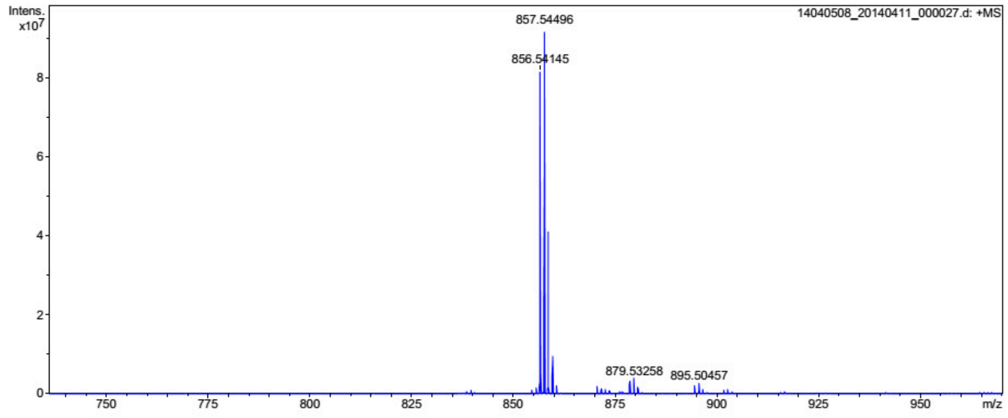

Meas. m/z	I	Formula	m/z	err [mDa]	err [ppm]	mSigma	rdb	e⁻ Conf	N-Rule
856.54145	81536448	C 46 H 74 N 5 O 10	856.54302	1.57	1.84	287.6	12.5	even	ok

25. ^{1}H NMR (400 MHz, CD_3OD) spectrum of compound **25**

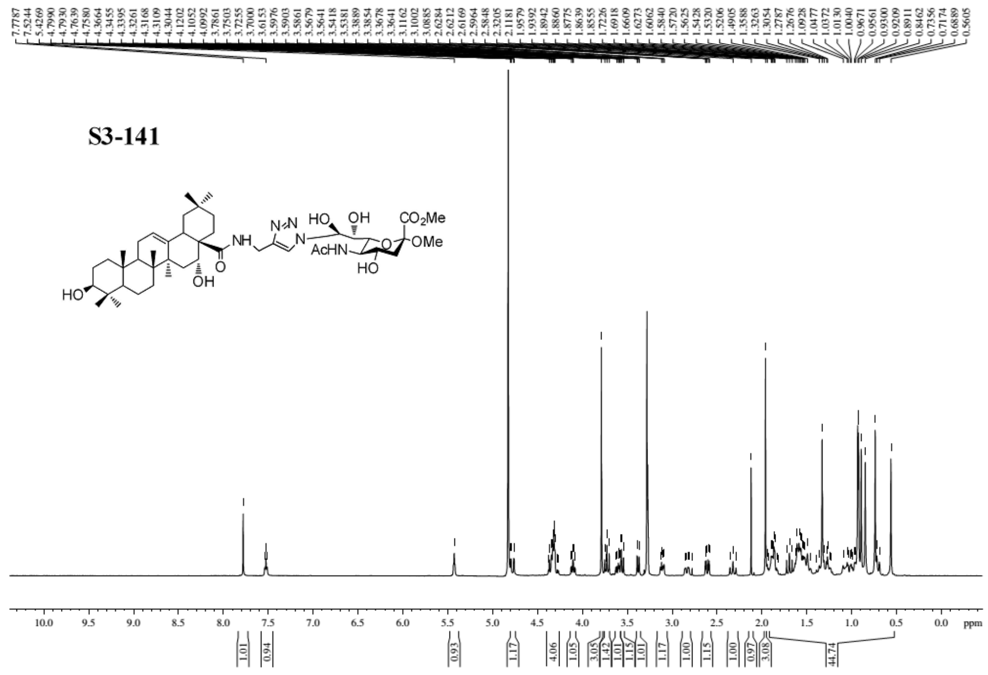

26. ^{13}C NMR (100 MHz, CD_3OD) spectrum of compound **25**

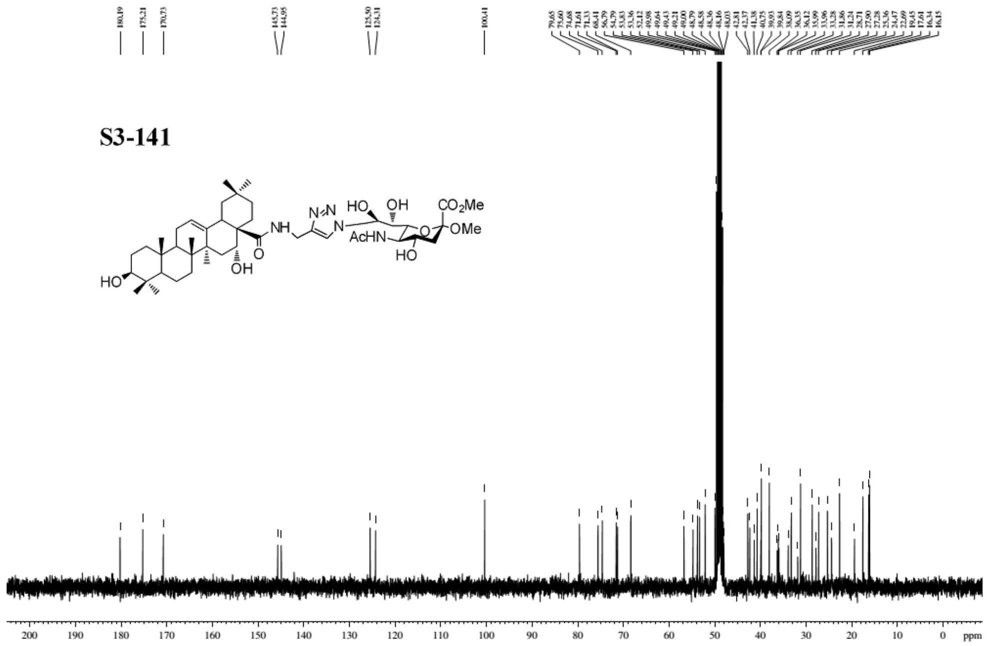

27. HR-ESI-MS spectrum of compound **25**

Peking University Mass Spectrometry Sample Analysis Report

Analysis Info

Analysis Name	14040508_20140411_000029.d	Acquisition Date	4/11/2014 10:15:50 AM
Sample	S3-141	Instrument	Bruker Apex IV FTMS
Comment	ESI Positive	Operator	Peking University

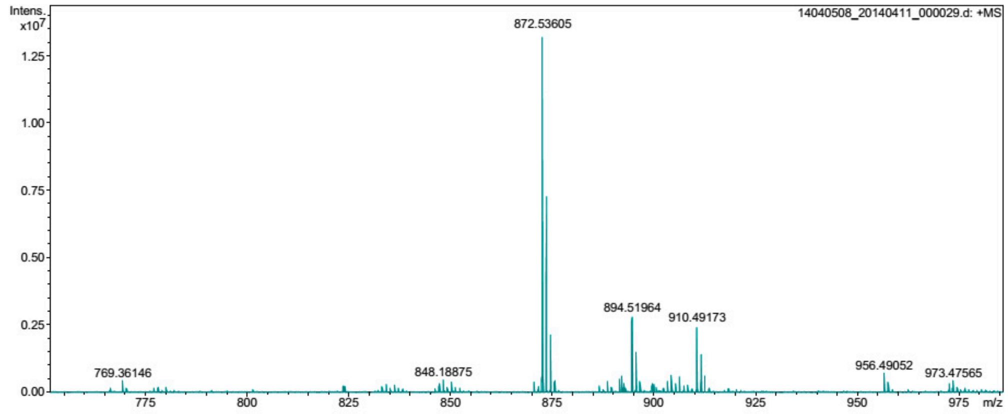

Meas. m/z	I	Formula	m/z	err [mDa]	err [ppm]	mSigma	rdb	e⁻ Conf	N-Rule
872.53605	13413647	C 46 H 74 N 5 O 11	872.53793	1.89	2.16	18.3	12.5	even	ok

28. ^{1}H NMR (400 MHz, CD_3OD) spectrum of compound **26**

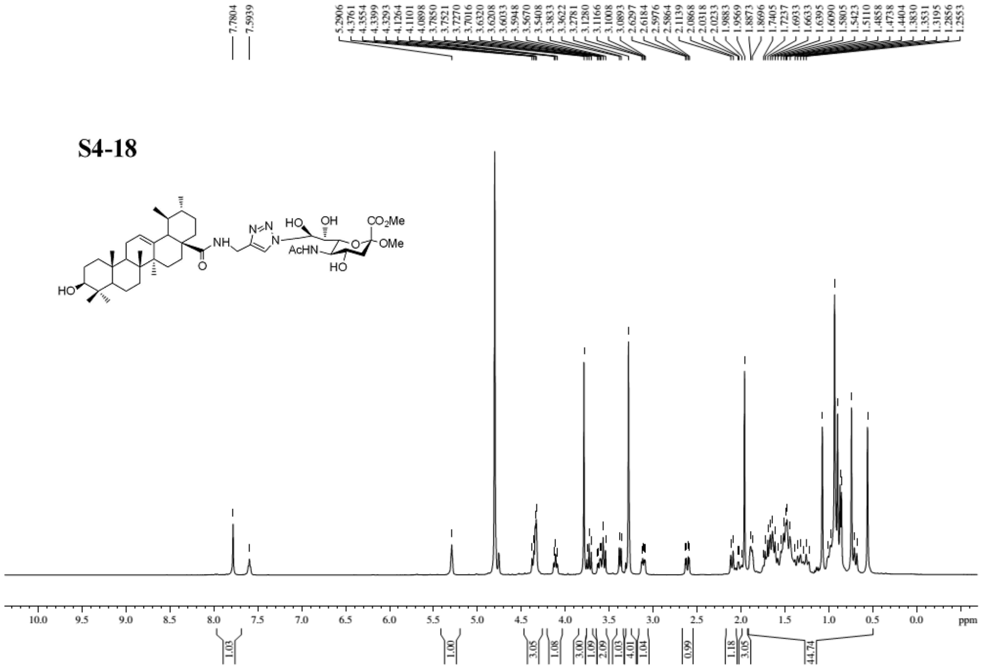

29. ^{13}C NMR (100 MHz, CD_3OD) spectrum of compound **26**

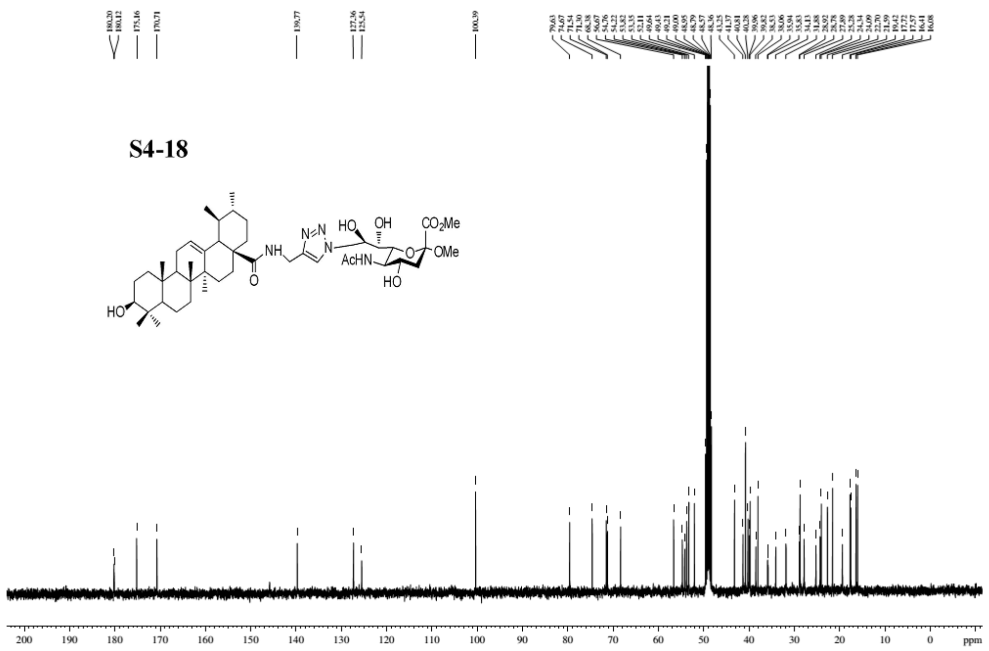

30. HR-ESI-MS spectrum of compound **26**

Peking University Mass Spectrometry Sample Analysis Report

Analysis Info

Analysis Name	14040508_20140411_000024.d	Acquisition Date	4/11/2014 10:06:48 AM
Sample	S4-18	Instrument	Bruker Apex IV FTMS
Comment	ESI Positive	Operator	Peking University

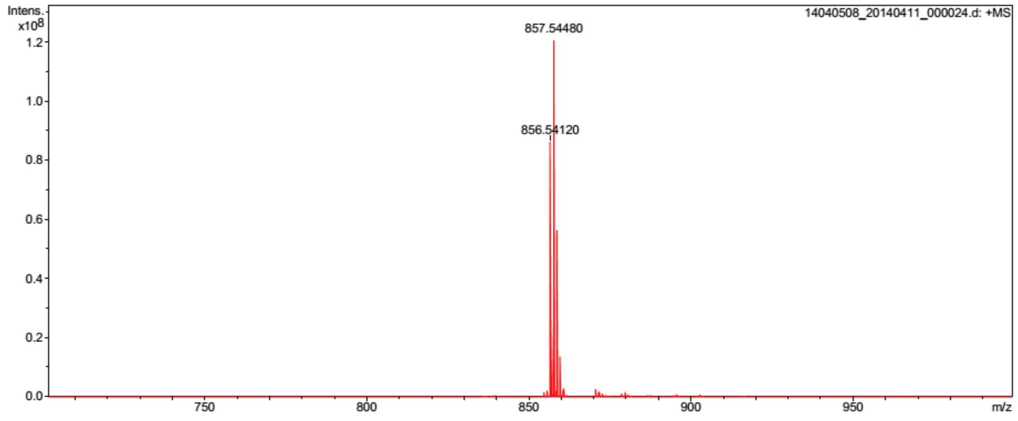

Meas. m/z	I	Formula	m/z	err [mDa]	err [ppm]	mSigma	rdb	e⁻ Conf	N-Rule
856.54120	86363592	C 46 H 74 N 5 O 10	856.54302	1.82	2.12	323.3	12.5	even	ok

31. ^{1}H NMR (400 MHz, $CDCl_3$) spectrum of compound **28**

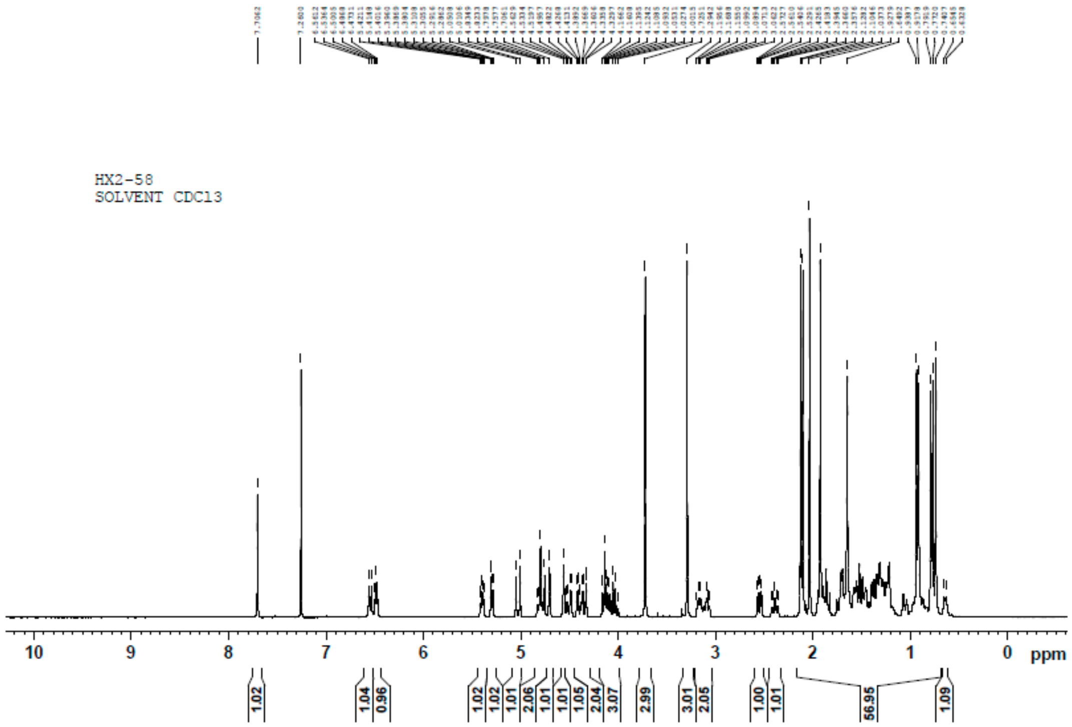

32. ^{13}C NMR (100 MHz, $CDCl_3$) spectrum of compound **28**

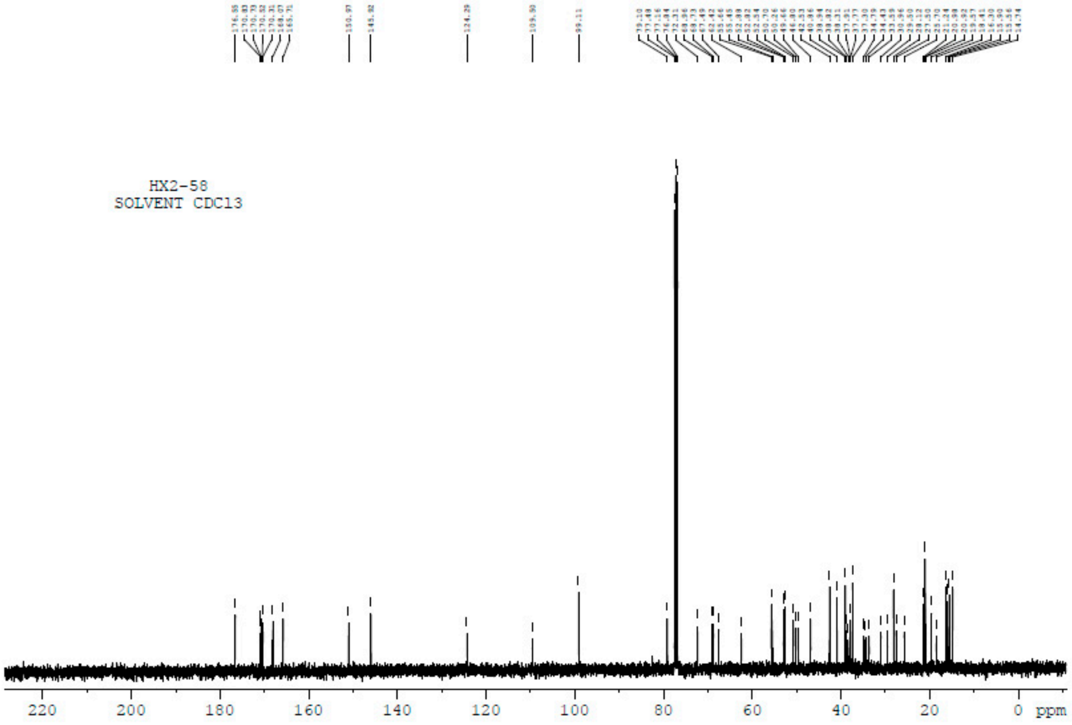

33. HR-ESI-MS spectrum of compound **28**

HIGH RESLUTION MASS SPECTROMETRY REPORT

Sample No.	Formula (M)	Ion Formula	Measured m/z	Calc m/z	Diff (ppm)
HX2-58	C54 H81 N5 O15	C54 H82 N5 O15	1040.5809	1040.5802	-0.59

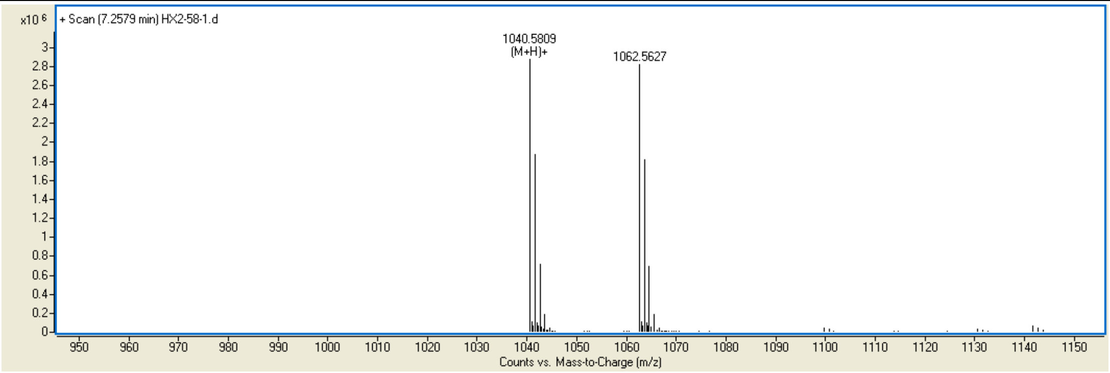

34. ^{1}H NMR (400 MHz, CD_3OD) spectrum of compound **29**

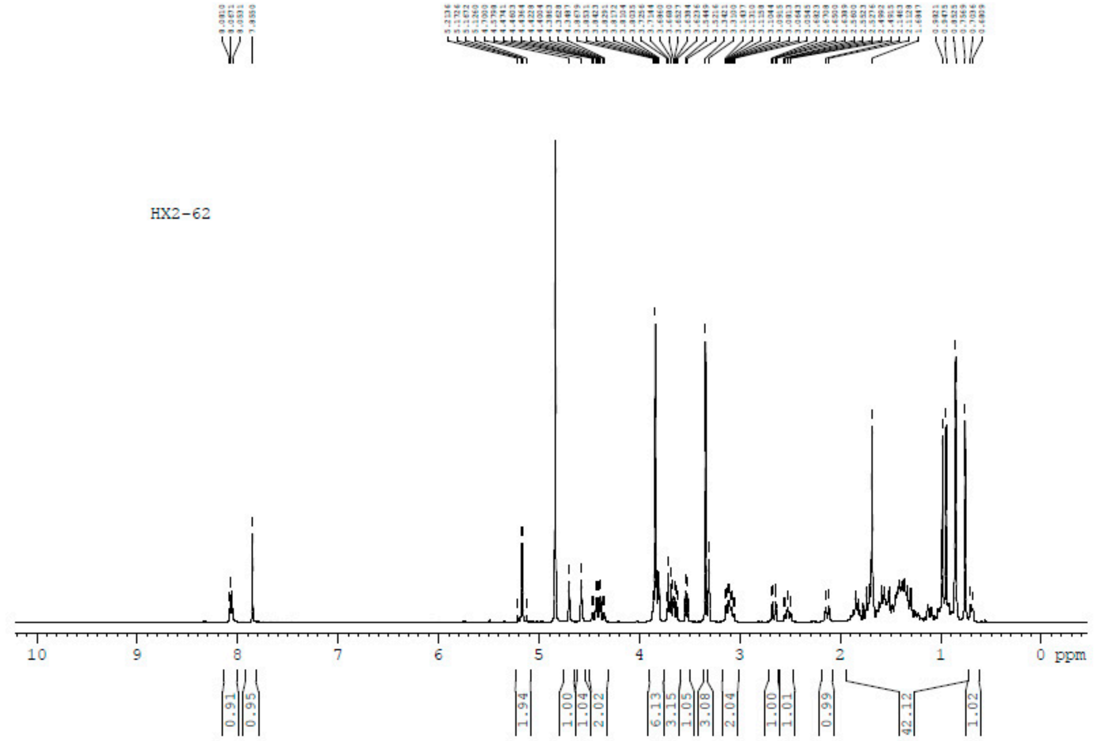

35. ^{13}C NMR (100 MHz, CD_3OD) spectrum of compound **29**

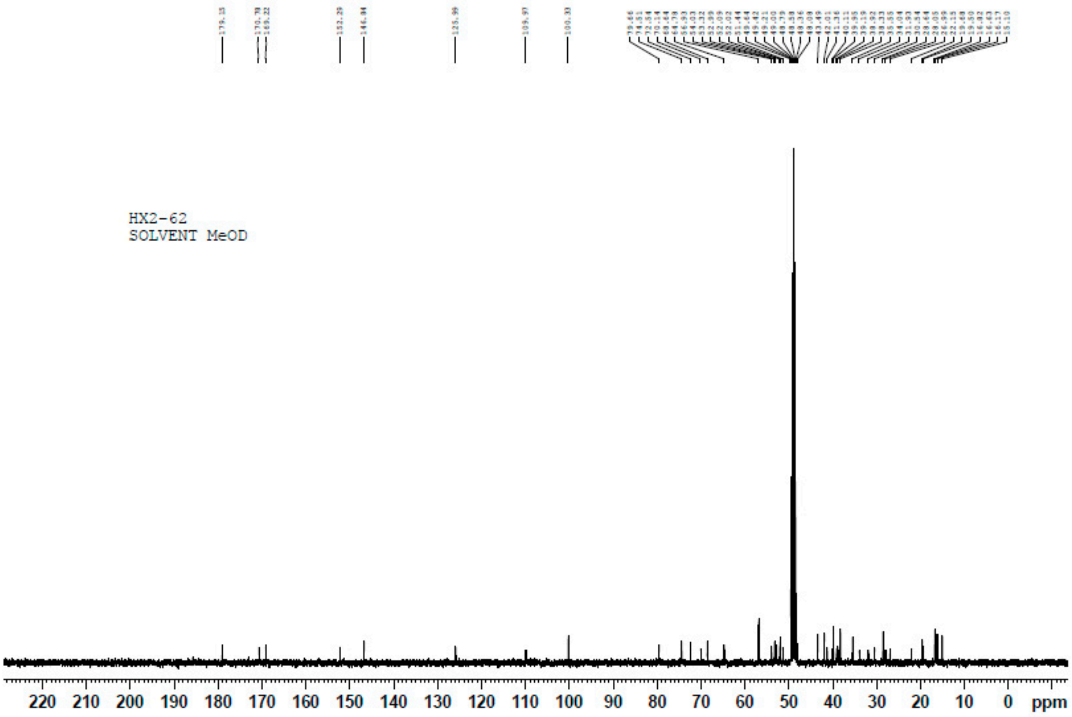

36. HR-ESI-MS spectrum of compound **29**

HIGH RESLUTION MASS SPECTROMETRY REPORT

Sample No.	Formula (M)	Ion Formula	Measured m/z	Calc m/z	Diff (ppm)
HX2-62	C46 H73 N5 O11	C46 H74 N5 O11	872.5385	872.5379	-0.73

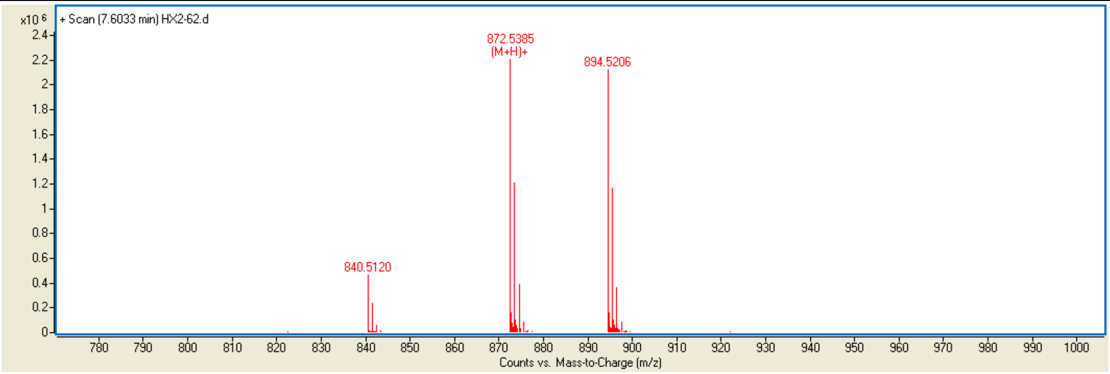

37. ^{1}H NMR (400 MHz, CD_3OD) spectrum of compound **30**

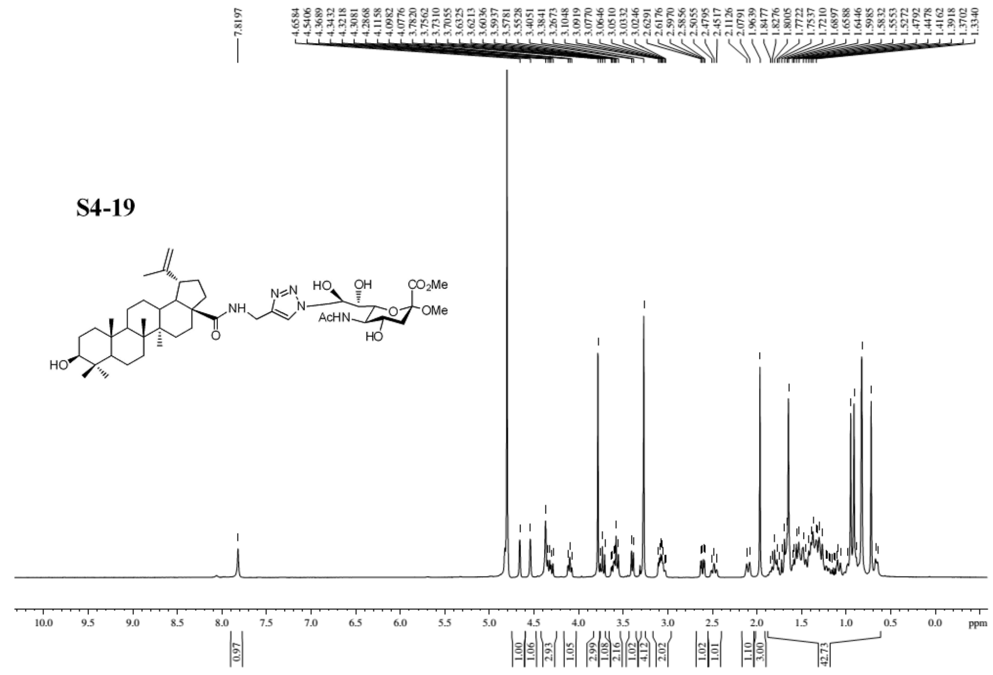

38. ^{13}C NMR (100 MHz, CD_3OD) spectrum of compound **30**

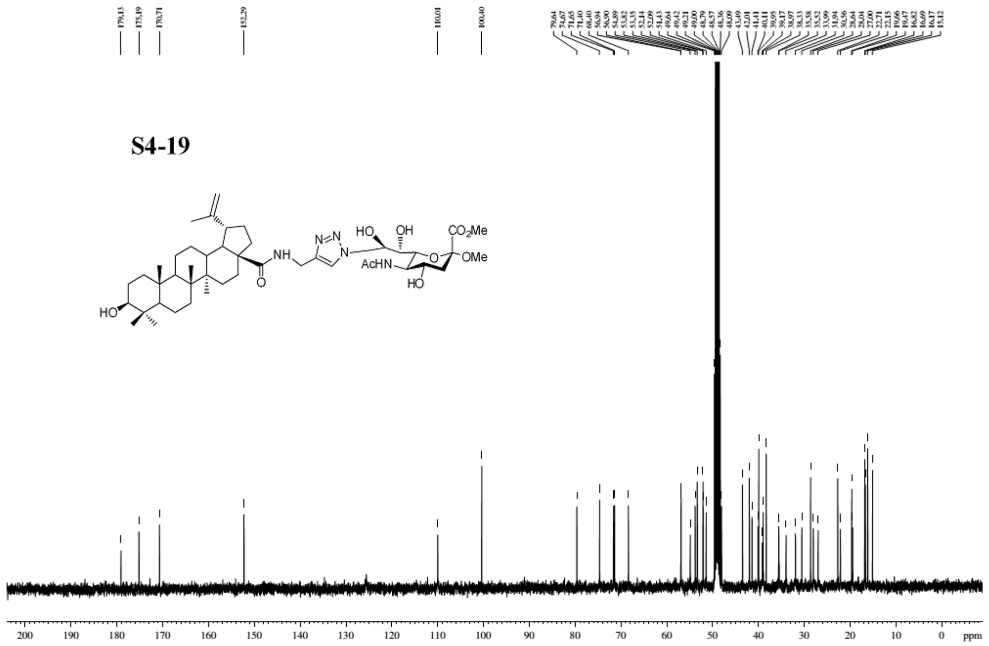

39. HR-ESI-MS spectrum of compound **30**

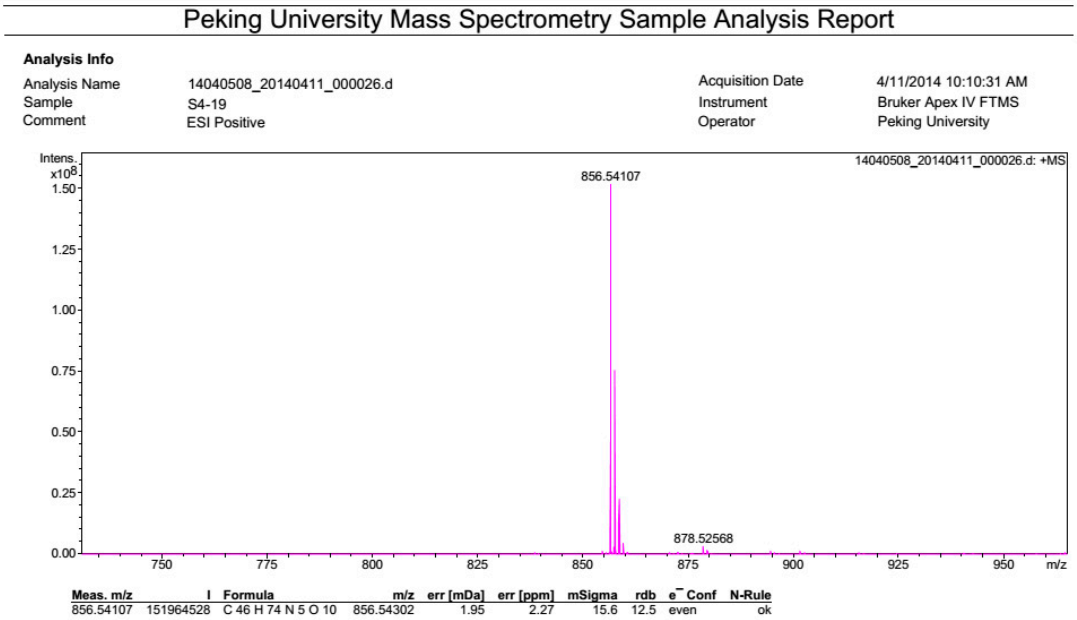

40. ^{1}H NMR (400 MHz, $CDCl_3$) spectrum of compound **34**

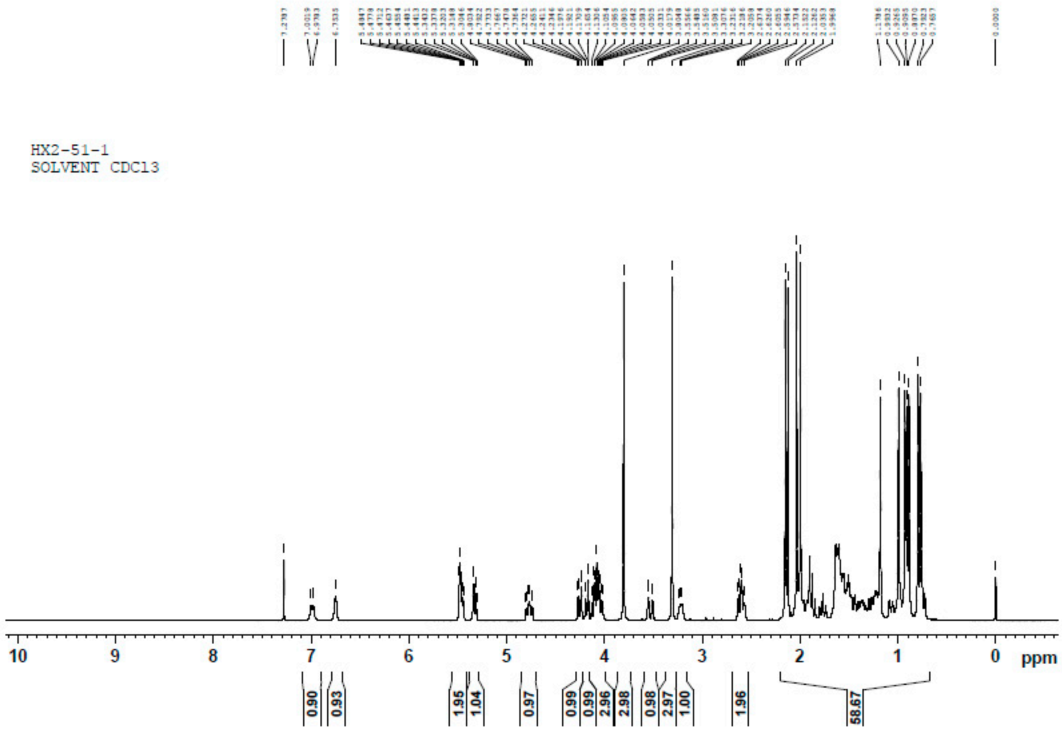

41. ^{13}C NMR (100 MHz, $CDCl_3$) spectrum of compound **34**

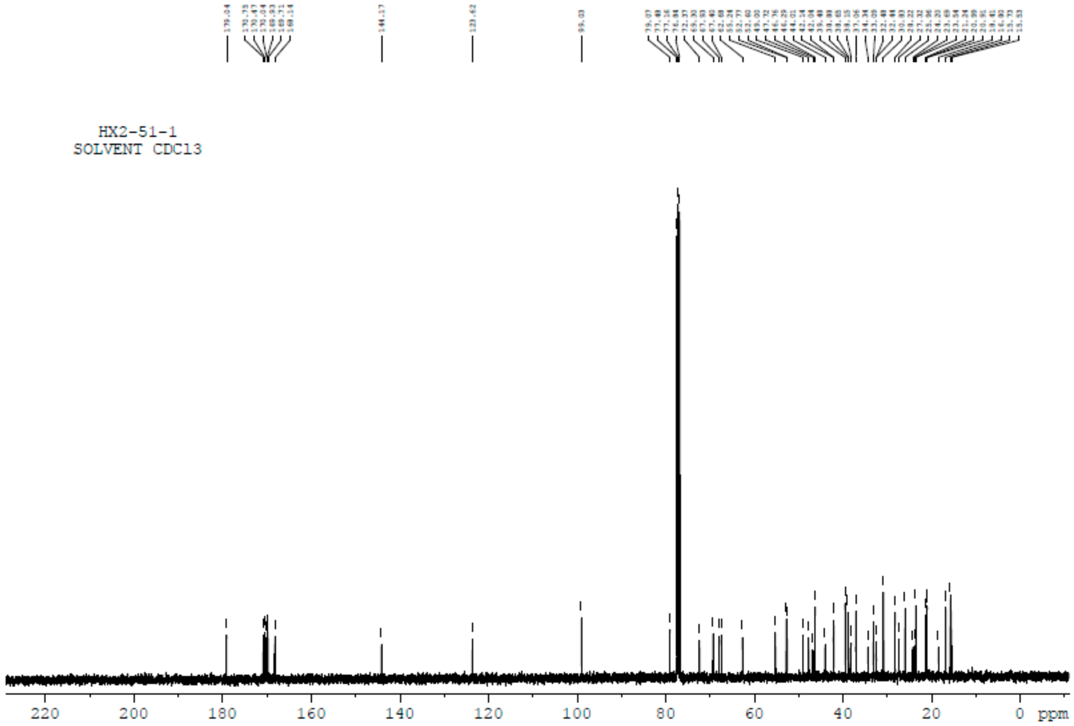

42. HR-ESI-MS spectrum of compound **34**

HIGH RESLUTION MASS SPECTROMETRY REPORT

Sample No.	Formula (M)	Ion Formula	Measured m/z	Calc m/z	Diff (ppm)
HX2-51	C51 H78 N2 O15	C51 H78 N2 Na O15	981.5294	981.5294	0

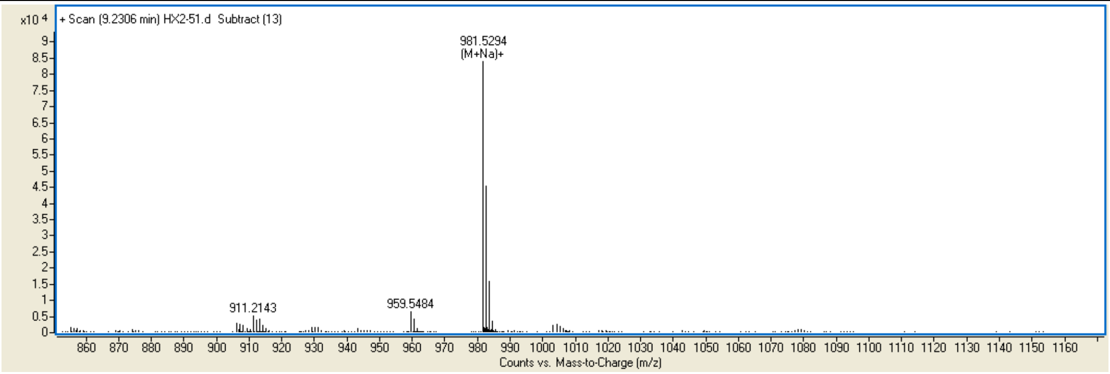

43. ^{1}H NMR (400 MHz, CD_3OD) spectrum of compound **35**

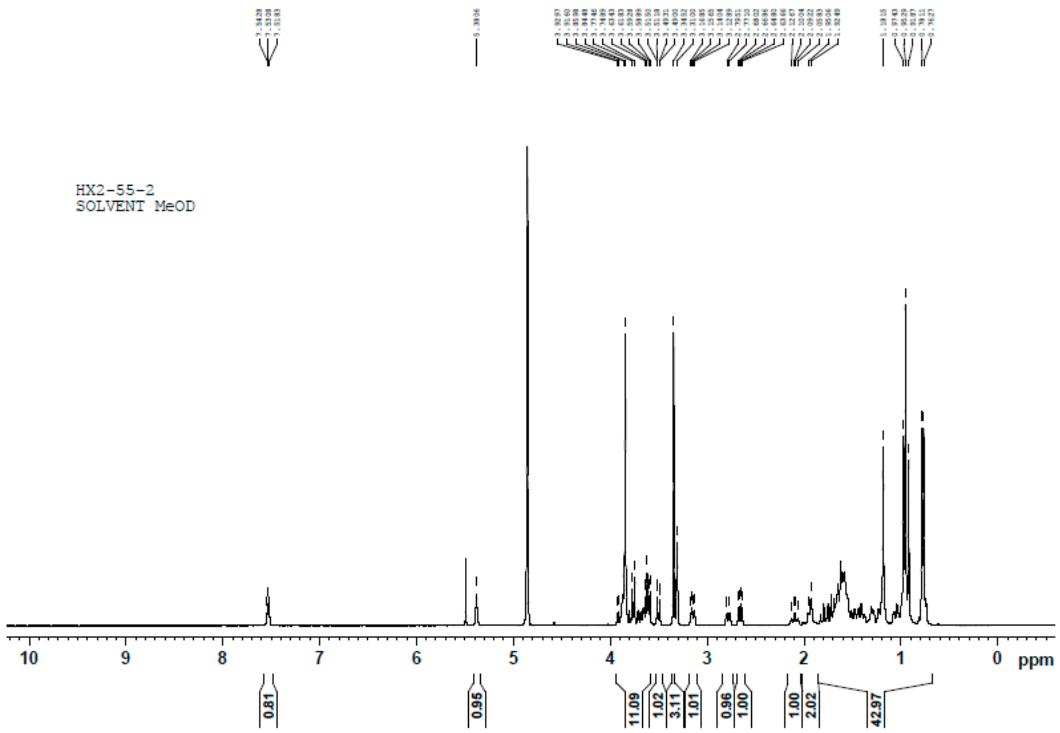

44. ^{13}C NMR (100 MHz, CD_3OD) spectrum of compound **35**

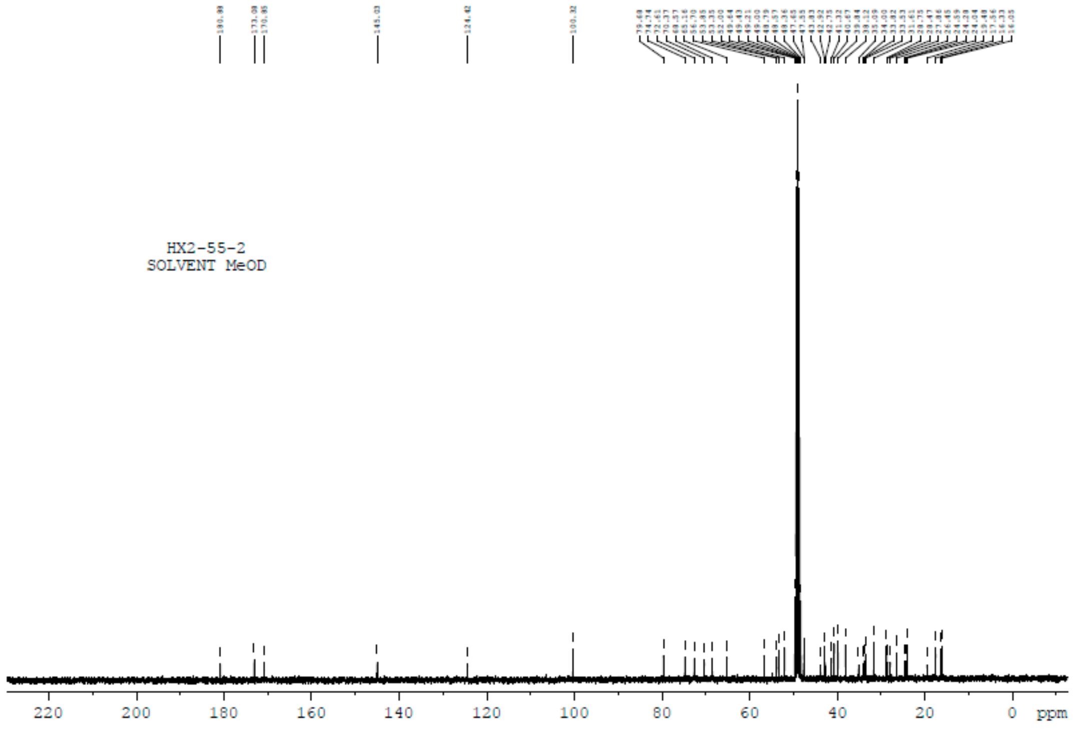

45. HR-ESI-MS spectrum of compound **35**

HIGH RESLUTION MASS SPECTROMETRY REPORT

Sample No.	Formula (M)	Ion Formula	Measured m/z	Calc m/z	Diff (ppm)
HX2-55-2	C43 H70 N2 O11	C43 H70 N2 Na O11	813.4876	813.4872	-0.39

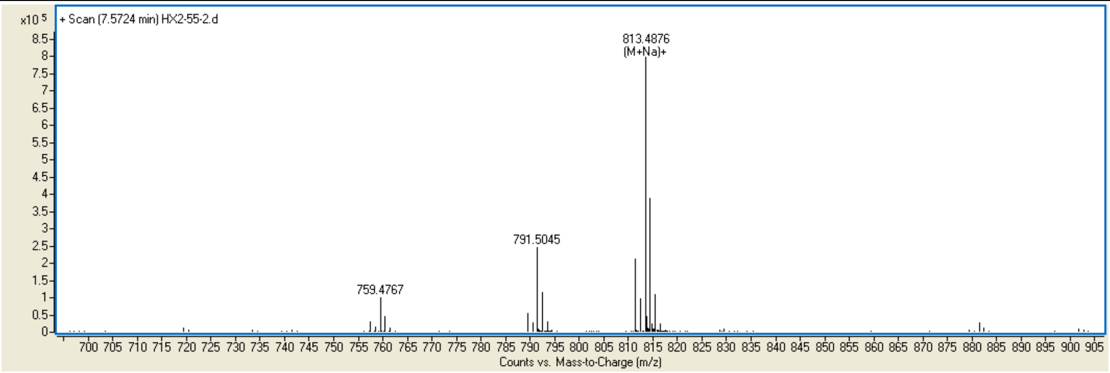

46. ^{1}H NMR (400 MHz, $CDCl_3$) spectrum of compound **36**

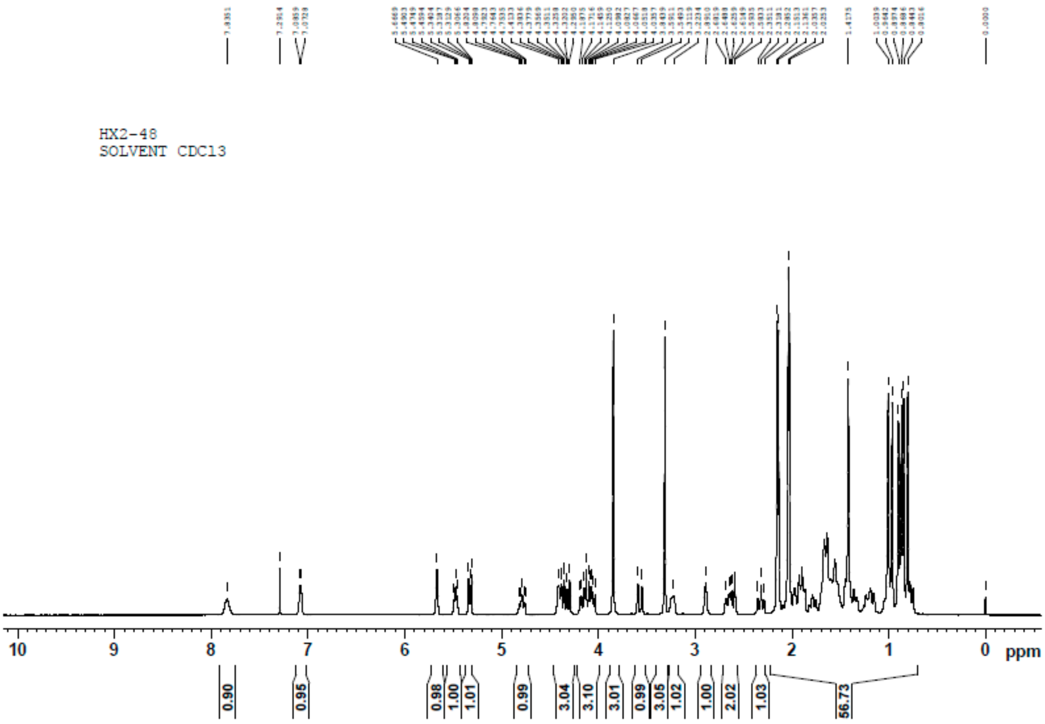

47. ^{13}C NMR (100 MHz, $CDCl_3$) spectrum of compound **36**

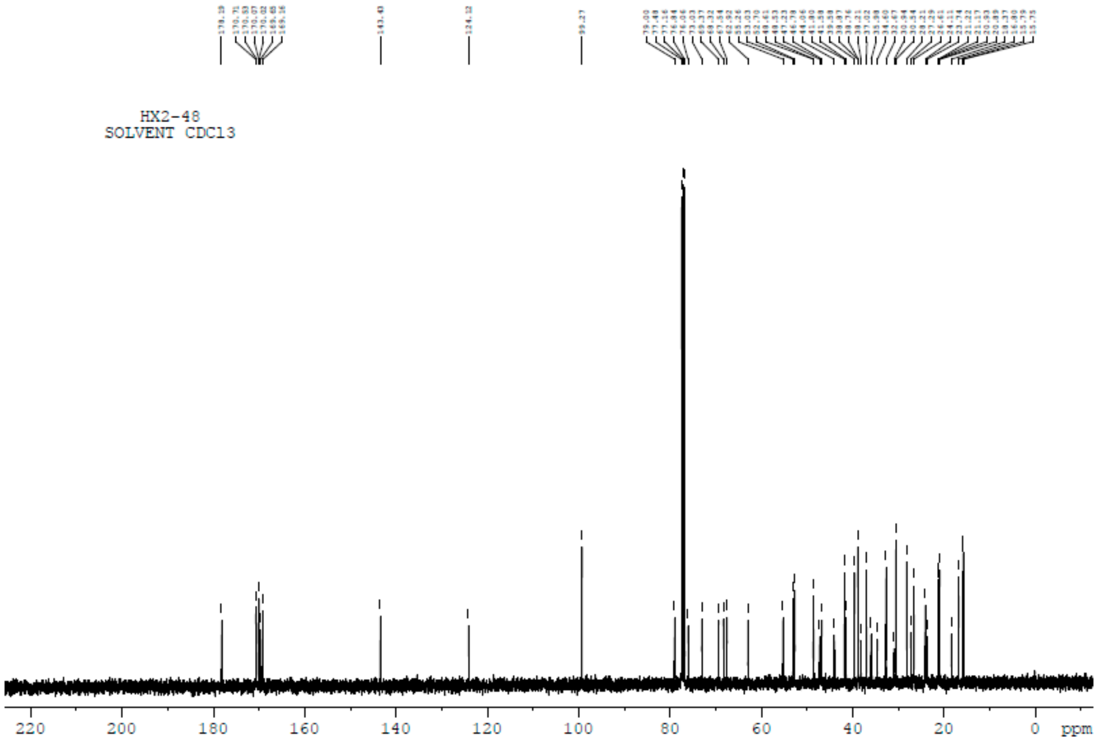

48. HR-ESI-MS spectrum of compound **36**

HIGH RESLUTION MASS SPECTROMETRY REPORT

Sample No.	Formula (M)	Ion Formula	Measured m/z	Calc m/z	Diff (ppm)
HX2-48	C51 H78 N2 O16	C51 H78 N2 Na O16	997.5252	997.5244	-0.83

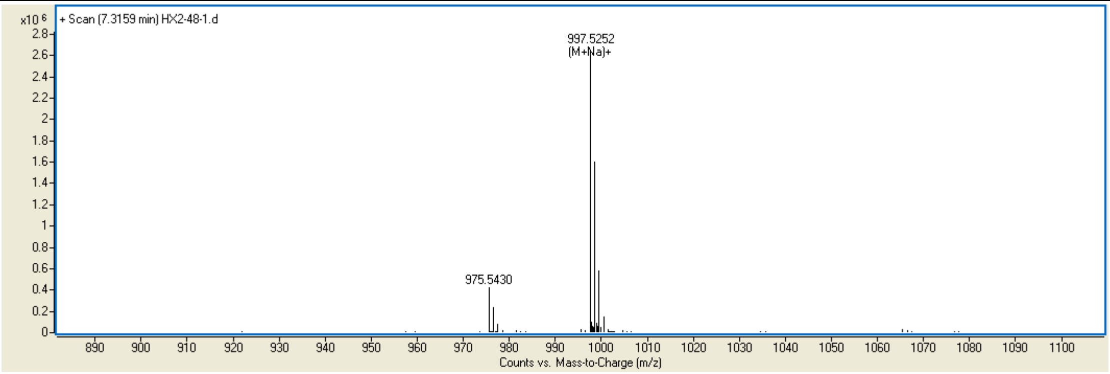

49. ^{1}H NMR (400 MHz, CD_3OD) spectrum of compound **37**

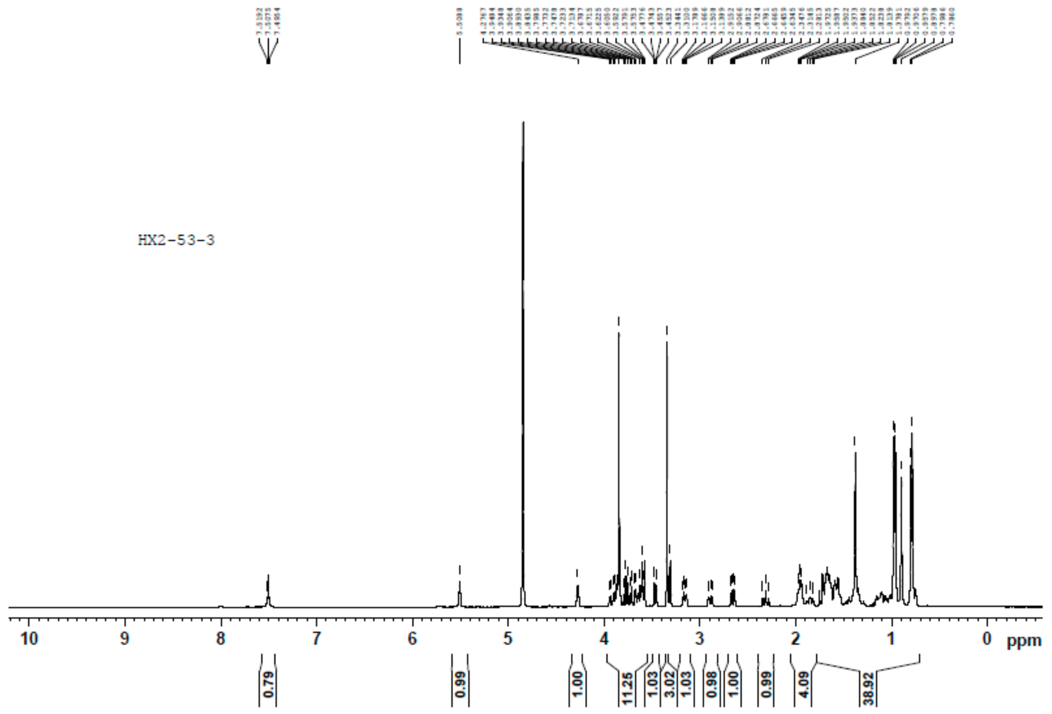

50. ^{13}C NMR (100 MHz, CD_3OD) spectrum of compound **37**

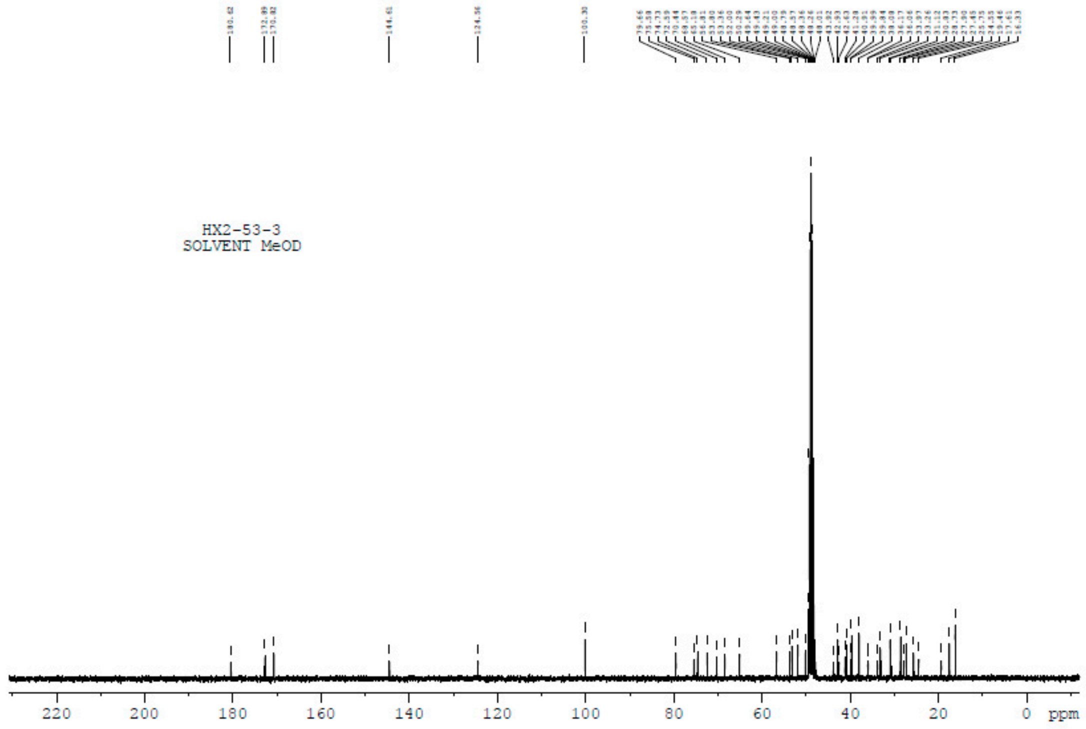

51. HR-ESI-MS spectrum of compound **37**

HIGH RESLUTION MASS SPECTROMETRY REPORT

Sample No.	Formula (M)	Ion Formula	Measured m/z	Calc m/z	Diff (ppm)
HX2-53-5	C43 H70 N2 O12	C43 H70 N2 Na O12	829.4826	829.4821	-0.84

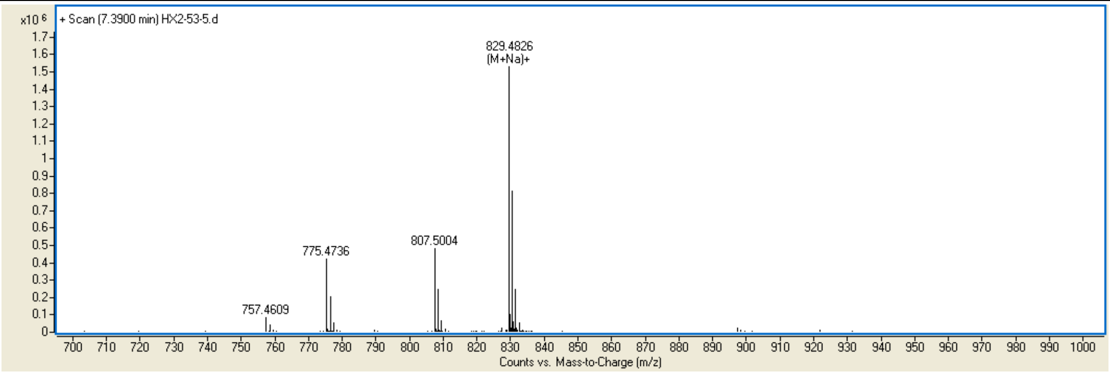

52. ^{1}H NMR (400 MHz, $CDCl_3$) spectrum of compound **38**

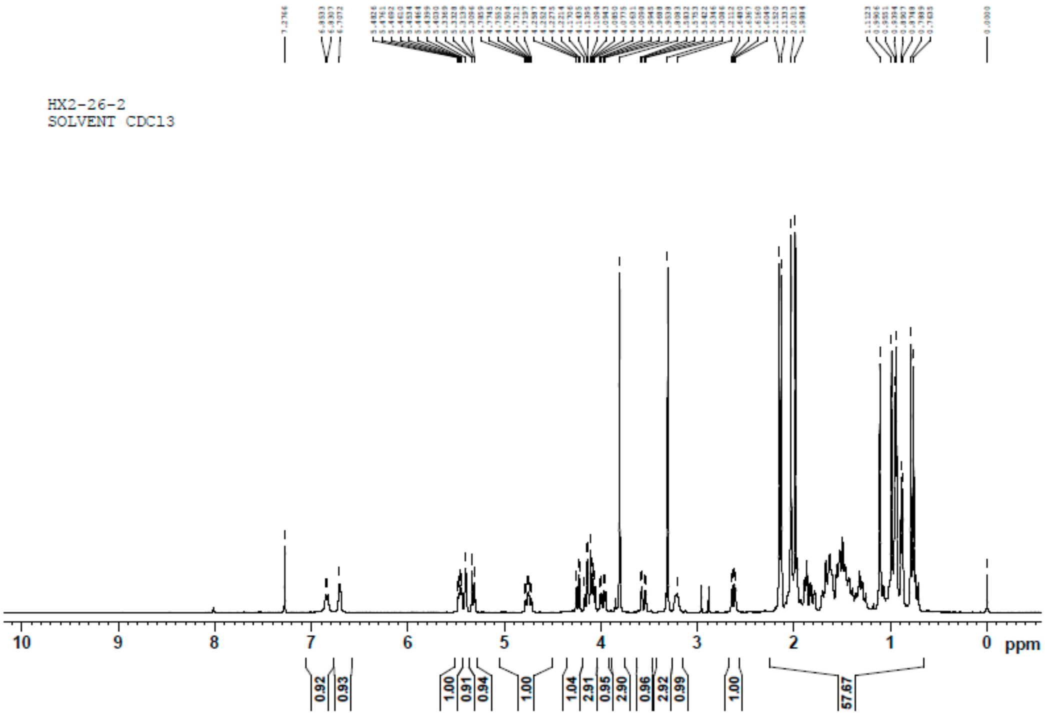

53. ^{13}C NMR (100 MHz, $CDCl_3$) spectrum of compound **38**

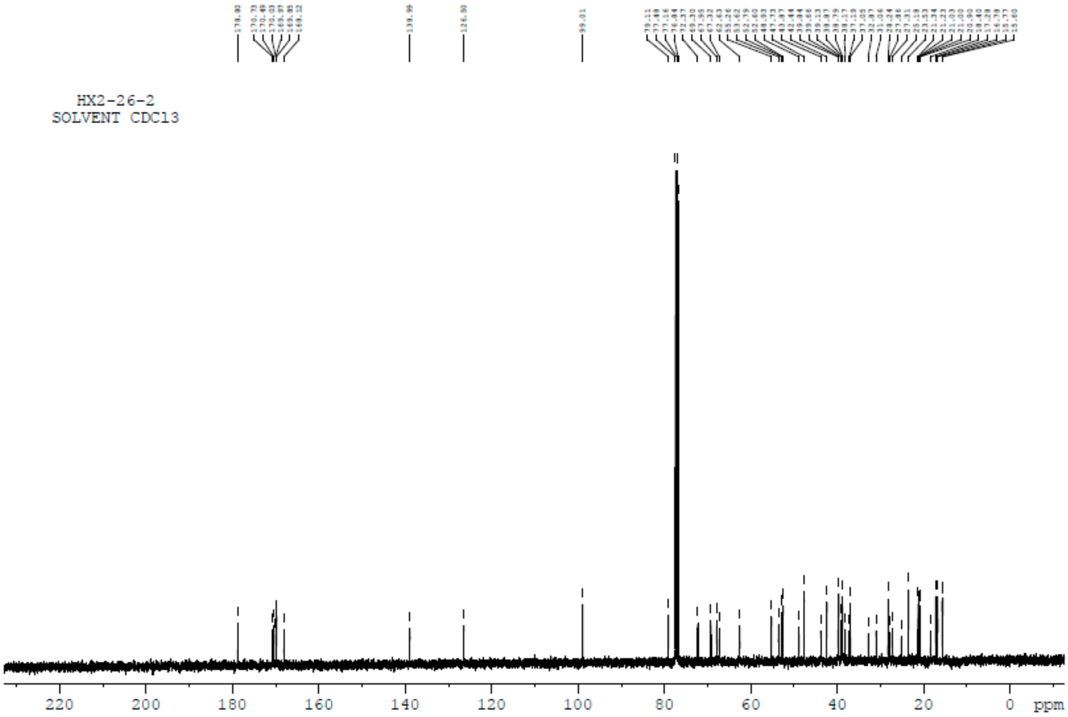

54. HR-ESI-MS spectrum of compound **38**

HIGH RESLUTION MASS SPECTROMETRY REPORT

Sample No.	Formula (M)	Ion Formula	Measured m/z	Calc m/z	Diff (ppm)
HX2-26	C51 H78 N2 O15	C51 H79 N2 O15	959.5472	959.5475	0.34

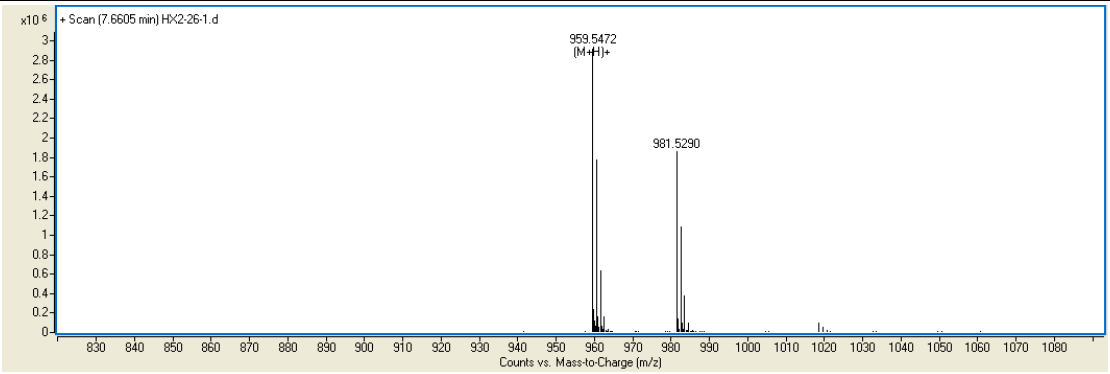

55. ^{1}H NMR (400 MHz, CD_3OD) spectrum of compound **39**

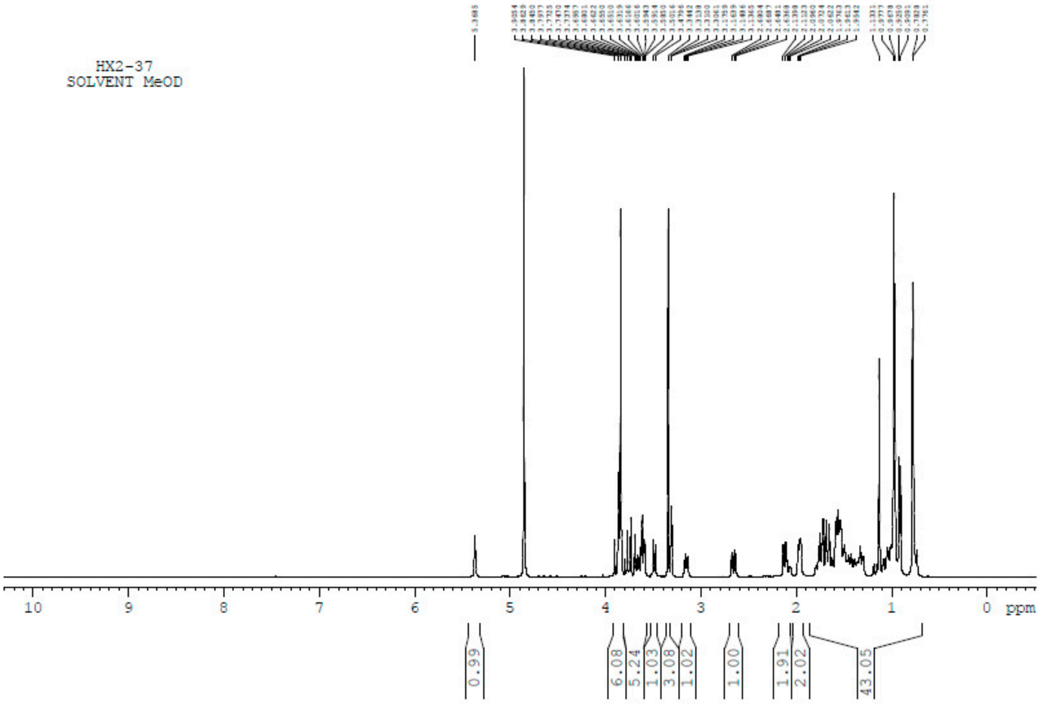

56. ^{13}C NMR (100 MHz, CD_3OD) spectrum of compound **39**

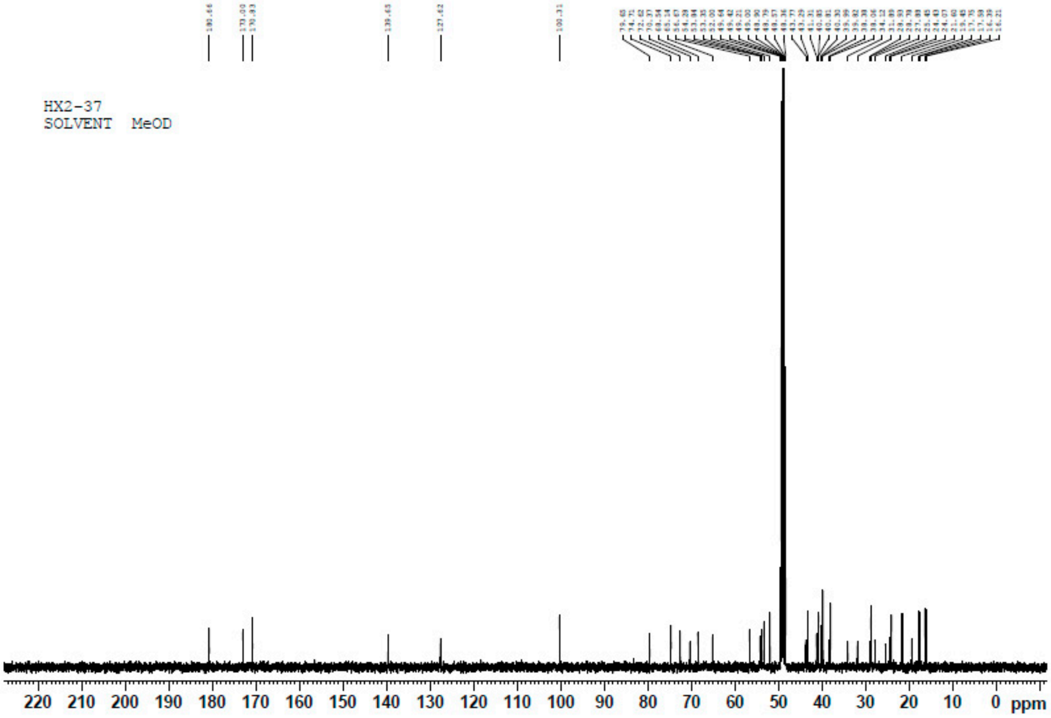

57. HR-ESI-MS spectrum of compound **39**

HIGH RESLUTION MASS SPECTROMETRY REPORT

Sample No.	Formula (M)	Ion Formula	Measured m/z	Calc m/z	Diff (ppm)
HX2-37	C43 H70 N2 O11	C43 H70 N2 Na O11	813.4879	813.4872	-1.05

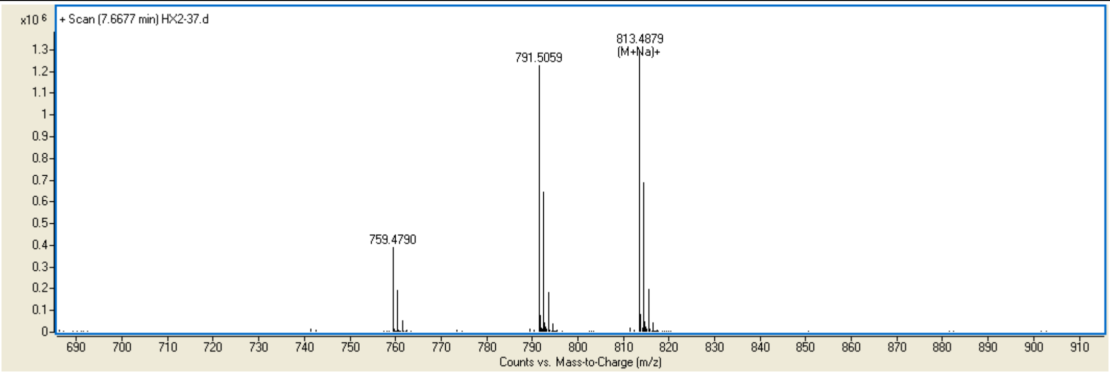

58. ^{1}H NMR (400 MHz, CD_3OD) spectrum of compound **40**

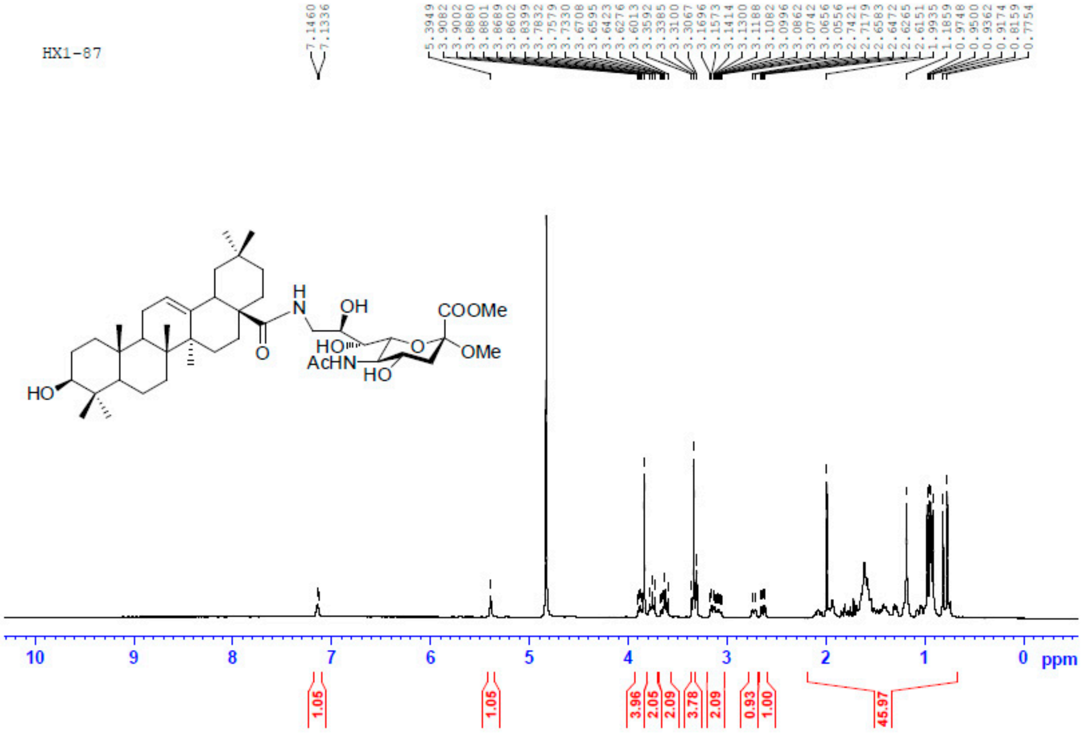

59. ^{13}C NMR (100 MHz, CD_3OD) spectrum of compound **40**

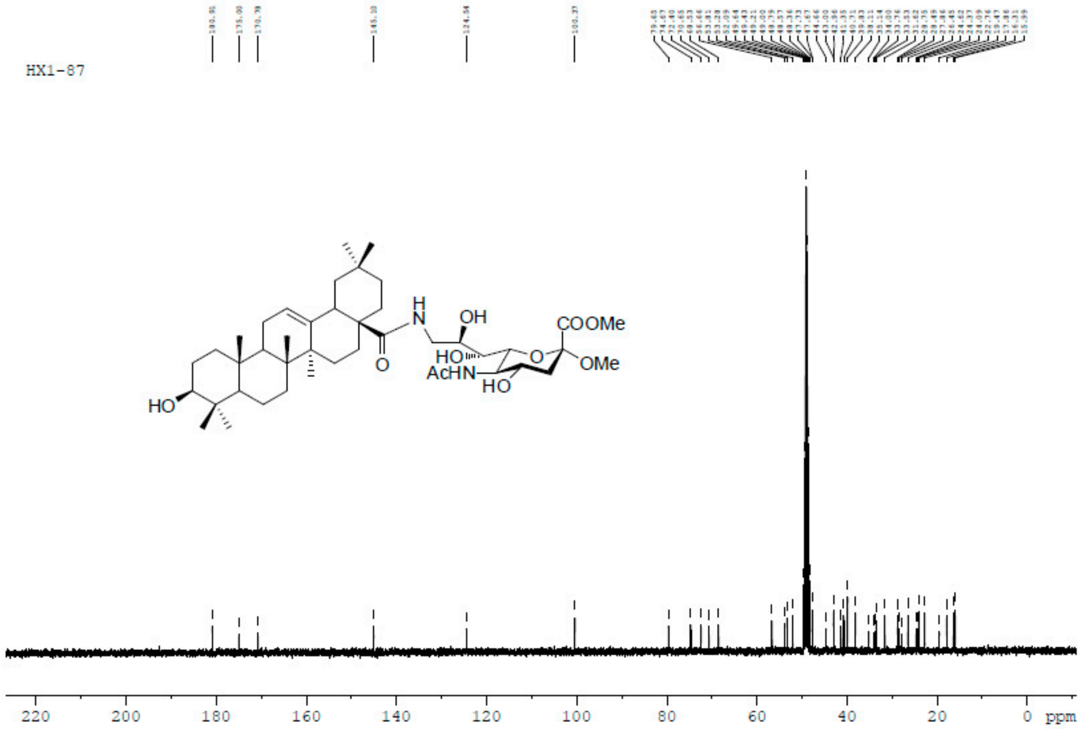

60. HR-ESI-MS spectrum of compound **40**

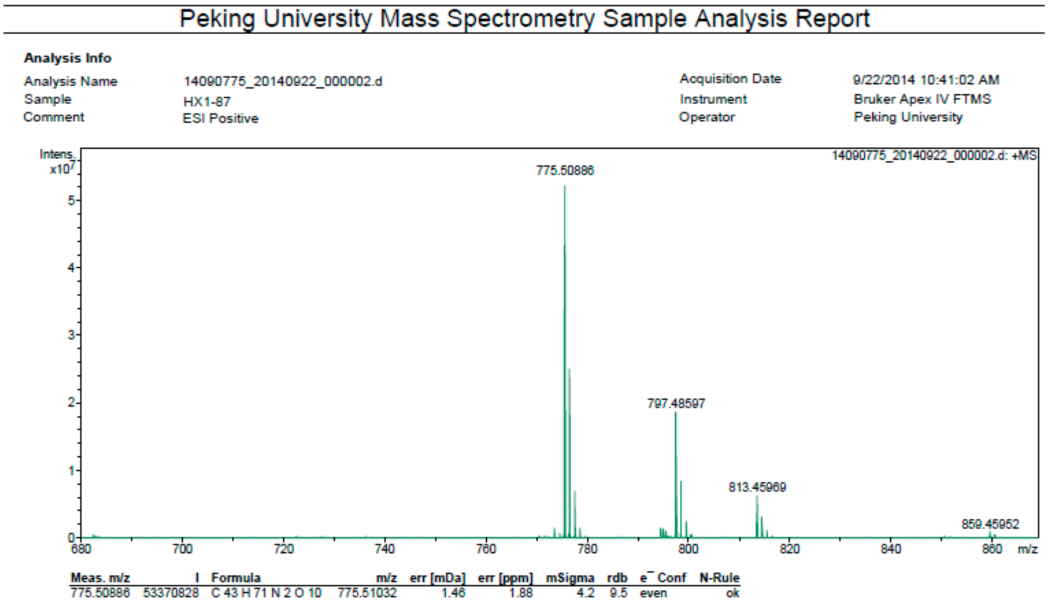

61. ^{1}H NMR (400 MHz, CD_3OD) spectrum of compound **41**

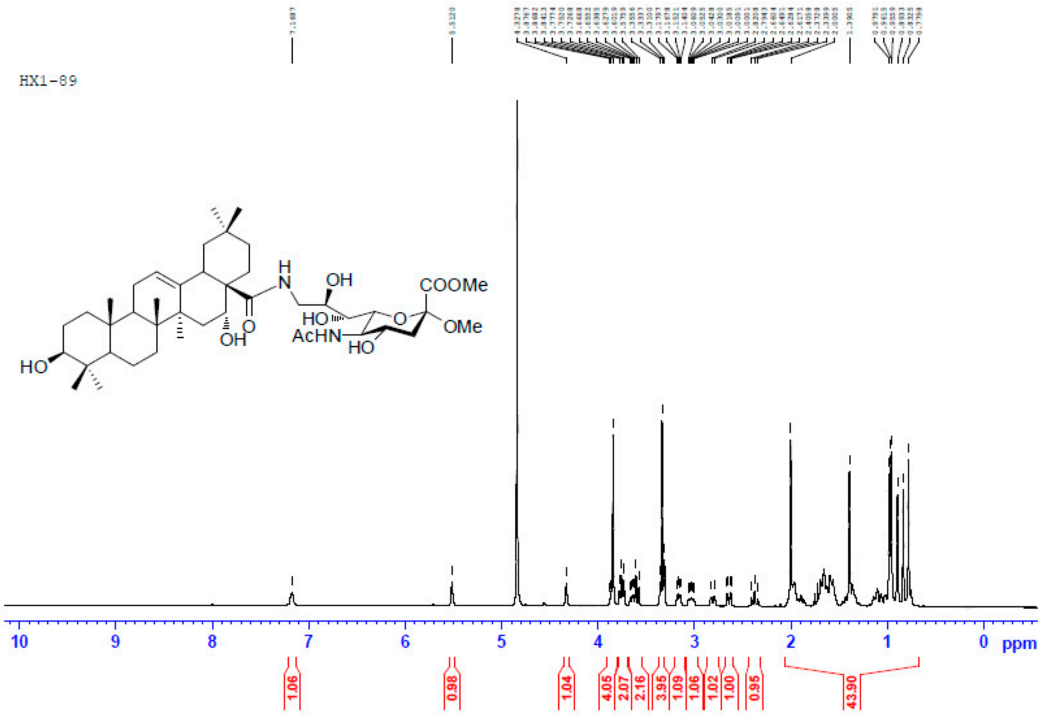

62. ^{13}C NMR (100 MHz, CD_3OD) spectrum of compound **41**

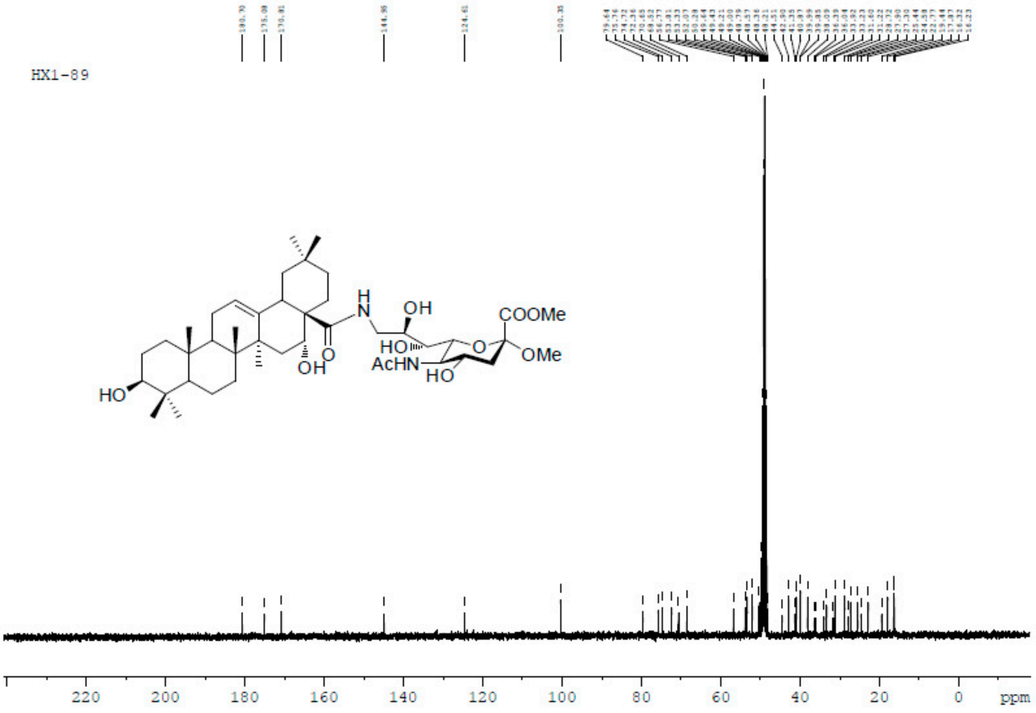

63. HR-ESI-MS spectrum of compound **41**

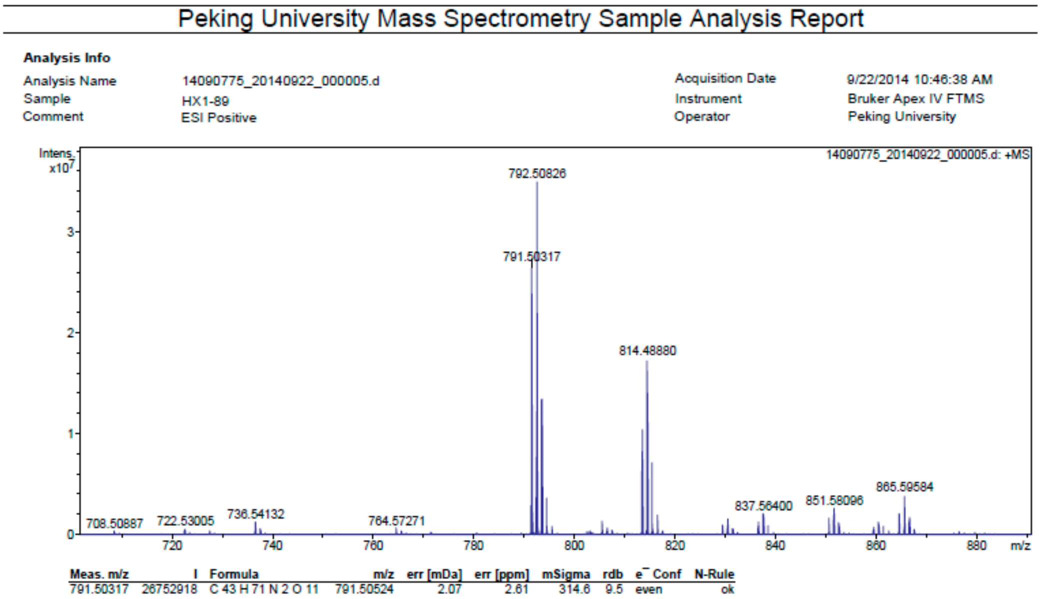

64. ^{1}H NMR (400 MHz, CD3OD) spectrum of compound **42**

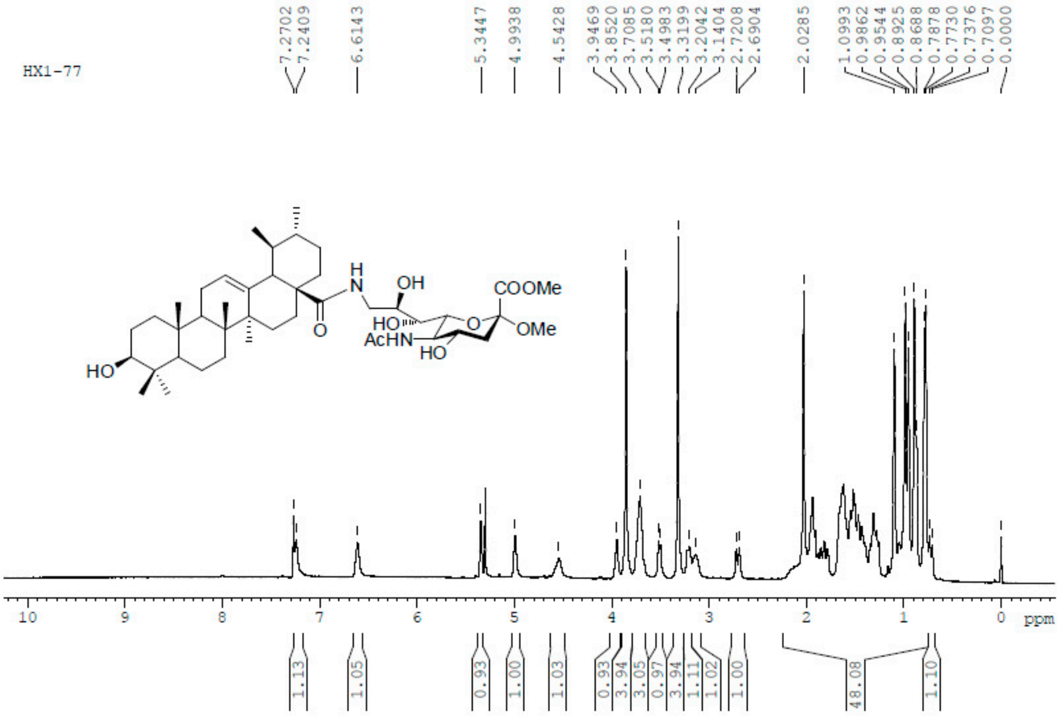

65. ^{13}C NMR (100 MHz, CD_3OD) spectrum of compound **42**

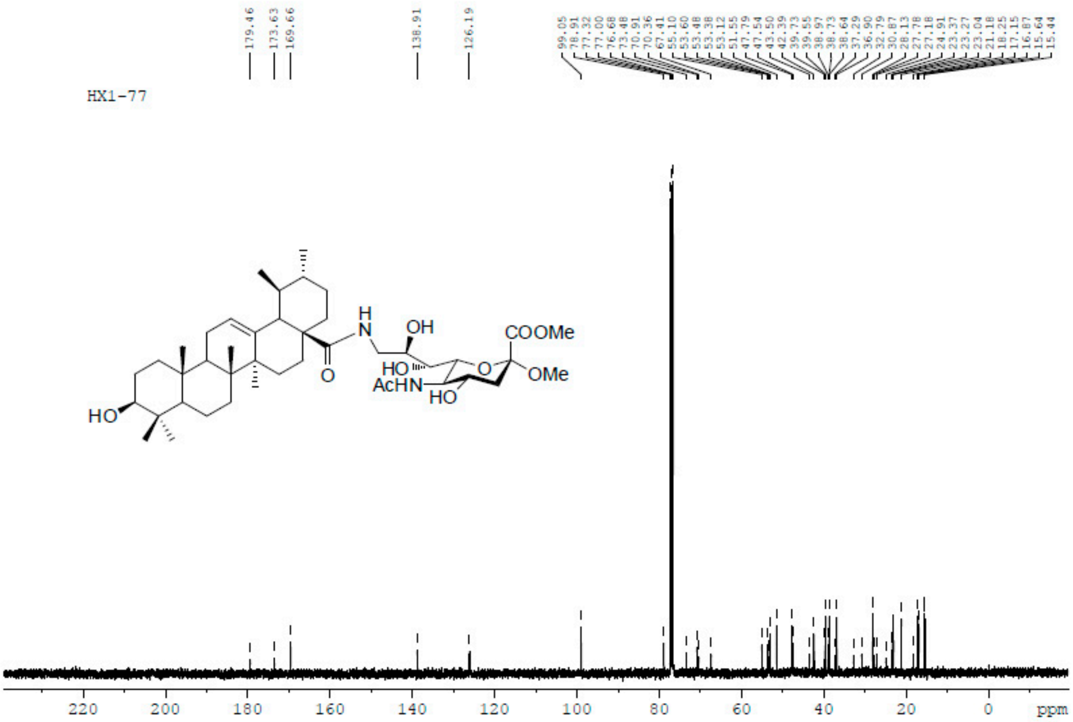

66. HR-ESI-MS spectrum of compound **42**

Peking University Mass Spectrometry Sample Analysis Report

Analysis Info

Analysis Name	14090775_20140922_000001.d	Acquisition Date	9/22/2014 10:38:45 AM
Sample	HX1-77	Instrument	Bruker Apex IV FTMS
Comment	ESI Positive	Operator	Peking University

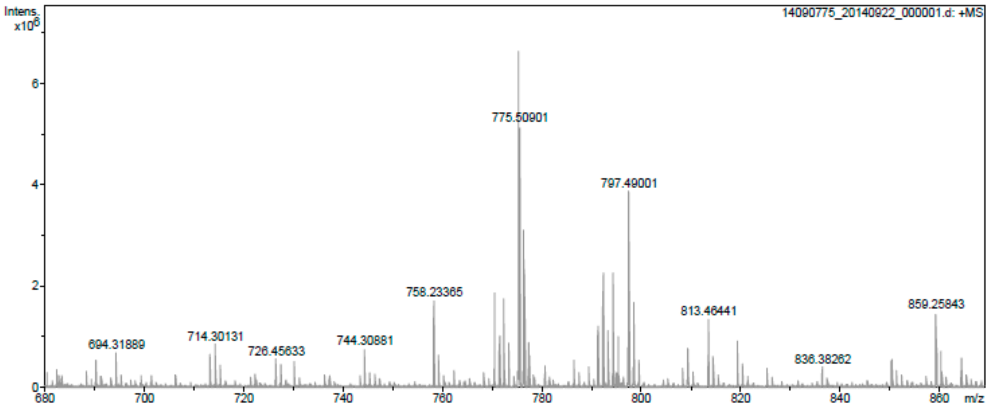

Meas. m/z	I	Formula	m/z	err [mDa]	err [ppm]	mSigma	rdb	e⁻ Conf	N-Rule
775.50901	5183648	C 43 H 71 N 2 O 10	775.51032	1.31	1.69	12.3	9.5	even	ok
797.49001	3892586	C 43 H 70 N 2 Na O 10	797.49227	2.26	2.83	28.2	9.5	even	ok

67. ^{1}H NMR (400 MHz, $CDCl_3$) spectrum of compound **44**

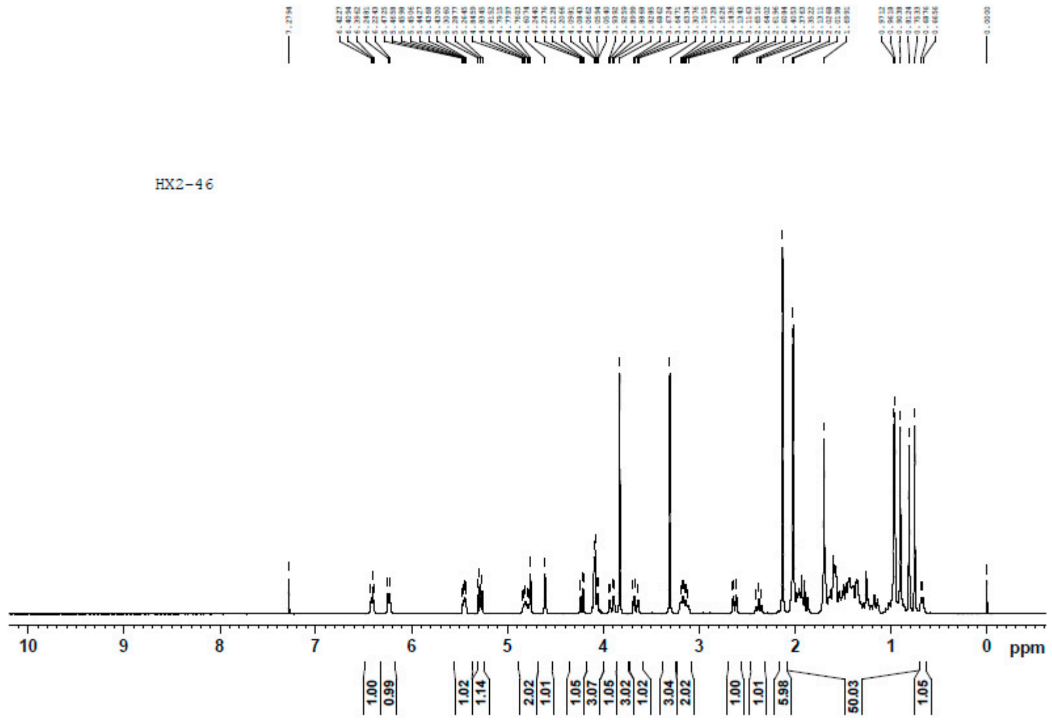

68. ^{13}C NMR (100 MHz, $CDCl_3$) spectrum of compound **44**

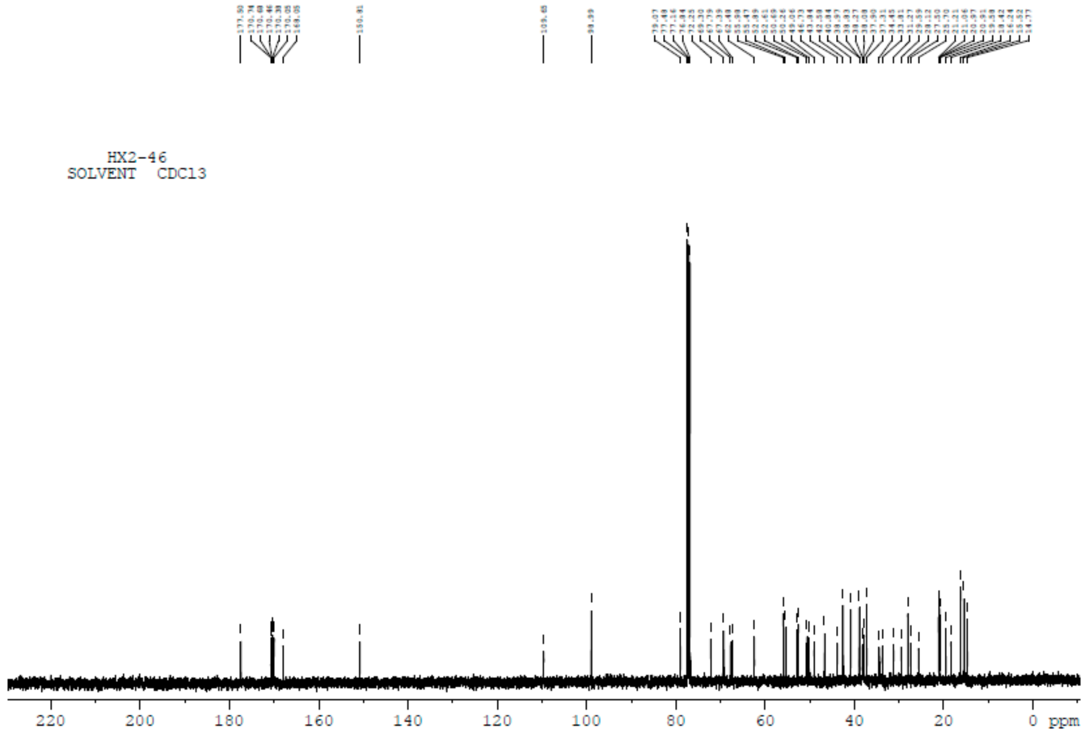

69. HR-ESI-MS spectrum of compound **44**

HIGH RESLUTION MASS SPECTROMETRY REPORT

Sample No.	Formula (M)	Ion Formula	Measured m/z	Calc m/z	Diff (ppm)
HX2-46	C51 H78 N2 O15	C51 H79 N2 O15	959.5481	959.5475	-0.45

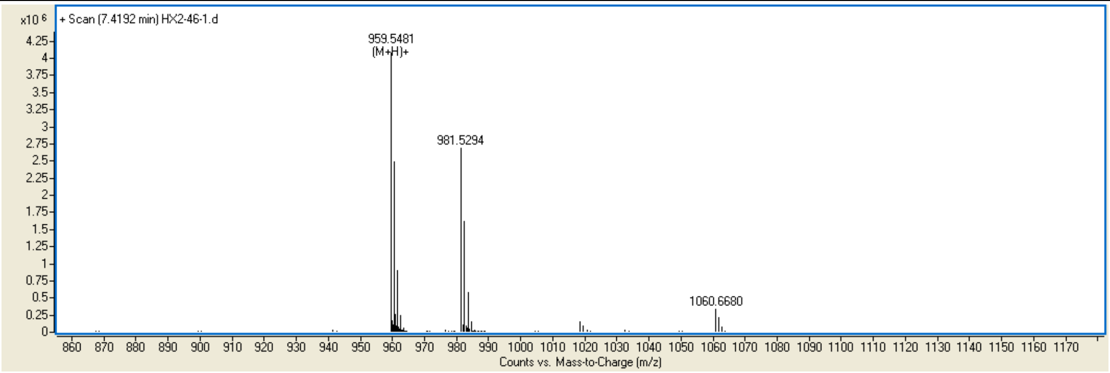

70. ^{1}H NMR (400 MHz, CD_3OD) spectrum of compound **45**

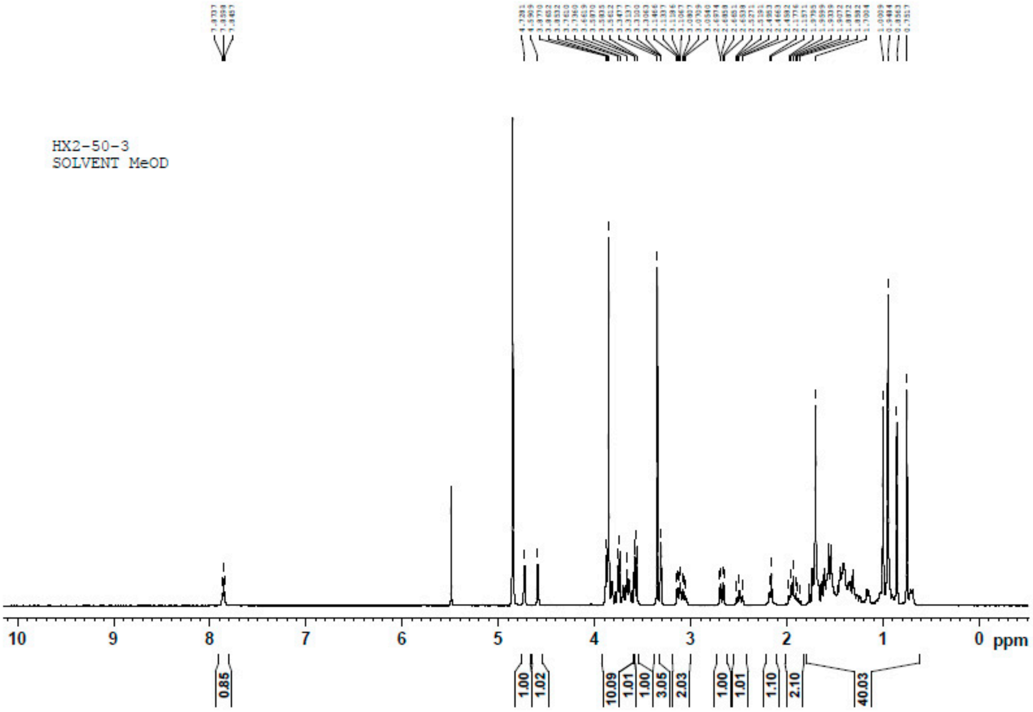

71. ^{13}C NMR (100 MHz, CD_3OD) spectrum of compound **45**

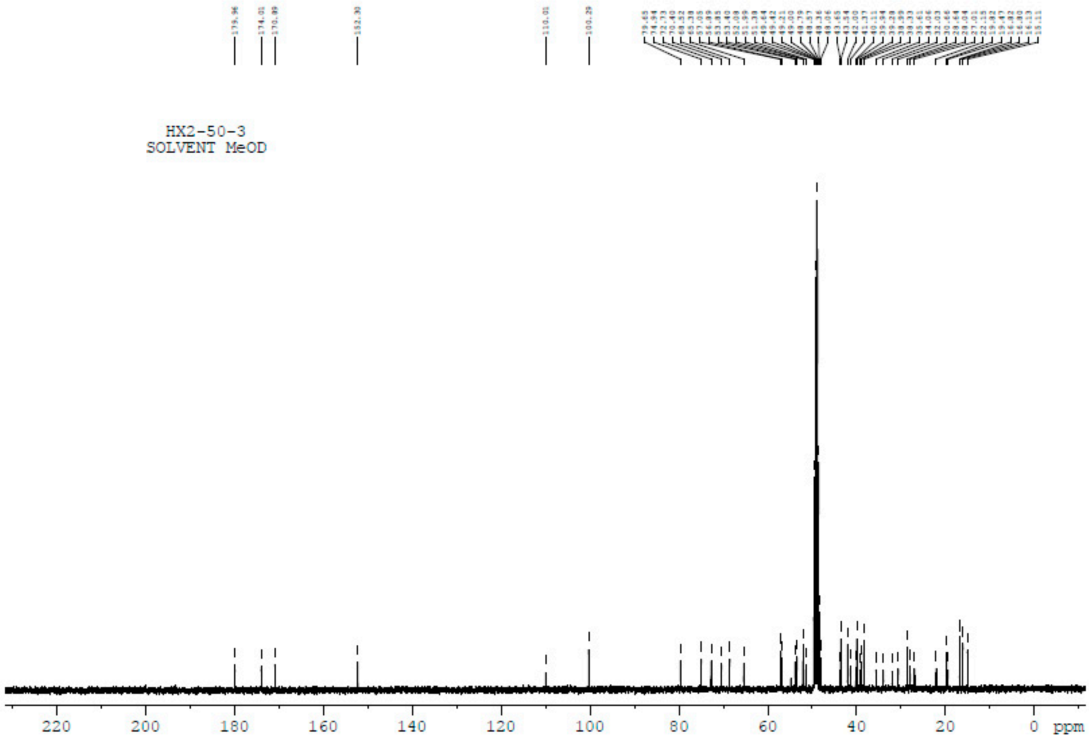

72. HR-ESI-MS spectrum of compound **45**

HIGH RESLUTION MASS SPECTROMETRY REPORT

Sample No.	Formula (M)	Ion Formula	Measured m/z	Calc m/z	Diff (ppm)
HX2-50	C43 H70 N2 O11	C43 H70 N2 Na O11	813.4878	813.4872	-0.82

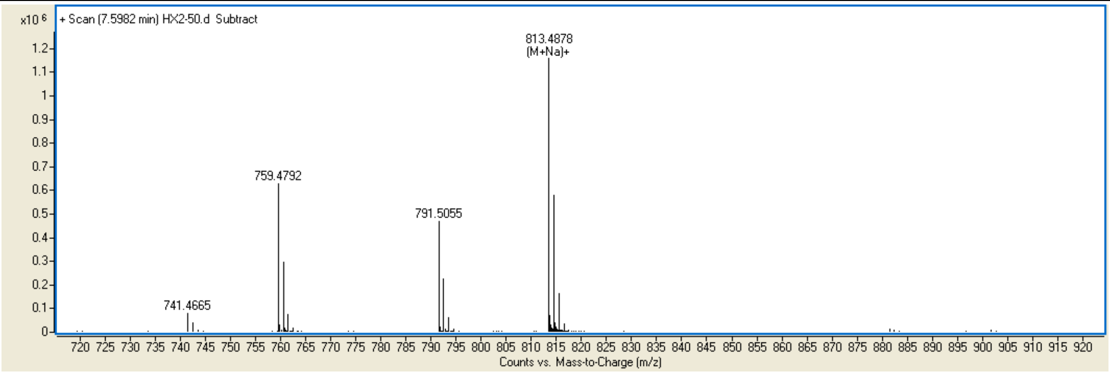

73. ^{1}H NMR (400 MHz, CD_3OD) spectrum of compound **46**

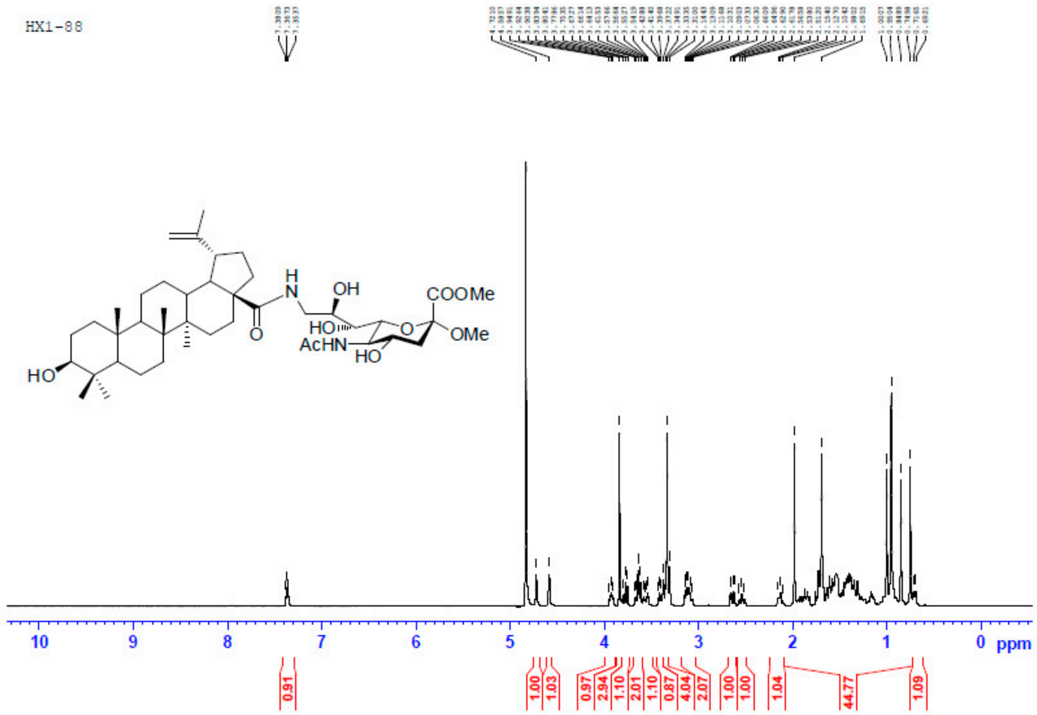

74. ^{13}C NMR (100 MHz, CD_3OD) spectrum of compound **46**

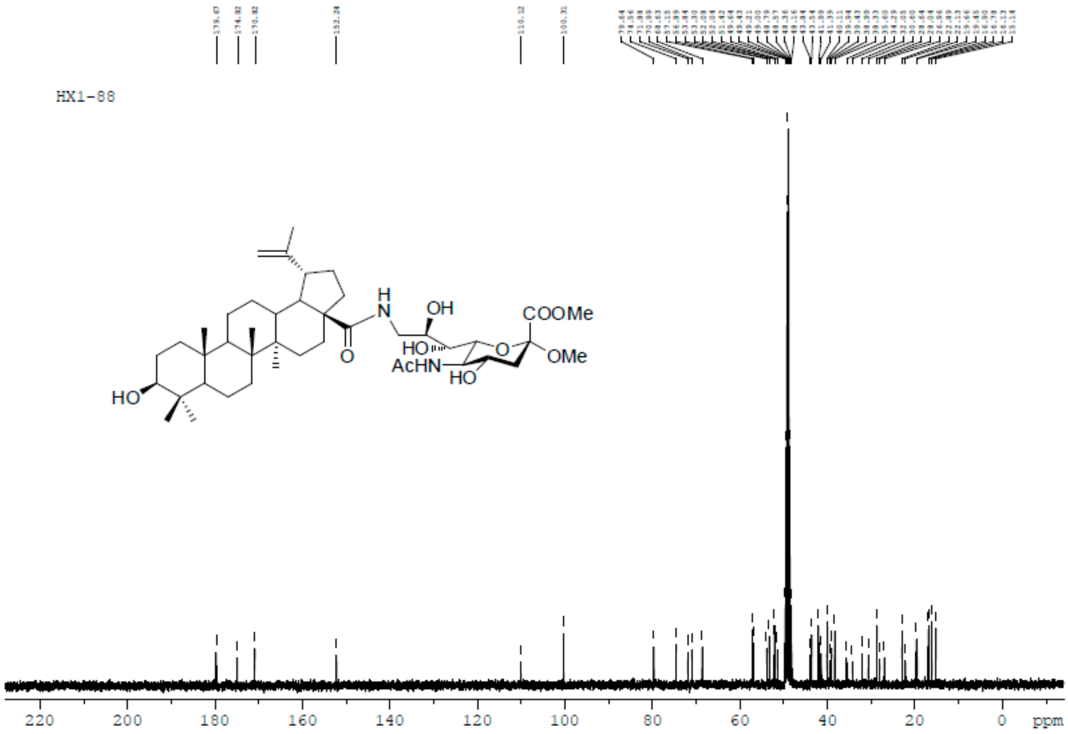

75. HR-ESI-MS spectrum of compound **46**

Peking University Mass Spectrometry Sample Analysis Report

Analysis Info

Analysis Name	14090775_20140922_000003.d	Acquisition Date	9/22/2014 10:42:48 AM
Sample	HX1-88	Instrument	Bruker Apex IV FTMS
Comment	ESI Positive	Operator	Peking University

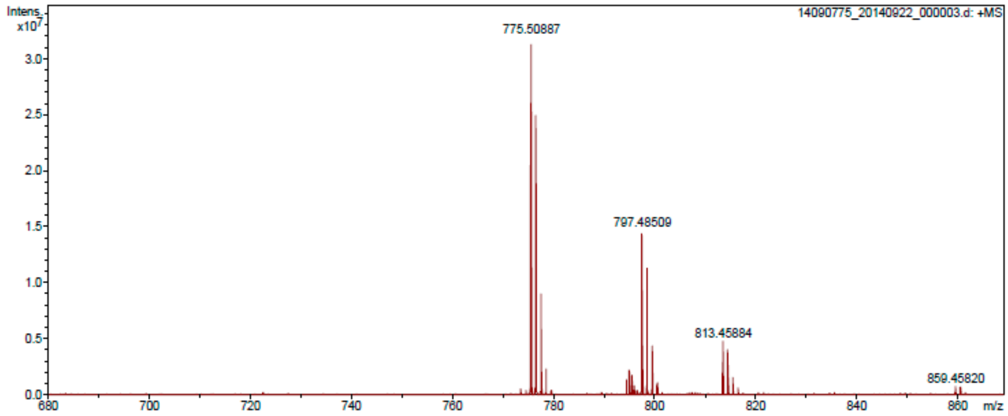

Meas. m/z	I	Formula	m/z	err [mDa]	err [ppm]	mSigma	rdb	e⁻ Conf	N-Rule
775.50887	31970314	C 43 H 71 N 2 O 10	775.51032	1.46	1.88	177.2	9.5	even	ok
